# A bis(silylene)pyridine pincer ligand can stabilize mononuclear manganese(0) complexes: facile access to isolable analogues of the elusive d^7^-Mn(CO)_5_ radical†

**DOI:** 10.1039/d2sc03352f

**Published:** 2022-07-06

**Authors:** Shweta Kalra, Daniel Pividori, Dominik Fehn, Chenshu Dai, Shicheng Dong, Shenglai Yao, Jun Zhu, Karsten Meyer, Matthias Driess

**Affiliations:** Department of Chemistry: Metalorganics and Inorganic Materials, Technische Universität Berlin Strasse des 17. Juni 135, Sekr. C2 D-10623 Berlin Germany matthias.driess@tu-berlin.de; Inorganic Chemistry, Department of Chemistry and Pharmacy, Friedrich-Alexander-Universität Erlangen-Nürnberg (FAU) Egerlandstrasse 1 91058 Erlangen Germany; State Key Laboratory of Physical Chemistry of Solid Surface, Collaborative Innovation Center of Chemistry for Energy Materials (iChEM), College of Chemistry and Chemical Engineering, Xiamen University 361005 Xiamen China

## Abstract

Using the potentially tridentate *N*,*N′*-bis(*N*-heterocyclic silylene)pyridine [SiNSi] pincer-type ligand, 2,6-*N*,*N′*-diethyl-bis[*N*,*N′*-di-*tert*-butyl(phenylamidinato)silylene] diaminopyridine, led to the first isolable bis(silylene)pyridine-stabilized manganese(0) complex, {κ^3^-[SiNSi]Mn(dmpe)} 4 (dmpe = (Me_2_P)_2_C_2_H_4_), which represents an isolobal 17 VE analogue of the elusive Mn(CO)_5_ radical. The compound is accessible through the reductive dehalogenation of the corresponding dihalido (SiNSi)Mn(ii) complexes 1 (Cl) and 2 (Br) with potassium graphite. Exposing 4 towards the stronger π-acceptor ligands CO and 2,6-dimethylphenyl isocyanide afforded the related Mn(0) complexes κ^2^-[SiNSi]Mn(CO)_3_ (5) and κ^3^-[SiNSi]Mn(CNXylyl)_2_(κ^1^-dmpe) (6), respectively. Remarkably, the stabilization of Mn(0) in the coordination sphere of the [SiNSi] ligand favors the d^7^ low-spin electronic configuration, as suggested by EPR spectroscopy, SQUID measurements and DFT calculations. The suitability of 4 acting as a superior pre-catalyst in regioselective hydroboration of quinolines has also been demonstrated.

## Abstract 2

An isolable bis(silylene)pyridine stabilized manganese(0) complex {κ^3^-[SiNSi]Mn(dmpe)}, isolobal to elusive Mn(CO)_5_ radical has been synthesized and fully characterised.

## Graphical abstract

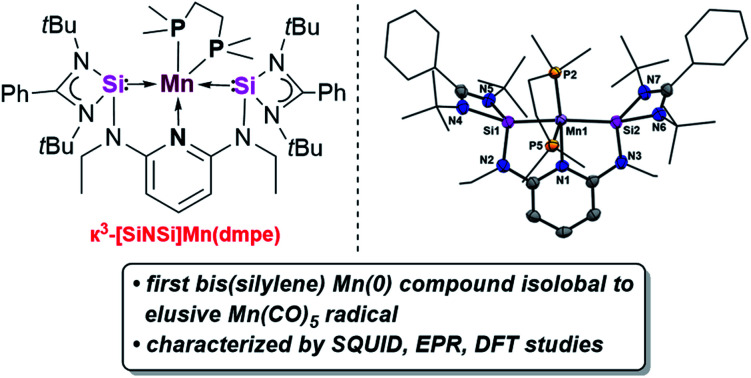

## Introduction

Silylene-based transition-metal (TM) complexes have proven to be a remarkable asset in molecular functional chemistry because of their fascinating and unusual chemical properties.^1^ Mono-silylenes have been extensively used to stabilize a wide variety of metal complexes in the zero oxidation state.^2^ The bidentate analogues, bis(silylenes), are also known to serve as effective ligands to stabilize even the first-row metal centers Fe, Co, Ni in low oxidation states.^3–6^ Despite numerous reports on silylene–TM(0) complexes, examples of Mn(0) complexes are rare. A previous attempt to attain a silylene–Mn(0) complex using the Mn(0) precursor Mn_2_(CO)_10_ failed and resulted in a disproportionation reaction to form a silylene–Mn(i) complex.^7^

**Fig. 1:**
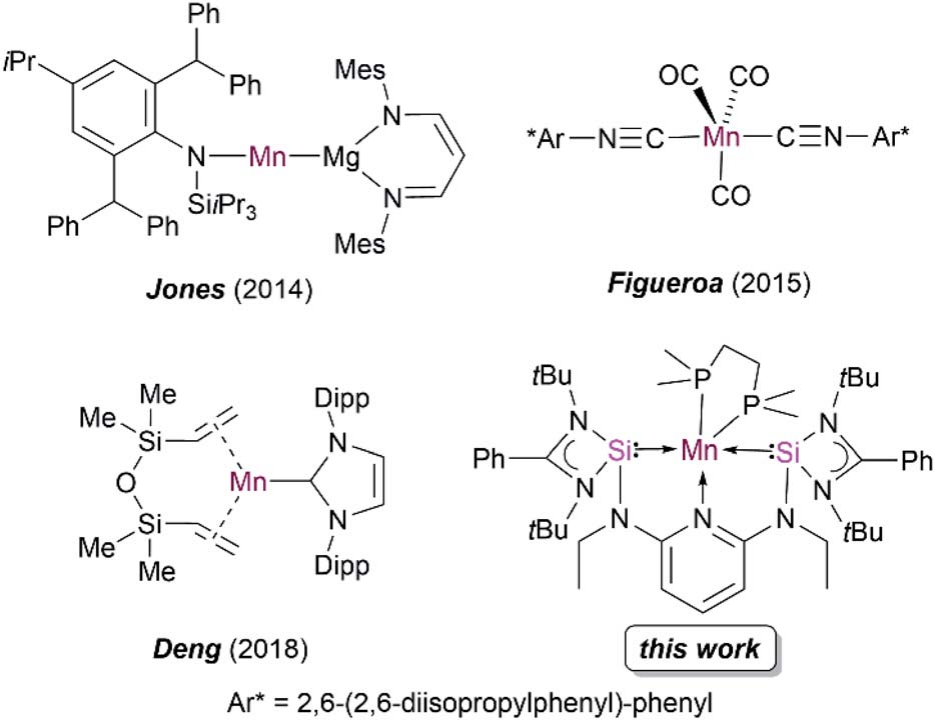
Reported examples of low-valent manganese complexes and the novel bis(silylene) Mn(0) complex of this work.

Early attempts to isolate the five-coordinated 17 valence electron (VE) Mn(CO)_5_ monoradical have only been limited to their isolation in low-temperature matrices.^8^ The synthesis of open shell Mn(CO)_5_ and Mn(CO)_3_(PR_3_)_2_ complexes with Mn(0) have also been probed *via* photochemical substitution of dinuclear manganese complexes [Mn_2_(CO)_8_L_2_] (L = CO, phosphine),^9^ but their high reactivity has thus far prevented isolation at room temperature. Recently, bulky amido and nacnac–Mg(i) ligands (nacnac = *N*-acetyl-*N*-acetonates) have also been employed to stabilize Mn(0) complexes (Fig. 1).^10^ Figueroa and co-workers have ingeniously utilized the strategy of Mn(−i) and Mn(+i) comproportionation reaction along with employing sterically crowded isocyanide ligands to isolate a five-coordinate Mn(0) complex (Fig. 1).^11^ Additionally, Deng and co-workers utilized a *N*-heterocyclic carbene (NHC) and dvtms (1,3-divinyltetramethyldisiloxane) ligands to obtain Mn(0) complexes, although the mononuclear complexes formed are stable only at low temperatures (Fig. 1).^12^ Previously, our group reported the synthesis of a neutral bis(silylene)pyridine ligand [SiNSi] that has been used to stabilize Fe(0) in [SiNSi]Fe(PMe_3_)_2_; the latter turned out to act as a suitable pre-catalyst for the outer-sphere hydrosilylation of ketones.^13,14^ While a series of dichlorido bis(silylene) Mn(ii) complexes have been synthesized and studied to be suitable pre-catalysts for stereoselective transfer hydrogenation of alkynes,^15^ as mentioned above, a bis(silylene) Mn(0) complex is currently unknown in the family of TM compounds. Keeping in mind that low-valent TM complexes with dative Si(ii)–TM(0) bonds have a versatile potential in metal-mediated catalytic transformation of organic substrates,^4,16^ we became interested in using the potentially tridentate 2,6-*N*,*N*′-diethyl-bis[*N*,*N*′-di-*tert*-butyl(phenylamidinato)silylene] diaminopyridine ligand (SiNSi) to realize 17 VE Mn(0) complexes being stable at room temperature, starting from corresponding Mn(ii) precursor complexes. Herein, we report the isolation, electronic structure, spectroscopic characterisation and reactivity of the first isolable bis(silylene)-stabilized mononuclear d^7^-Mn(0) complexes.

## Results and discussion

Starting with the [SiNSi] ligand and MnCl_2_, the bis(silylene) dichloride manganese(ii) complex κ^2^-[SiNSi]MnCl_2_ (1) has been synthesized by a modified procedure (see ESI†). The characterisation data obtained for 1 is in agreement with the literature report.^15^ Complex 1 was further characterised by electron paramagnetic resonance (EPR) spectroscopy and SQUID magnetometry (SQUID = superconducting quantum interference device). With *S* = 5/2, high-spin Mn(ii), d^5^ electronic configuration in tetrahedral geometry (e^2^, t^3^_2_), in complex 1, as expected an effective magnetic moment of 6.10 B.M. was determined by the variable-temperature (VT) SQUID magnetometry (Fig. S1†).

**Fig. 2:**
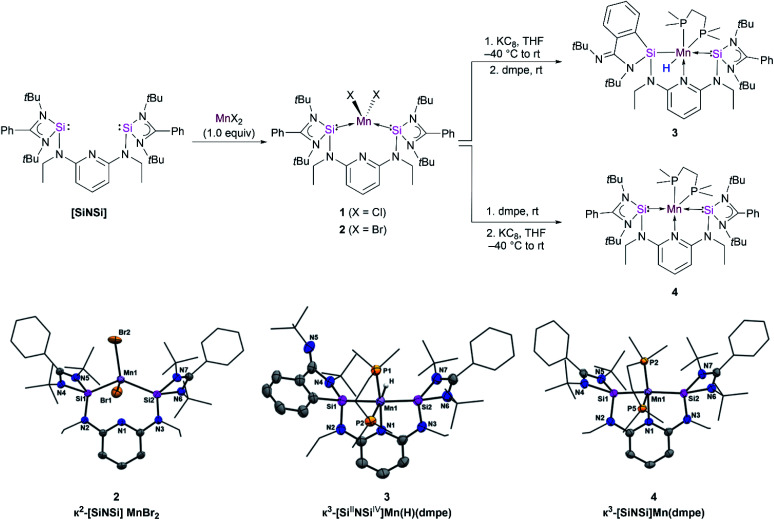
Synthetic routes to compound 3 and 4 (top) and molecular structures of 2, 3 and 4 (bottom). Molecular structures have been depicted with thermal ellipsoids at 50% probability. Hydrogen (except hydrogen atom bonded to metal atom) and solvent atoms are omitted for clarity. Selected distances (Å) and angles (°) for 2: Mn1–Si1 2.560(7), Mn1–Si2 2.567(6), Br1–Mn1–Br2 108.837(15). Selected bond lengths (Å) and angles (°) for 3: Mn1–Si1 2.320(8), Mn1–Si2 2.213(8), Mn1–N1 2.076(2), Mn1–P1 2.221(7), N1–Mn1–Si2 82.04(6), N1–Mn1–P2 95.55(6); selected bond lengths (Å) and angles (°) for 4: Mn1–Si1 2.242(13), Mn1–N1 2.111(3), Mn1–P2 2.176(11), N1–Mn1–Si1 80.27(10), P2–Mn1–Si2 96.04(5).

Similarly, the reaction of [SiNSi] with MnBr_2_ led to formation of the homologous dibromide manganese(ii) complex 2 in 90% yields as light-yellow powder (Fig. 2, top). The molecular structure established by single-crystal X-ray diffraction analysis shows that the Mn center in 2 adopts a distorted-tetrahedral coordination geometry with Mn–Si distances of 2.560(7) and 2.567(6) Å, respectively, which are consistent with those observed in previously reported bis(silylene) Mn(ii) complexes (Fig. 2, bottom).^15^ The lack of pyridine coordination to the Mn center in 1 and 2 is presumably due to the enhanced σ-donor strength of the silylene donors of the [SiNSi] ligand.^17^ The stronger σ-donor nature of the bis(silylene) arms rather forces manganese to adopt tetrahedral coordination over five-fold coordination. This result is in stark contrast with the existing Mn(ii) complexes of PNP^18^ and NNN type^19^ of pincer ligands, in which the *N*-pyridine coordination is widely observed.

The effective magnetic moment of 2 was measured in solution (Evans method, *d*_8_-THF, 298 K) and in the solid-state by SQUID magnetometry (on two independently synthesized and measured samples). The solution moment of 5.81 B.M. compares well with the 5.99 B.M. determined at 300 K by SQUID, and are in good agreement with the calculated spin-only value for a high-spin d^5^ ion (*S* = 5/2, *μ*^s.o.^_eff_ = 5.93 B.M.) (Fig. S5†).^20,21^ As anticipated, the magnetic moment of 2 is temperature independent in the 150–300 K range. Below 150 K, *μ*_eff_ decreases to reach 5.0 B.M. at 2 K, which is likely due to zero-field splitting (ZFS) in a high-spin Mn(ii) complex. The EPR spectrum of 2 (Fig. S6†) exhibits a highly rhombic *S* = 5/2 spectrum with resonances between 50 and 600 mT, corresponding to *g*_eff_ = 10–1. Further spectral features in the low-field region, *i.e.* a six-line-pattern, may stem from partially resolved hyperfine coupling to one ^55^Mn (*I* = 5/2, 100% nat. abundance) nucleus (Fig. S6†). The magneto-chemical properties of the Cl-derivative 1 are similar, and shown in Fig. S1 and S2.†

Taking inspiration from bis-carbene and bis-phosphine Fe complexes [CNC]Fe(N_2_)_2_ (ref. 22) and [PNP]Fe(N_2_)_2_ (ref. 23) reported by Chirik and co-workers, we attempted to isolate the similar manganese–dinitrogen complex [SiNSi]Mn(N_2_)_2_. However, the reductive dehalogenation of 1 and 2 with potassium graphite under an N_2_ atmosphere in the absence of supporting ligand resulted in oily residues from which we could not isolate a new product. This result prompted us to employ an additional ligand that would allow us to isolate the desired product. Hence, the reductions of 1 and 2 were carried out in the presence of dmpe (1,2-bis-dimetylphosphinoethane) in THF solutions (Fig. 2, top). Dark red crystals of a product suitable for an X-ray crystallographic analysis could be grown in concentrated hexane solutions of the reaction mixture. The diffraction analysis indicated the formation of the hydrido silyl-silylene-pyridine Mn(ii) complex [Si^II^NSi^IV^]Mn(H)(dmpe) 3 (Fig. 2, bottom) with a distorted octahedral coordination geometry around the Mn center. The mechanism is unknown, but we propose that 3 is formed *via in situ* formation of a Mn(0) species which can undergo a silylene-assisted intramolecular phenyl C–H activation and silylation of the phenyl ring at the amidinate ligand. Subsequently, oxidative addition of the Si–H moiety of the phenylhydrosilyl group to Mn(0) affords the hydrido silyl manganese(ii) complex 3. The IR spectrum of 3, featuring a Mn–H stretching vibrational band located at *ν* = 1745 cm^−1^ (Fig. S7†), confirms the presence of a Mn–H moiety. It is noteworthy that a similar silylene-assisted C–H activation of a phenyl group has previously been reported for a bis(silylene)-stabilized Pd(0) complex.^24^

Complex 3 displays two different Mn–Si distances, 2.320(8) and 2.213(8) Å for Mn1–Si1 and Mn1–Si2, respectively. The longer one corresponds to the change of the oxidation state of Si1 (+IV). However, the Mn1–Si1 distance is similar to the value observed for the hydrido silyl manganese complex [CpMn(dmpe)(H)(SiPh_2_H)] (2.319(4) Å) reported by Sun *et al.*^25^ The shorter Mn1–Si2 distance, in turn, is longer than the reported value of 2.148(2) Å in the related hydrido silylene manganese(i) complex *cis*-[(dmpe)_2_MnH(=SiPh_2_)] by Emslie and co-workers, presumably due to the presence of a two-coordinate silylene ligand and Mn(i).^26,27^

The isolation of 3 encouraged us to change the order of addition of the starting materials to possibly achieve isolation of the proposed Mn(0) intermediate. In fact, addition of dmpe to 1 prior to the reduction step with KC_8_ enabled the isolation of the new paramagnetic species κ^3^-[SiNSi]Mn(dmpe) 4 with a Mn(0) center in 39% crystalline yields (Fig. 2, top). Using 2 as a precursor affords 4 in higher yield (50%). The high resolution electrospray mass spectrum (HR-ESI-MS) of 4 unambiguously confirmed its composition ([M + 2H]^+^: calcd 888.4629; exptl. 888.4618). Dark purple, diamond-shaped crystals of 4, suitable for a single-crystal X-ray diffraction analysis, were obtained in concentrated hexane solutions. The Mn center features a pseudo-square pyramidal (PSQP) coordination geometry (with the *τ*_5_ geometry index of 0.41)^28^ (Fig. 2, bottom). A similar geometric surrounding of Mn was observed in the pentacoordinate Mn(0) complex [Mn(CO)_3_(CNAr*)_2_] (Ar* = 2,6-(2,6-diisopropylphenyl)-phenyl) reported by Figueroa and co-workers (Fig. 1).^11^ The PSQP geometry of 4 includes a *N*-pyridine coordination to Mn, featuring a pincer-type configuration, coplanar with the Si(ii) donor atoms and one of the P(iii) atoms of dmpe, leaving one P atom in axial position. The Mn–Si distances of 2.214(13) and 2.242(13) Å in 4 are considerably shorter than those in 1 and 2, which range from 2.592(5) to 2.635(7) Å. This observation illustrates an increased electron density at Mn(0) which, in turn, results in more π-backdonation to the empty 3p-orbitals at Si(ii) and causes a stronger Mn–Si bond interaction. Furthermore, it is worth mentioning that 4 features an average axial/basal-plane angle of 96.4°. Interestingly, an axial/basal-plane angle of 96(±3)° was determined by IR spectroscopy for the transient [Mn(CO)_5_] isolated in a CO matrix at 20 K.^8,29^

**Fig. 3:**
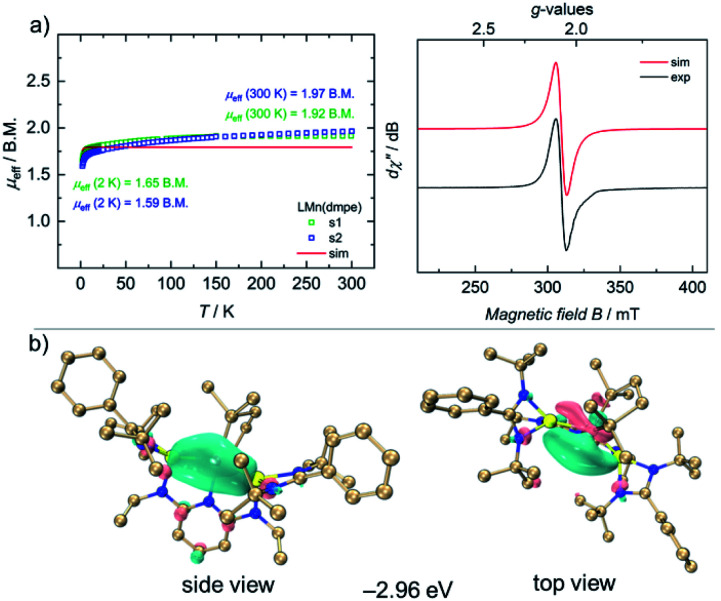
(a) VT SQUID magnetization data (left) with added simulation (red trace) for a *S* = 1/2 and *g*_avg_ = 2.07 system, and CW X-band EPR spectrum of 4 (right, black trace) and its simulation (red trace) (b). Highest singly occupied molecular orbitals (HSOMO) of 4.

Complex 4 is stable for several days at room temperature in the solid state as well as in solutions under inert gas atmospheres. The ^1^H NMR spectrum of 4 shows paramagnetically shifted signals from −20 to +30 ppm. The magnetic moment of 2.65 B.M. for 4 was observed by the Evans method in C_6_D_6_ solutions at 298 K. To gain more insights into the magnetic properties of 4 in the solid-state, VT SQUID measurements were performed on microcrystalline samples of 4. Fig. 3a (left) shows the effective magnetic moment (*μ*_eff_) of 4, plotted as a function of temperature. At 300 K, 4 shows a magnetic moment of 1.95 B.M., slightly higher than the spin-only-value for one unpaired electron (*μ*^s.o.^_eff_ = 1.73 B.M.), which essentially reflects the presence of a single-unpaired electron in d^7^ electronic configuration of a low-spin Mn(0) ion with an *S* = 1/2 doublet ground state. Accordingly, the EPR spectrum of 4 in THF features a broad, isotropic signal with an effective *g*-value of *g*_iso_ = 2.07, consistent with the SQUID measurement (Fig. 3a, right).

To gain further insights into the electronic nature at the Mn(0) center in complex 4, density functional theory (DFT) calculations were performed at PBE0-D3BJ/Def2-SVP-ma-TZVP level of theory. The principal interacting orbital (PIO) analysis determines the strength of orbital interactions *via* PIO-based bond index (PBI).^30,31^ PIO analysis developed by Lin was an intuitive bonding analysis tool by dominant interacting semi-localized orbitals between fragments. Accordingly, principal interacting spin orbitals (PISO) analysis extends the PIO analysis to open-shell systems.^32^

Compound 4 has a doublet ground state (Table S10†) with an unpaired electron. The unpaired spin density is mainly localized on the d orbital of the Mn atom along the Mn–P bond as shown in the highest singly occupied orbital (HSOMO) of 4 (Fig. 3b). The α and β interactions of Mn–Si, Mn–N and Mn–P1 bonds exhibit very similar patterns. Specifically, the first α- and β-PISO pairs with similar PBI values (0.19 and 0.22) suggest a dative bond between Mn and Si1, with contributions of 0.92 e from Mn and 1.45 e from Si1 in total (Fig. S36†), similar to those of the Mn–Si2 bond (Fig. S37†). Apart from the first α- and β-PISO pairs, the second α- and β-PISO pairs illustrate a π-backdonation from d orbitals of Mn atom to vacant p orbitals of Si1 atom (Fig. S37†). As for Mn–P bonds, there are one σ-type donation and two π-type backdonation in both α and β systems (Fig. S38 and S39†). The dominating contribution of σ-type donation also suggests a dative interaction from each P atom to Mn. In addition, the unpaired α electron occupying the d orbital oriented along the Mn–P2 bond makes the Mn atom in α system more difficult to accept the electron donation from the P2, resulting in a smaller PBI value of the first α-PISO than that of the first β-PISO pair (0.08 *vs.* 0.26, Fig. S39†). Similarly, the primary donation interaction represented *via* the first α- and β-PISO pairs indicates a weak dative bond between Mn and N1 atoms (Fig. S40†) indicated by smaller PBI values.

## Reactivity

Given the unique structure of 4 as a bis(silylene)pyridine Mn(0) complex, we were curious about its reactivity towards stronger π-acceptor ligands, such as CO and xylyl isocyanide, to access other [SiNSi]Mn(0) species. The treatment of toluene solutions of 4 with CO gas (1 bar) at ambient temperature resulted in a rapid colour change from dark-purple to wine-red, indicating the formation of the new Mn(0) complex κ^2^-[SiNSi]Mn(CO)_3_ (5). The IR spectrum of the latter shows three distinctive CO bands at 1844, 1811 and 1716 cm^−1^, verifying the addition of three CO ligands to Mn (Fig. S13†). The IR absorption peaks are shifted to relatively lower energy compared to known isolobal [Mn(CO)_3_(CNAr^Dipp^)_2_],^11^ presumably owing to the more pronounced σ-donation of Si(ii) *vs.* CO and hence a significant increase of Mn → CO π back-donation.

**Fig. 4:**
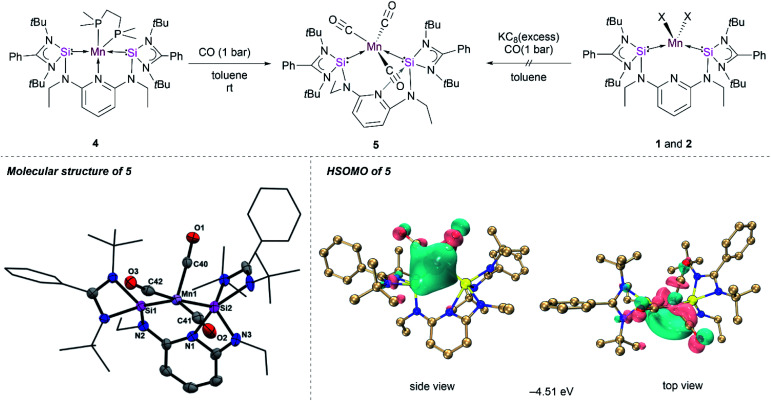
Synthesis, molecular structure, and HSOMO of 5. Thermal ellipsoids were drawn at 40% probability. Hydrogen and solvent atoms are omitted for clarity. Selected bond lengths (Å) and angles (°) for 5: Mn1–Si1 2.224(18), Mn1–Si2 2.362(18), Mn1–C40 1.774(7), Mn1–C41 1.801(7), Mn1–C42 1.778(7), Si1–Mn1–Si2 93.85(6), C42–Mn1–Si2 174.4(2).

Single-crystals suitable for an X-ray diffraction analysis were obtained from slow diffusion of diethyl ether into benzene solutions of 5. The molecular structure of 5 revealed a pseudo-square pyramidal coordination geometry (*τ* = 0.13)^28^ of Mn with one of the CO in the axial position (Fig. 4), which is reminiscent of the geometry predicted for the elusive Mn(CO)_5_ radical.^27^ It is important to note that the structural orientation of the bis(silylene) donors plays a crucial role in preventing the dimerization of the Mn(0) radical species on exposure to the CO atmosphere. However, Mn–Mn dimerization occurs in the case of a NHC Mn(0) complex (Fig. 1) reported by Deng *et al.*^12^ The molecular structure of 5 showed slightly shorter Mn–C bond distances of 1.801(7) Å, 1.774(7) Å and 1.778(7) Å compared to its bis(isocyanide) analogue manganese(0) complex [Mn(CO)_3_(CNAr^Dipp^)_2_],^11^ owing to the strong σ-donation from the [SiNSi] pincer ligand. The carbonyl ligands are known to carry a negative charge in the neutral metal–carbonyl complexes,^33^ which further explains the shortening of Mn–C bond distances in 5. Interestingly, the molecular structure of 5 shows that the pincer ligand rearranges to give a Si1–Mn1–Si2 bond angle close to 90° through the loss of the N(pyridine) coordination (Fig. 4); instead, a N(pyridine) coordination to one of the Si(ii) centers is realized. This has not previously been observed in the related Fe(0) complex [SiNSi]Fe(CO)_2_, bearing same ligand.^13^ However, a similar N(pyridine) → Si(ii) coordination was observed in the tetracarbonyl Fe(0) Ge(0) complex (‘germylone’) [SiNSi]Ge(0) → Fe(CO)_4_, with the same [SiNSi] pincer ligand.^34^

To gain insights into the electronic structure of 5, PISO analyses were performed and we found that compound 5 also has a doublet ground state (Table S11†), with an unpaired electron, which is also predominantly localized on the d orbital of the Mn along the Mn–C bond (Fig. 4). Thus, it is reasonable to obtain the almost same interaction of the Mn–Si bonds as those of compound 4 (Fig. S41 and S42†). In addition, one σ type and two π type interactions were also found for Mn–C bonds in both α system and β system (Fig. S43–S45†), similar to those of Mn–C bonds in compound Mn(CO)_5_ (Fig. S47 and S48†).^35^ The σ donation interaction from the C atom to the Mn atom is strongest according to the largest PBIs of the first α- and β-PISO pairs. These two π type backdonation are stronger than those of Mn–P bonds, based on the larger PBI values (such as 0.04 *vs.* 0.11), leading to a stronger Mn–C bond. The σ-type donation from the lone pair electrons of the N1 atom to the Si2 atom is confirmed by the PISO analysis.

The solution-state magnetic moment, determined by the Evans method (C_6_D_6_, 298 K), was found to be 1.80 B.M., suggesting the presence of a Mn(0) ion with d^7^ low-spin electronic configuration and an *S* = 1/2 ground state in 5. In contrast, in the solid-state, highly temperature-dependent and reproducibly low SQUID magnetization data were observed, with *μ*_eff_ values ranging from 0.5 B.M. at 2 K to 1.18 B.M. at 300 K (Fig. S16†). Regardless, in line with the *S* = 1/2 spin-state, the EPR spectrum of a benzene solution of 5, recorded at 293 K, features one broad resonance centered at *g*_eff_ ≈ 2.01 with a partly-resolved, six-line spectrum due to hyperfine coupling to the nuclear spin of a single Mn nucleus (^55^Mn, *I* = 5/2, 100% nat. abundance, Fig. S15†).

**Scheme 1:**
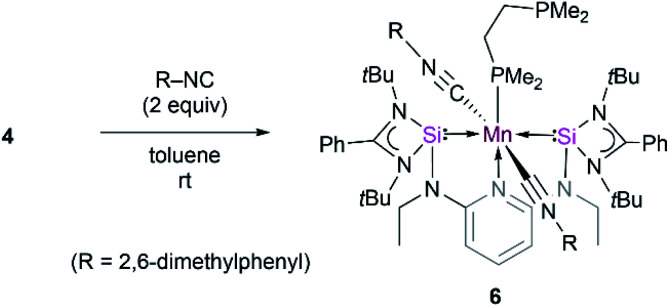
Reactivity of 4 towards 2,6-xylyl isocyanide to form 6.

Further attempts to synthesize 5 through reduction of 1 and 2 in the presence of CO were unsuccessful. This could be explained by the uncontrollable reactivity of *in situ* formed Mn(0) species. This finding underlines the advantage of 4 being a suitable precursor for other Mn(0) complexes. Accordingly, the dmpe ligand ligated to the d^7^-Mn(0) center in 4 can also partially be replaced by the 2,6-dimethyl phenyl isocyanide ligand. When toluene is added to a mixture of 4 and 2,6-xylyl isocyanide in the molar ratio of 1 : 2, green solutions of the new κ^3^-[SiNSi]Mn(CNXylyl)_2_(dmpe) complex 6 are formed (Scheme 1). Complex 6 displays IR stretching vibration bands at 1900 and 1866 cm^−1^ which account for the coordination of two xylyl isocyanide ligands at Mn (Fig. S17†). The presence of two isocyanide ligands in 6 is corroborated by its HR-APCI-MS spectrum ([M]^+^: calcd 1148.5943; exptl. 1148.5951) (Fig. S18†).

The magnetic behavior of solid samples of 6 were studied by VT-SQUID measurements, which, similar to samples of 5, feature highly reproducible, temperature-dependent moments, ranging from 1.00 B.M. at 2 K to 1.84 B.M. at 300 K, thus confirming the presence of a low-spin d^7^-Mn(0) ion in the doublet (*S* = 1/2) ground state (Fig. S19†). This is in agreement with the magnetic moment determined by the Evans method (1.86 B.M., C_6_D_6_, 298 K). EPR spectra of 6, recorded in frozen and liquid solutions at 9, 95 and 293 K, in a variety of solvents (*e.g.* benzene, toluene, THF), show complicated signals centered around *g* ≈ 2. All spectra reproducibly display additional, partly resolved spectral features, likely due to hyperfine coupling to the ^55^Mn and, possibly, ^31^P, nuclei (^55^Mn : *I* = 5/2, 100%; ^31^P : *I* = 1/2, 100%). However, a proper simulation has not been accomplished (Fig. S20–S23†).

## Catalytic suitability of 4 in hydroboration of quinolines

In the past decade, regioselective dearomatization of *N*-heteroarenes has been widely studied due to the importance of partially reduced 1,2-dihydro- and 1,4-dihydroborated products in agrochemical and pharmaceutical industries.^36^ Moreover, the catalysts used to carry out the transformation previously mostly involved precious transition metal such as Rh,^37^ La^38^ and Th.^39^ In recent years, various novel approaches also have been employed utilizing Fe,^40^ Ni,^41^ and Zn^42^ complexes.

We then set out to learn whether hydroboration of *N*-heteroarenes can be achieved using the Mn complexes. To the best of our knowledge, Mn-catalysed selective hydroboration of *N*-heteroarenes is unknown. This encouraged us to investigate the catalytic ability of isolated bis(silylene) Mn(0) complexes towards hydroboration of *N*-heteroarenes. A preliminary catalyst screening showed that 4 acts as an active pre-catalyst for hydroboration of 7a with pinacolborane (HBpin) exhibiting high regioselectivity towards 1,2-dihydroborated product at 50 °C (Table 1, entry 3). This perhaps could be explained based on the considerable labile nature of dmpe ligand as observed in complex 4. In comparison to the CO and xylyl isocyanide ligands in complex 5 and 6 respectively, even higher reactivity was observed with complex 4. With the optimized reaction conditions in hand, we expanded the substrate scope to various electronically modified quinolines. Both electron withdrawing substituents such as Cl-, Br-, F- and electronic donating group such as methyl group at various positions were well-tolerated (Table 2). Notably, hydroboration reactions of 7b and 7c were more selective when performed with catalyst 4 than with Mn(0) precursor Mn_2_(CO)_10_ under the standard condition with same catalyst loadings.

**Table 1:** Catalyst screening for regioselective 1,2-hydroboration of quinoline^a^

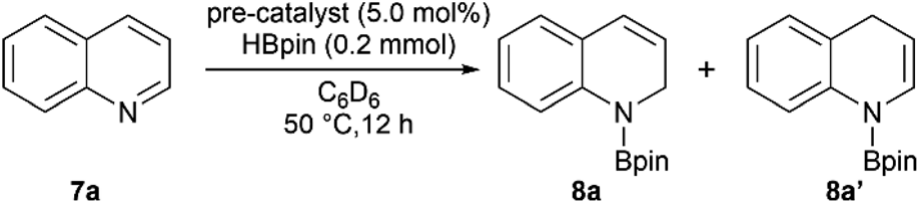		
Entry	Pre-catalyst	Conv.^b^ (%) (8a : 8a′)
1	[SiNSi]MnCl_2_1	<5
2	[SiNSi]MnBr_2_2	<5
3	[SiNSi]Mn(dmpe) 4	97 (76 : 24)
4	[SiNSi]Mn(CO)_3_5	17 (85 : 15)
5	[SiNSi]Mn(CNXyl)_2_(dmpe) 6	30 (90 : 10)

a All reactions were performed in 0.1 mmol scale.

b Conversion was determined from ^1^H NMR spectroscopy using mesitylene as internal standard.

**Table 2:** Catalytic hydroboration of *N*-heteroarenes with HBpin using κ^3^-[SiNSi]Mn(dmpe) (4) as a precatalyst^a^

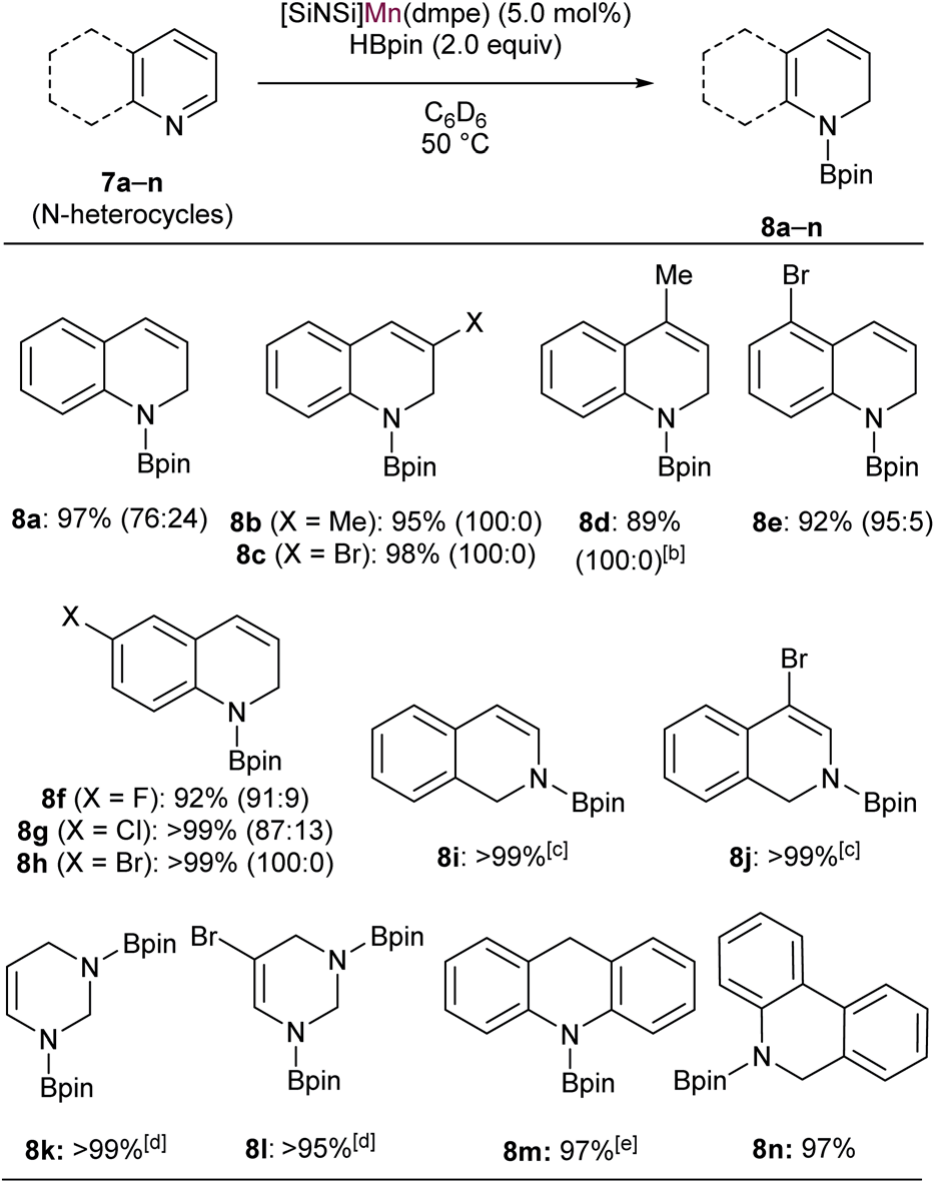

a All reactions were performed in 0.1 mmol scale. Conversion was determined from ^1^H NMR spectroscopy using mesitylene as internal standard. Regioisomeric ratio (1,2- *vs.* 1.4-addition products) ratio has been shown in parentheses.

b For 36 h.

c At room temperature (2 h).

d 4.0 equiv. of HBPin was used.

e For 48 h.

When performed with 3-methylquinoline (7b), under standard conditions 1,2-hydroboration product 8b was formed exclusively in the presence of complex 4 (8b : 8b′ = 100 : 0) than with Mn_2_(CO)_10_ (8b : 8b′ = 90 : 10). Similar results were also obtained when 3-bromoquinoline (7c) was used as substrate (8c : 8c′ = 100 : 0 with catalyst 4 than 85 : 15 with Mn_2_(CO)_10_). These outcomes further affirmed the catalytic suitability of complex 4 towards regioselective hydroboration of *N*-heterocycles compared to Mn_2_(CO)_10_. Complex 4 also catalyzes the hydroboration of other *N*-heterocycles such as isoquinoline, pyrimidine, acridine and phenanthridine under similar condition. However, complex 4 did not show any reactivity towards quinolines with substituents such as Cl-, Br- and Ph-groups at 2- and 8-positions (not shown). The reason can probably be attributed to the steric hindrance introduced by the bulkier groups in the proximal position of active catalyst.

**Fig. 5:**
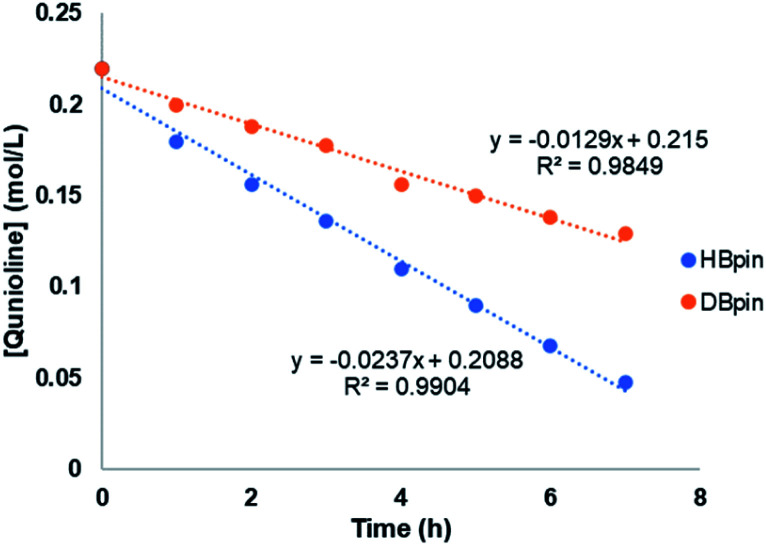
Kinetic isotope effect (KIE) experiment.

To gain more insights of the mechanistic pathway, deuterium-labelling experiments were conducted, which in turn resulted in selective 1,2-deuteroboration of quinoline as major product with 73% deuterium incorporation (Fig. 5). Kinetic isotope effect (KIE) experiments revealed a *K*_H_/*K*_D_ of 1.84, indicating the dissociation of H–Bpin bond to be a rate determining step. The values obtained are comparable to the KIE study for reaction of catecholborane (HBcat) with ruthenium complexes by Hartwig *et al.*^43^ Interestingly, when an equimolar reaction of complex 4 with HBpin was carried out at 50 °C, no expulsion of dmpe ligand was observed in the ^31^P NMR confirming the phosphine to be an integral part of active catalyst. The control reaction of 4 with HBpin resulted in the formation of diamagnetic Mn–H species as indicated by the appearance of hydridic signal at −9.6 ppm in ^1^H NMR of reaction mixture (Fig. S29†). ^1^H–^31^P HMQC NMR further indicated the correlation of hydridic signal at −9.6 ppm with the phosphorous atom present in the proposed 4a complex. Consecutive addition of 3-methylquinoline to it afforded selectively the 1,2-hydroboration product in high yields and regenerated the active catalyst as shown *via* emergence of the hydridic signal in the ^1^H NMR spectrum. However, attempts to isolate the proposed active catalyst were unsuccessful. Further, to rule out the formation of any active borenium species catalysing the reaction, a control experiment was performed using dmpe (5.0 mol%) and HBpin (2.0 equiv.) under optimized conditions in the absence of 4, but no conversion was observed.

**Scheme 2:**
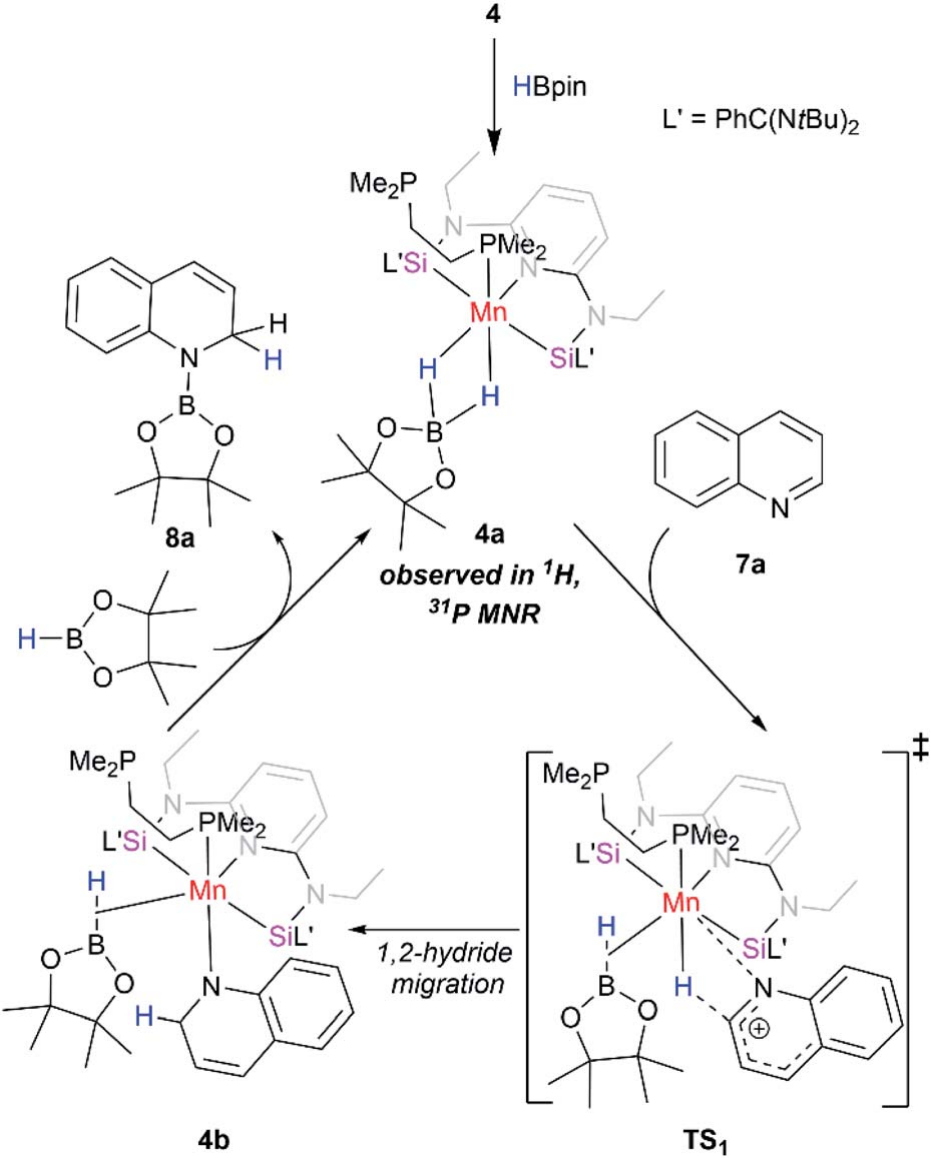
Proposed reaction pathway for hydroboration of quinoline with HBpin using [SiNSi]Mn(dmpe) (4) as a precatalyst.

Based on these findings, we propose a plausible reaction mechanism for the catalytic pathway as shown in Scheme 2. The reaction was initiated by the *in situ* reaction of 4 with 2.0 equiv. of HBpin molecule to afford a diamagnetic Mn(i) complex 4a through the loss of one phosphine coordinated to Mn center. The active catalyst 4a once formed, undergoes a 1,2-hydride migration to quinoline *via* a four-membered transition state. The intermediate 4b on reaction with HBpin results into 1,2-hydroborated quinoline and regenerates back the active intermediate 4a. The proposed mechanism is in analogy with the related reports on regioselective hydroboration of *N*-heteroarenes.^42^

## Conclusion

In summary, we have demonstrated the synthesis of the first bis(silylene) stabilized pincer type manganese (0) complex coordinated by three different sets of supporting ligands such as phosphine, CO and isocyanide. The complexes were studied by EPR spectroscopy and SQUID magnetometry referring to the presence of zero-valent manganese with one unpaired electron. The complexes were also examined for the regioselective-hydroboration of *N*-heteroarenes. Complex 4 has shown promising results in the selective hydroboration catalysis when compared to complex 5 and 6. The investigation of these complexes towards new chemical transformations is currently under investigation.

## Data availability

All experimental and computational data associated with this work are available in the ESI.†

## Author contributions

S. K. carried out the synthetic experiments and analyzed the experimental data. D. P. and D. F. collected the variable-temperature magnetic data. C. D. and S. D. performed the DFT calculations. S. Y. assisted the XRD refinement of the compounds and edited the manuscript. J. Z., K. M., and M. D. supervised the work and edited the manuscript. The manuscript was written through contribution of all authors.

## Conflicts of interest

There are no conflicts to declare.

## Supplementary Material

SC-013-D2SC03352F-s001

SC-013-D2SC03352F-s002
