# Supplementary material for: A bis(silylene)pyridine pincer ligand can stabilize mononuclear manganese(0) complexes: facile access to isolable analogues of the elusive d7-Mn(CO)5 radical
Source: Chem Sci. 2022 Jul 6;13(29):8634–41. doi: 10.1039/d2sc03352f (PMC9337724; doi:10.1039/d2sc03352f)
Supplement: SC-013-D2SC03352F-s001 [file SC-013-D2SC03352F-s001.pdf]

Electronic Supplementary Material (ESI) for Chemical Science.
This journal is © The Royal Society of Chemistry 2022

A Bis(silylene)pyridine Pincer Ligand Can Stabilize Mononuclear Manganese(0) Complexes: Facile Access to Isolable Analogues of the Elusive d^7-$Mn(CO)_5$ Radical

Shweta Kalra,[a] Daniel Pividori,[b] Dominik Fehn,[b] Chenshu Dai,[c] Shicheng Dong,[c] Shenglai Yao,[a] Jun Zhu,[c] Karsten Meyer,[b] and Matthias Driess,*[a]

[a]*Institute of Chemistry, Metalorganics and Inorganic Materials, Technische Universität Berlin, Strasse des 17. Juni 135, Sekr. C2, 10623 Berlin, Germany*

[b]*Inorganic Chemistry, Department of Chemistry and Pharmacy, Friedrich-Alexander-Universität Erlangen Nürnberg (FAU), Egerlandstrasse 1, 91058 Erlangen, Germany*

[c]*State Key Laboratory of Physical Chemistry of Solid Surface and Collaborative Innovation Center of Chemistry for Energy Materials (iChEM), and College of Chemistry and Chemical Engineering, Xiamen University, 361005 Xiamen, (People's Republic of China)*

Contents

**1**	**General considerations**	**1**
**2**	**Experimental section**	**3**
**2.1**	**Syntheses and Spectroscopic Data of Reported Compounds**	**3**
2.1.1	Compound [SiNSi]$MnCl_2$ (**1**)	**3**
2.1.2	Compound [SiNSi]$MnBr_2$ (**2**)	**5**
2.1.3	Compound [$Si^{II}NSi^{IV}$]Mn(H)(dmpe) (**3**)	**7**
2.1.4	Compound [SiNSi}Mn(dmpe) (**4**)	**9**
2.1.5	Compound [Si,Si']$Mn(CO)_3$ (**5**)	**12**
2.1.6	Compound [SiNSi]$Mn(XylNC)_2$(dmpe) (**6**)	**15**
**3**	**Experimental section for selective catalytic hydroboration of N-heteroarenes**	**19**
3.1	Optimization of Reaction Conditions	**19**
3.2	Preparative-scale reaction	**20**
3.3	Mercury-test experiment	**21**
3.4	General procedure of catalytic hydroboration of N-heteroarenes with HBpin using [SiNSi]Mn(dmpe) (**4**) as a precatalyst	**21**
3.5	Spectroscopic data of catalytic hydroboration of N-heteroarenes with HBpin using [SiNSi]Mn(dmpe) (**4**) as a precatalyst	**22**
3.6	Mechanistic studies	**27**
**4**	**Cyclic-Voltammetry experiment**	**33**
**5**	**NMR spectra**	**34**
**6**	**X-ray crystallographic data**	**49**
**7**	**Computational details**	**57**
**8**	**References**	**99**

1. General considerations

All experiments and manipulations were carried out using standard Schlenk techniques or in an MBraun inert atmosphere glovebox under dry oxygen free nitrogen atmosphere. Hexane, Et_2O, toluene and THF were dried by standard methods. Benzene-d_6 and THF-d_8 were stirred over a sonicated potassium mirror for a period of 24 h and recondensed into a Schlenk tube containing activated 4 Å mol sieves. The starting material 2,6-*N,N'*-diethyl-bis[*N,N'*-di-tert-butyl(phenylamindinato)silylene] amidopyridine [SiNSi] was prepared according to literature procedure.[1] Potassium graphite was prepared by reacting potassium with previously dried graphite in a 1:8 ratio at 160 °C for 2 h under dried nitrogen. $MnCl_2$, $MnBr_2$ were purchased from Acros Organics. 1,2-Bis(dimethylphosphino)ethane was purchased from abcr GmbH. 2,6-Dimethylphenyl isocyanide was bought from Alfa Aesar. NMR spectra were recorded on a Bruker AV 400 or 500 Spectrometer. The ^{1}H and $^{13}C\{^{1}H\}$ NMR spectra were referenced to the residual solvent signals as internal standards. High resolution mass spectra (HRMS) were obtained from the *Laboratory of Mass Spectrometry* at the *Institut für Chemie*, *Technische Universität Berlin*. ESI mass spectra were recorded on an Orbitrap LTQ XL of Thermo Scientific mass spectrometer, and the raw data evaluated using the X-calibur™ computer program. Melting point samples were sealed in a glass capillary under nitrogen. Magnetic measurements including EPR spectroscopy and SQUID were performed at the *Department of Chemistry & Pharmacy, Friedrich-Alexander-University, Erlangen - Nürnberg (FAU)*.

Magnetism data: Magnetism data of microcrystalline and powdered samples (15.8–22.1 mg), loaded within polycarbonate gel capsules, were collected on a Quantum Design MPMS-3 SQUID magnetometer. To test for reproducibility, two independently synthesized samples were measured for each compound. DC susceptibility was recorded in the temperature range of 2–300 K with an applied DC field of 1 T, if not stated otherwise. Values of the magnetic susceptibility were corrected for core diamagnetism of the sample using tabulated Pascal's constants.[2] For simulation and analysis of the data, the program "JulX2", written by Dr. Eckhard Bill (MPI CEC, Mülheim/Ruhr) was used.[3]

Electron Paramagnetic Resonance (EPR) Spectroscopy: EPR spectra were recorded on a JEOL continuous wave spectrometer JES-FA200, equipped with an X-band Gunn diode oscillator bridge, a cylindric mode cavity, and a helium cryostat. The samples were measured in solution under a nitrogen atmosphere in quartz glass EPR tubes at 293, 95, and 7 K. The spectra shown were measured using the following parameters: microwave frequency = 8.959 GHz, modulation amplitude 1.0, 0.5, and 0.1 mT, microwave power 1.0 mW, modulation frequency 100 kHz, time constant of 0.1 s. Data analysis and simulation of the

data was performed using the software "eview" and "esim", written by Dr. Eckhard Bill (MPI CEC, Mülheim/Ruhr),[4,5] on the basis of a spin Hamiltonian description of the electronic ground state:

$$\hat{H} = D\left(\hat{S}_z^2 - \frac{1}{3}S(S+1) + \frac{E}{D}\left(\hat{S}_x^2 - \hat{S}_y^2\right)\right) + \mu_B \underline{g}\vec{S}$$

Here, S represents the total spin quantum number of the coupled system, D and E/D are the axial and rhombic zero-field parameters, respectively, and $\underline{g}$ is the g-matrix. Calculations are based on the S = 5/2 routines developed by Gaffney and Silverstone.[6] EPR line widths, W, are given in units of mT and 10^{-4} cm^{-1} / GHz at full-width-half-maximum (FWHM).

Cyclic Voltammetry Measurement: Cyclic Voltammetry (CV) measurements were carried out at 295 K by using a Biologic SP-150 potentiostat and a three-electrode setup inside a glove-box. Pt-wire was used as an auxiliary electrode. A freshly polished glassy carbon disc (3 mm diameter) as a working electrode and a pseudo reference electrode Ag/Ag^+ was used. All cyclic voltammograms were referenced against the Cp_2Fe/Cp_2Fe^+ redox couple which was used as an internal standard. As an electrolyte, 0.3 M solutions of TBAPF6 in THF was used. The iR-drop was determined and compensated by using the impedance measurement technique implemented in the EC-Lab Software V10.

Single crystal X-ray structure analyses: Crystals were mounted on a glass capillary in perfluorinated oil and measured in a cold N_2 flow. The data for all compounds were collected on an Agilent Technologies SuperNova (single source) at 150 K (Cu-K_α radiation, λ = 1.5418 Å). All structures were solved by direct methods and refined on F^2 with the SHELX-97 software[7]. The positions of the H atoms were calculated and considered isotopically according to a riding model. Compound **2** crystallizes with a free THF molecule in the asymmetric unit. Compound **3** crystallizes with a free hexane molecule in the asymmetric unit. Compound **5** crystallizes with two overlapped benzene molecule in the asymmetric unit. **CCDC**: 2175816 (**2**), 2175817 (**3**), 2175818 (**4**), and 2175819 (**5**) contain the supplementary crystallographic data for this paper. These data can be obtained free of charge from The Cambridge Crystallographic Data Centre via www.ccdc.cam.ac.uk/structures/

2. Experimental Section

2.1 Syntheses and Spectroscopic Data of Reported Compounds

General procedure for the synthesis of [SiNSi]MnX$_2$ (X = Cl, Br) complexes: A mixture of bis-silylene **[SiNSi]** (1.1 equiv.) and MnX$_2$ (1 equiv.) was taken in a 100 ml Schlenk flask. To this 30 ml THF was added with the cannula. After stirring 16 h at room temperature a clear yellow solution was obtained. All volatiles were removed under vacuum. The residue was washed with diethyl ether (10 mL) and filtered with cannula and dried under vacuum resulted in an off-white solid.

2.1.1 Compound [SiNSi]MnCl$_2$ (1):

Prepared via above general procedure at 1.0 mmol scale, 92% yield. The obtained magnetic moment by Evans method, IR and Elemental analysis data was matched the reported data. Here, complex **1** was further characterized by EPR spectroscopy and SQUID measurement.

Melting Point T/°C: 220 (decomp.)

SQUID: $\mu_{eff,\ plateau}$ = 6.10 B.M. at 300 K (average value obtained from 2 independent batches).

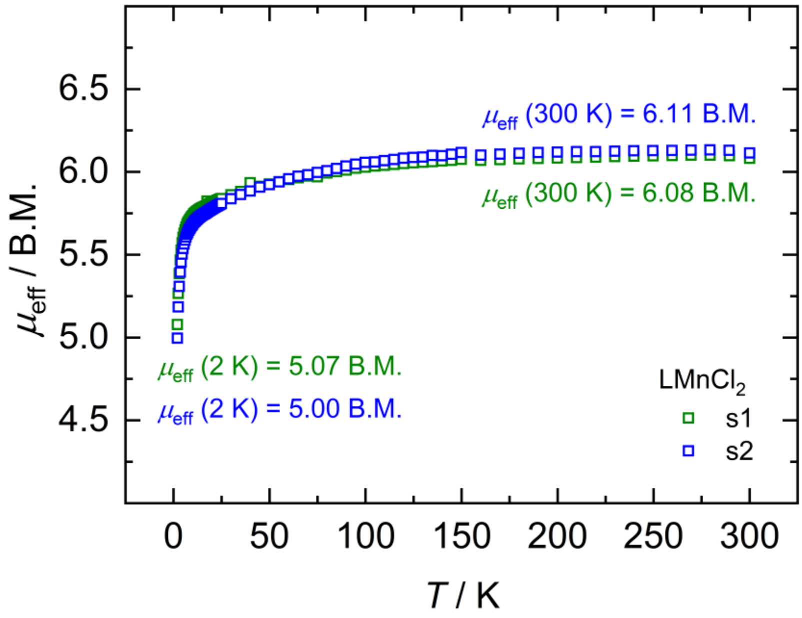

Figure S1: Temperature-dependent SQUID magnetization data (2–300 K at 1 T) for two independently synthesized batches of **1**, sample 1 (green squares) and sample 2 (blue squares), plotted as a function of the effective magnetic moment (μ_{eff}) *vs*. temperature (T). .

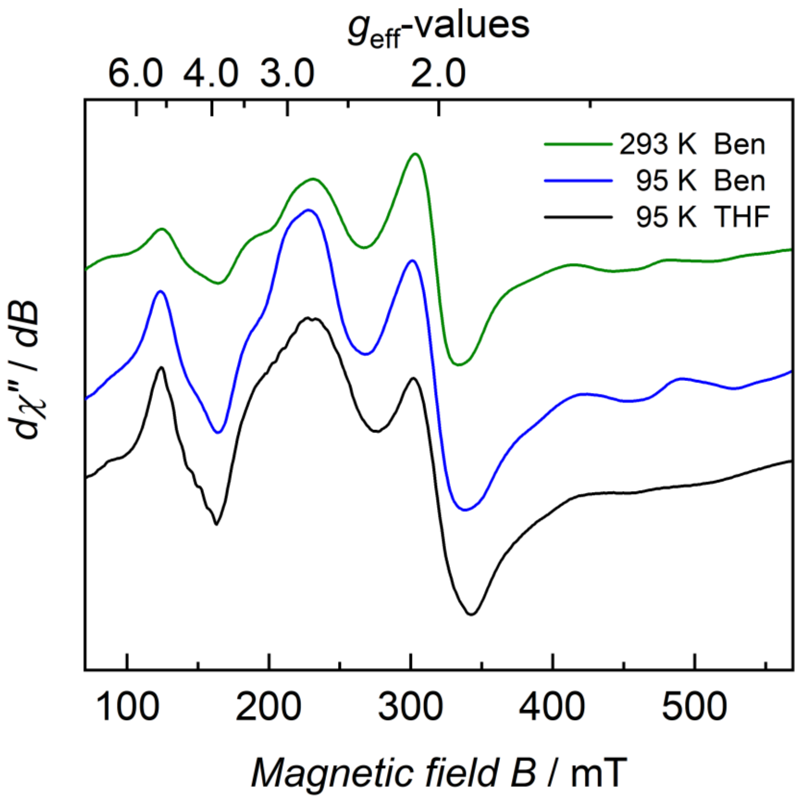

Figure S2: CW X-band EPR spectra of **1** recorded as a 1mM solution in THF at 95 K (black trace), in benzene at 95 K (blue trace), and in benzene at 293 K (green trace). Experimental conditions: microwave frequency ν = 8.959 GHz, modulation amplitude = 1.0 mT (benzene); 0.5 mT (THF), microwave power = 1.0 mW, modulation frequency = 100 kHz, time constant = 0.1 s.

2.1.2 Compound [SiNSi]MnBr$_2$ (2): at 1.0 mmol scale, 90% yield. Colorless rectangular shaped crystals were obtained by keeping concentrated THF solution of **2** at –20 °C overnight.

Melting Point (T/°C): 240 (decomp.).

Evans (THF-d_8, tetramethylsilylsilane capillary, 200 MHz, 298 K): 5.51 B.M.

SQUID: $\mu_{eff,\ plateau}$ = 5.99 B.M. at 300 K (average value obtained from 2 independent batches).

ESI-MS m/z (%): calculated for $[M]^+$ $[C_{39}H_{59}N_7Si_2MnBr_2]^+$ = 896.2092, found = 896.2083.

Elemental analysis: Calcd. for $C_{39}H_{59}N_7Si_2MnBr_2$: C, 52.11; H, 6.84; N, 10.91. Found: C, 52.04; H, 6.79; N, 10.86.

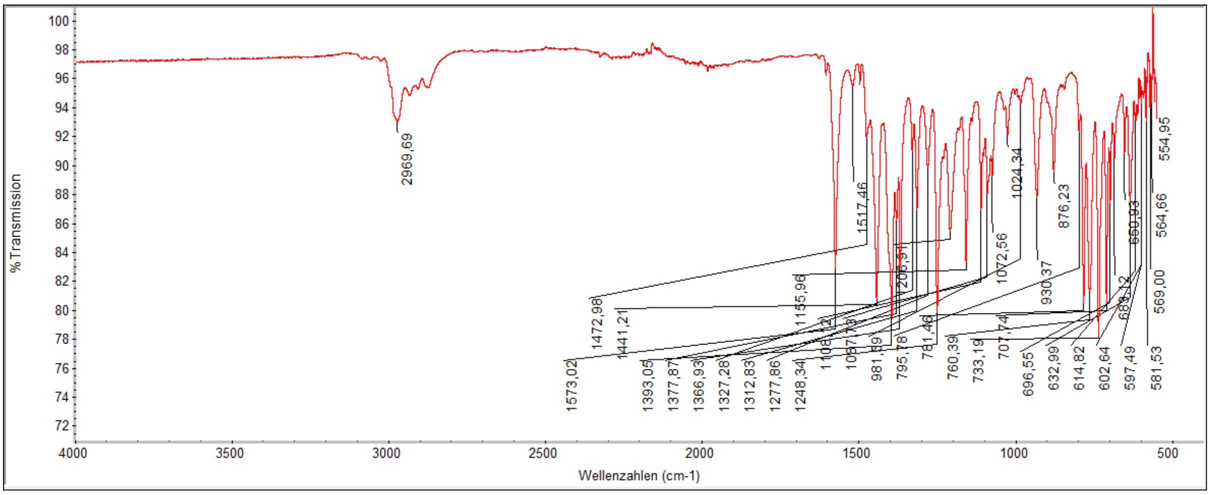

Figure S3: FT-IR spectrum of **2**.

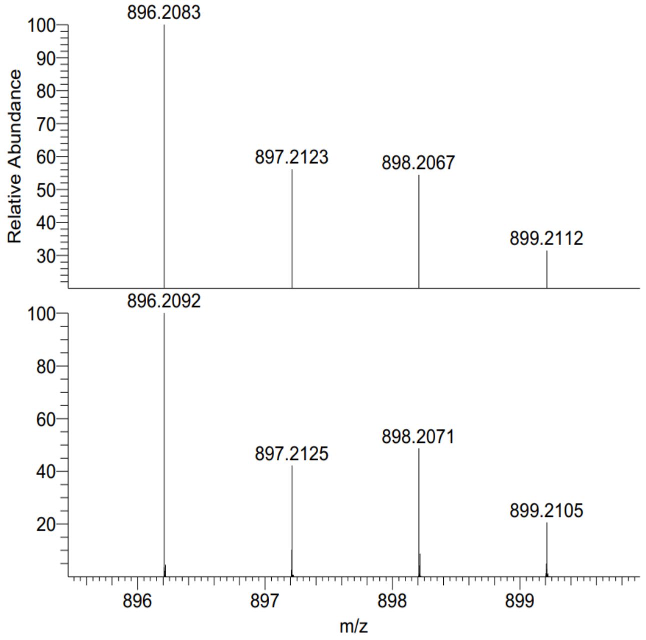

Figure S4: ESI-MS spectrum of **2** (Top: observed spectrum, bottom: calculated spectrum).

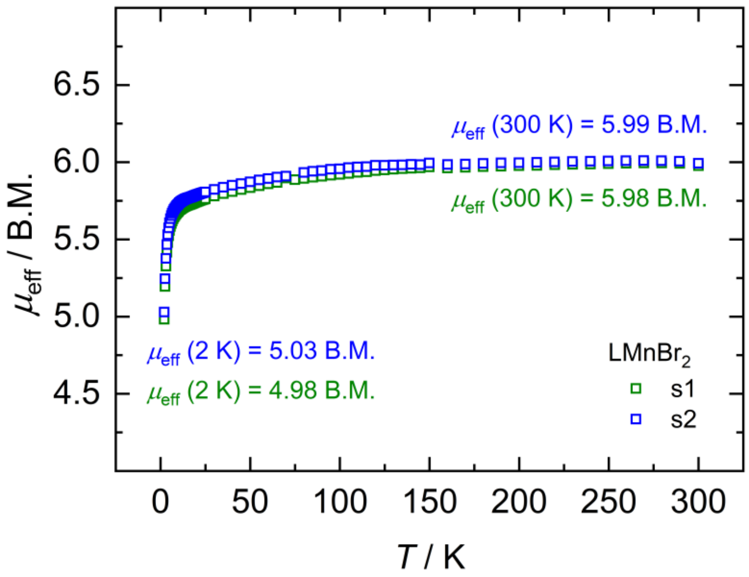

Figure S5: Temperature-dependent SQUID magnetization data (2–300 K at 1 T) for two independently synthesized batches of **2**, sample 1 (green squares) and sample 2 (blue squares), plotted as a function of the effective magnetic moment (μ_{eff}) *vs*. temperature (*T*)..

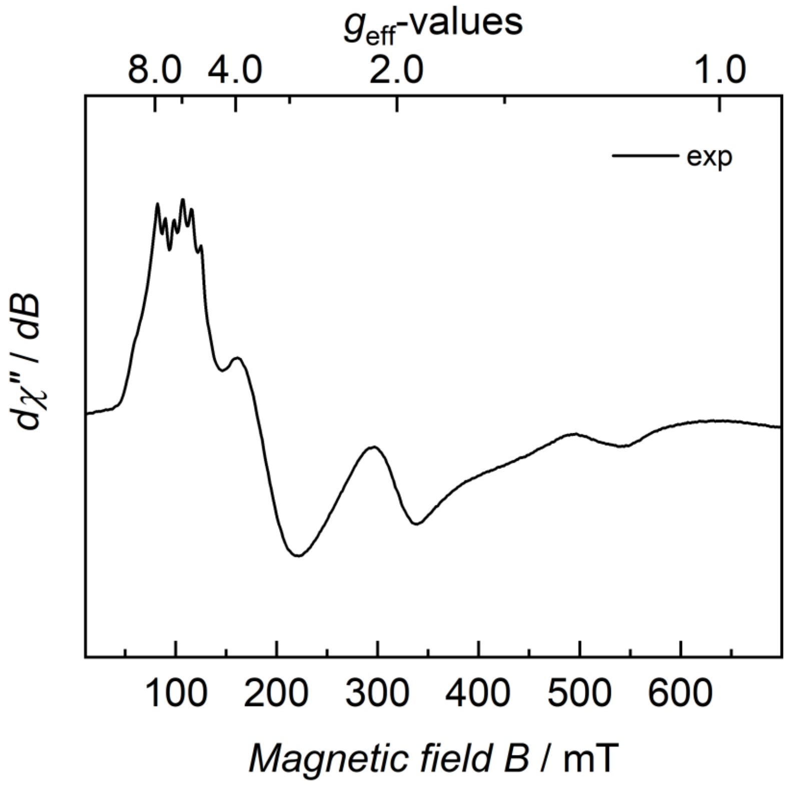

Figure S6: CW X-band EPR spectrum of **2** recorded as a 1 mM solution in THF (black trace) at 95 K. Experimental conditions: microwave frequency ν = 8.959 GHz, modulation amplitude = 1.0 mT, microwave power = 1.0 mW, modulation frequency = 100 kHz, time constant = 0.1 s.

2.1.3 Compound [Si^{II}NSi^{IV}]Mn(H)(dmpe) (3): A 100 ml Schlenk tube was charged with 500 mg (1.0 equiv.) of Mn(II)-dihalide complex **1** (0.619 mmol) or **2** (0.557 mmol) and 2.4 equiv. KC_8 (201 mg or 181 mg) in a glovebox. To this 15 mL of cold THF was added with stirring at –40 °C. After stirring the mixture at this temperature for 10 min, a solution of 1 equiv.1,2-Bis(dimethylphosphino)ethane (103 µL or 93 µL) in 5 mL THF solution was added. After stirring for 24 h, the reaction mixture was filtered and the volatiles were removed under vacuum. The red-brown residue was then extracted in 40 mL of hexane. The solution was concentrated and kept at –20 °C overnight. The compound was isolated as dark red crystals in 19% (170 mg, 0.192 mmol, starting from **1**) and 25% (223 mg, 0.251 mmol, starting from **2**) yield. However, subsequent filtration and drying of the crystals for 2 h under vacuum, resulted in a sticky solid. Single crystals suitable for an X-ray diffraction analysis were obtained by keeping a concentrated hexane solution at –20 °C for 2 days.

Melting Point (T/°C): 145 (decomp.)

Evans (THF-d_8, 200 MHz, 298 K): 3.70 B.M.

ESI-MS m/z (%): calculated for $[M]^+$ $[C_{45}H_{75}N_7Si_2MnP_2]^+$ = 886.4473, found = 886.4512.

Elemental analysis: Calcd. for $C_{45}H_{75}N_7Si_2MnP_2$: C, 60.85; H, 8.63; N, 11.04. Found: C, 60.81; H, 8.58; N, 10.98

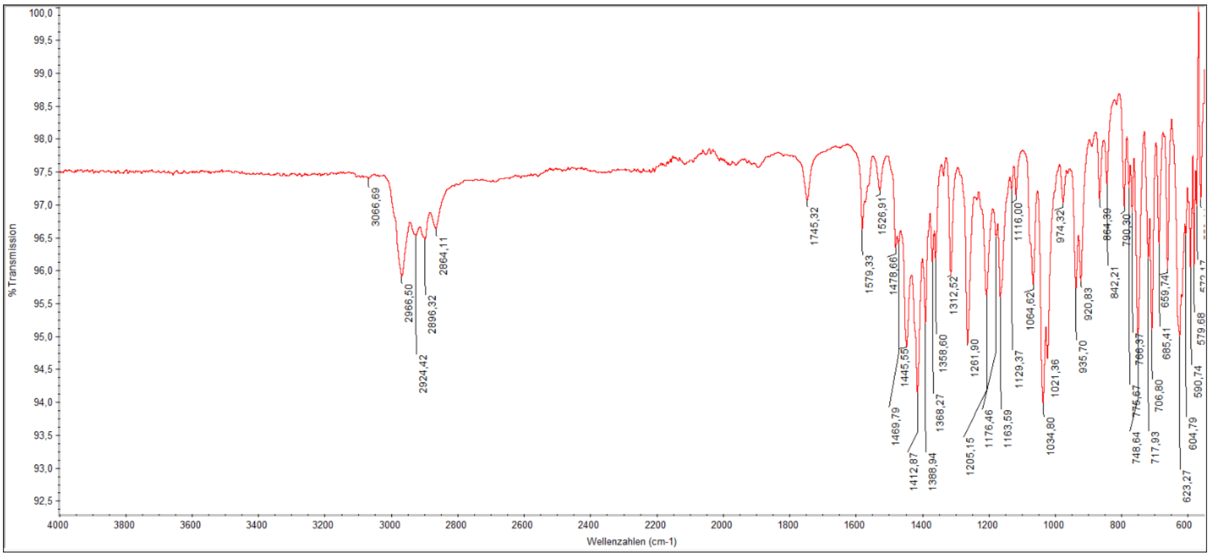

Figure S7: FT-IR spectrum of compound **3**

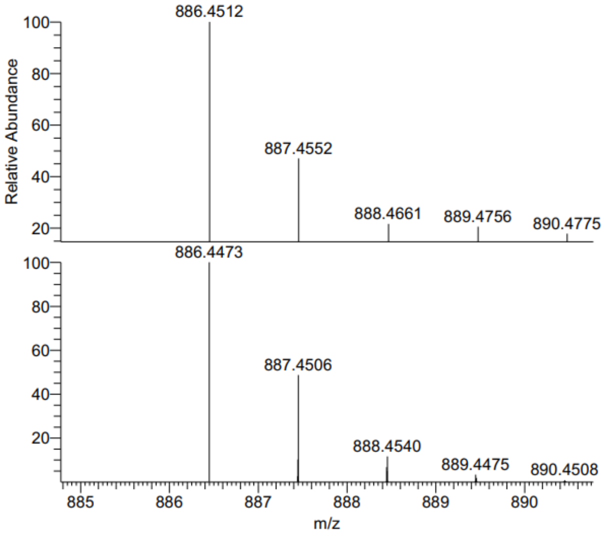

Figure S8: ESI-MS spectrum of compound **3** (Top: observed spectrum, bottom: calculated spectrum).

2.1.4 Compound [SiNSi]Mn(dmpe) (4): A 100 ml schlenk tube was charged with one equiv. of compound **1** (800 mg, 0.990 mmol) or **2** (800 mg, 0.892 mmol). To this 25 mL of THF was added by cannula, followed by an addition of one equivalent 1,2-bis(dimethylphosphino)ethane (166 µL or 149 µL) in a 5 mL THF solution. This mixture, was stirred for 2 hours. The solution was cooled to 0°C, which was then transferred to to a well-stirred cold suspension containing 2.4 equiv. KC_8 (321 mg or 289 mg) in THF at –40 °C. After stirring the mixture for 16 h, it was filtered using cannula filter. The filtrate was dried under vacuum for 2 hours. The residue was then extracted in hexane (2×50mL) as dark blue-black solution. The filtrate was concentrated to 10-15 mL and kept in freezer overnight giving a crop of diamond shape black crystals. Subsequent filtration and evaporation under vacuum afforded black shiny crystalline solid in 39% (340 mg, 0.383 mmol, starting from **1**) and 50% (396 mg, 0.446 mmol, starting from **2**) yield. Single crystals suitable for X-ray diffraction analysis were obtained from a concentrated hexane solution at room temperature overnight at room temperature.

Melting Point (T/°C): 135 (decomp.)

Evans (C_6D_6, tetramethylsilylsilane capillary, 200 MHz, 298 K): 2.65 B.M.

SQUID: $\mu_{eff,\ plateau}$ = 1.95 B.M. at 300 K; 1.62 B.M. at 2 K. (values are obtained from the average of two independent batches)

ESI-MS m/z (%): calculated for $[M+2H]^+$ $[C_{45}H_{77}N_7Si_2MnP_2]^+$ = 888.4629, found = 888.4631.

Elemental analysis: Calcd. for $C_{45}H_{75}N_7Si_2MnP_2$: C, 60.78; H, 8.73; N, 11.03. Found: C, 60.72; H, 8.69; N, 10.96.

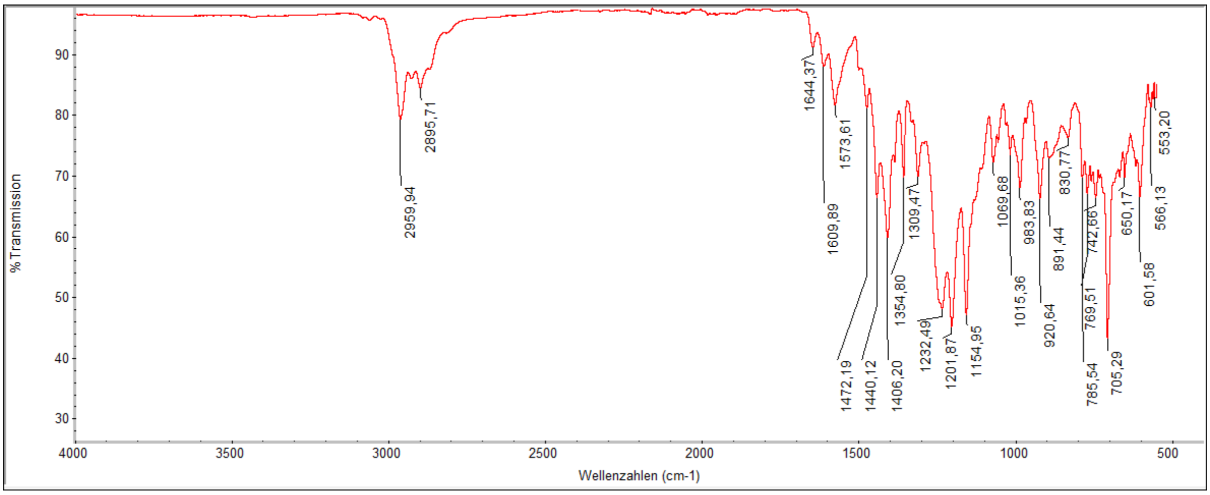

Figure S9: FT-IR spectrum of compound **4**

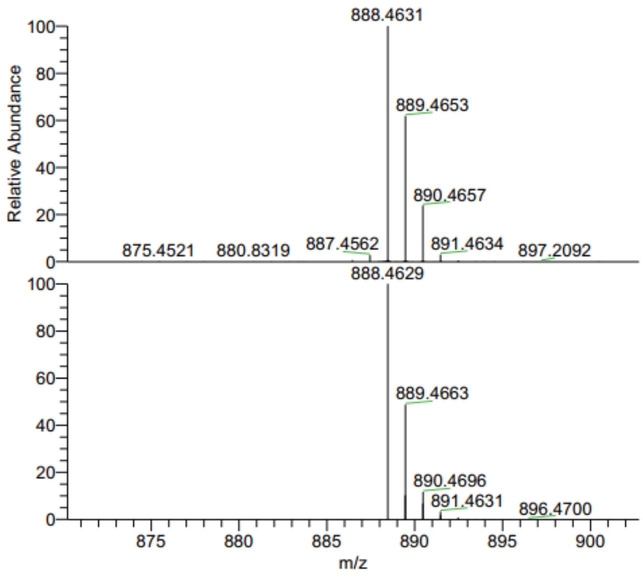

Figure S10: ESI-MS spectrum of compound **4** (Top: observed spectrum, bottom: calculated spectrum).

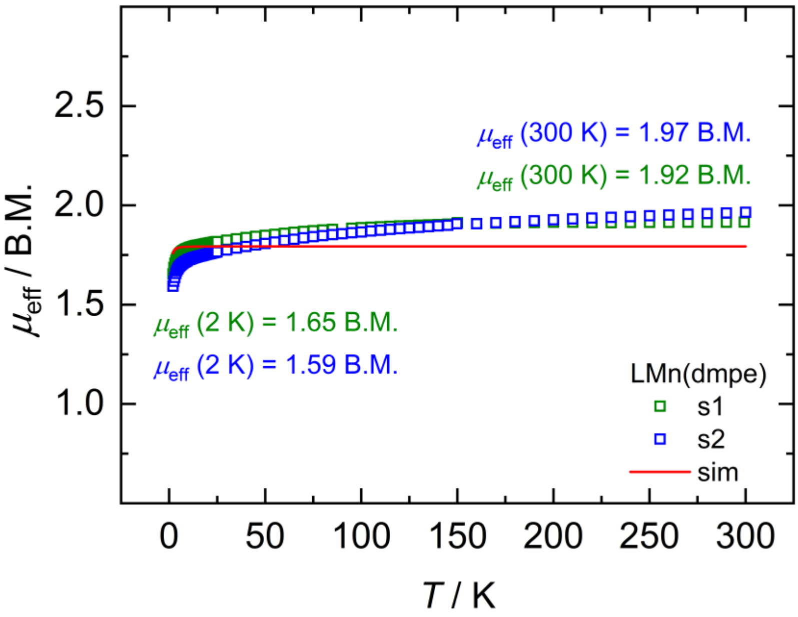

Figure S11: Temperature-dependent SQUID magnetization data (2–300 K at 1 T) for two independently synthesized batches of **4**, sample 1 (green squares) and sample 2 (blue squares), plotted as a function of the effective magnetic moment (μ_{eff}) *vs*. temperature (T) and corrected for temperature independent paramagnetism, TIP = $420\cdot10^{-6}$ emu. A simulation for S = 1/2 and g_{avg} = 2.07 is represented as a solid red trace as a comparison.

.

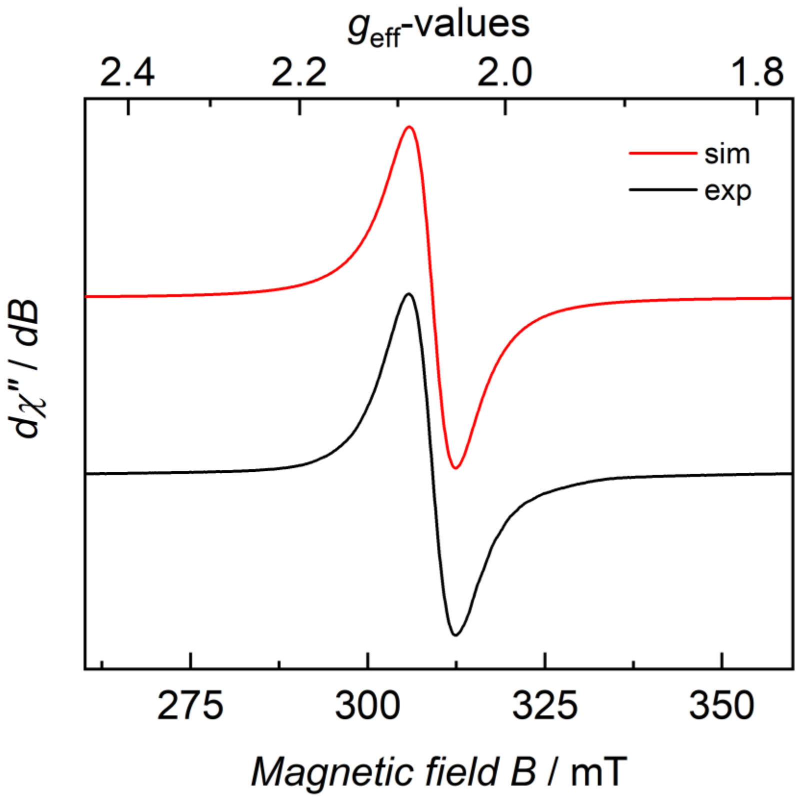

Figure S12: CW X-band EPR spectrum of **4** recorded as a 1 mM solution in THF at 95 K (black trace) and its simulation (red trace). Experimental conditions: microwave frequency ν = 8.959 GHz, modulation amplitude = 1.0 mT, microwave power = 1.0 mW, modulation frequency = 100 kHz, time constant = 0.1 s. Simulation parameters: effective *g*-value g_{iso} = 2.07, linewidths W_{iso} = 7.06 10^{-4} cm^{-1} / GHz, pseudo-Voigt lines used with ratios (Lorentz = 0, Gauss = 1) V_{iso} = 0.00.

2.1.5 Compound [Si,Si']Mn(CO)$_3$ (5): A solution of compound **4** (70 mg, 0.079 mmol) in 10 mL of toluene was set up under a CO atmosphere after three freeze−pump−thaw cycles. The reaction mixture was stirred for 6 hours at room temperature. The dark black-violet color of the reaction faded to wine red color over the course of the reaction. The volatiles were removed under vacuum. The residue was extracted in 30 mL hexane and was concentrated to 5 mL. Keeping it at –20 °C overnight afforded the first crop of desired product (30 mg). Further concentration, crystallization, from the remaining hexane solution afforded the second crop of product (25 mg) for a total yield of 55 mg of the product (85%, 0.067 mmol). Single crystals suitable for an X-ray diffraction analysis were obtained from a concentrated diethyl ether/benzene solution at room temperature after 2 days.

Melting Point (T/°C): 160 (decomp.)

Evans (THF-d_8, 200 MHz, 298 K): 1.80 B.M.

SQUID: μ_{eff} = 1.18 B.M. at 300 K

ESI-MS m/z (%): calculated for $[M+2H]^+$ $[C_{42}H_{59}MnN_7O_3Si_2]^+$ = 822.3749, found = 822.3753.

Elemental analysis: Calcd. for $C_{42}H_{57}MnN_7O_3Si_2$: C, 61.29; H, 7.47; N, 11.91. Found: C, 61.22; H, 7.49; N, 11.88.

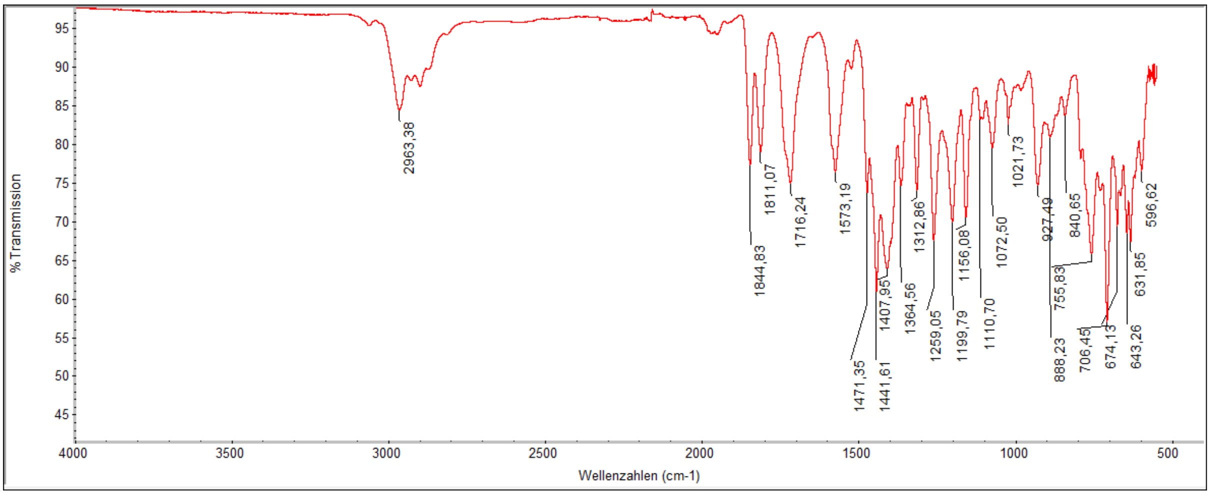

Figure S13: FT-IR spectrum of compound **5**.

(a)

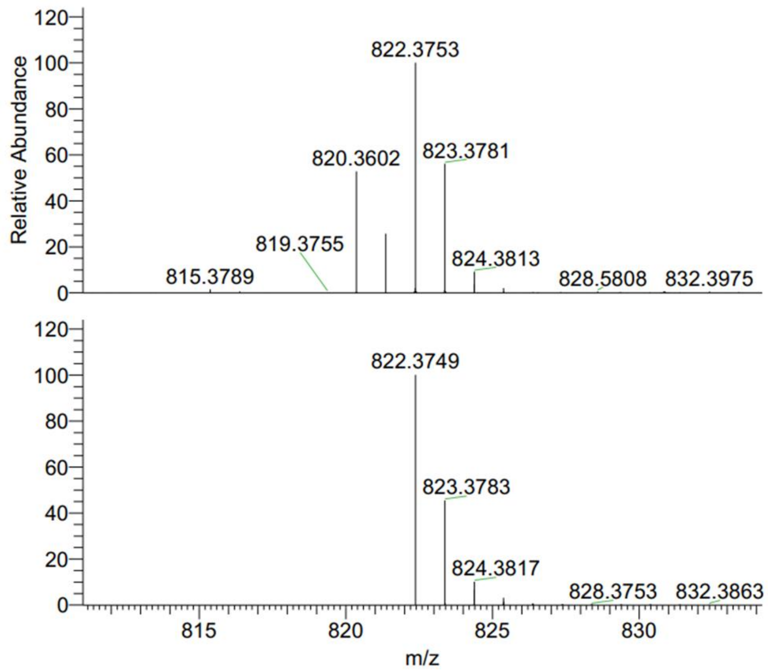

(b)

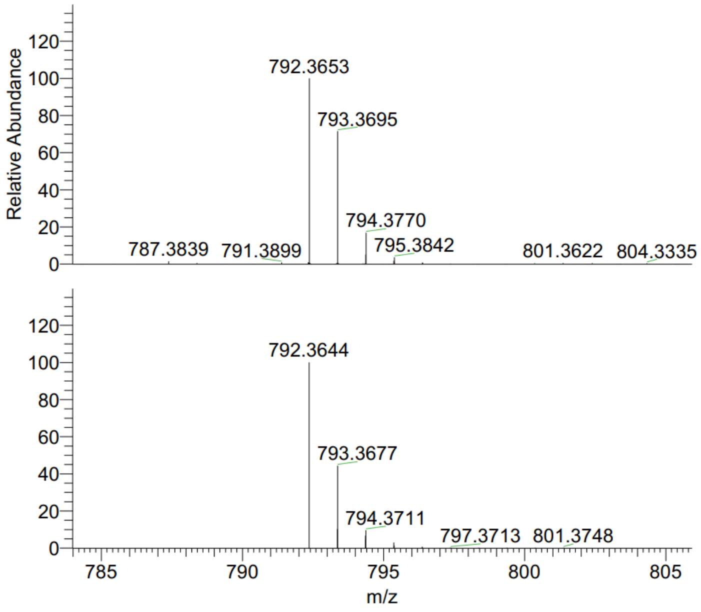

Figure S14: ESI-MS spectrum of compound **5** (Top: observed spectrum, bottom: calculated spectrum). (a) for $[M+2H]^{+}$ (b) for $[M–CO]^{+}$

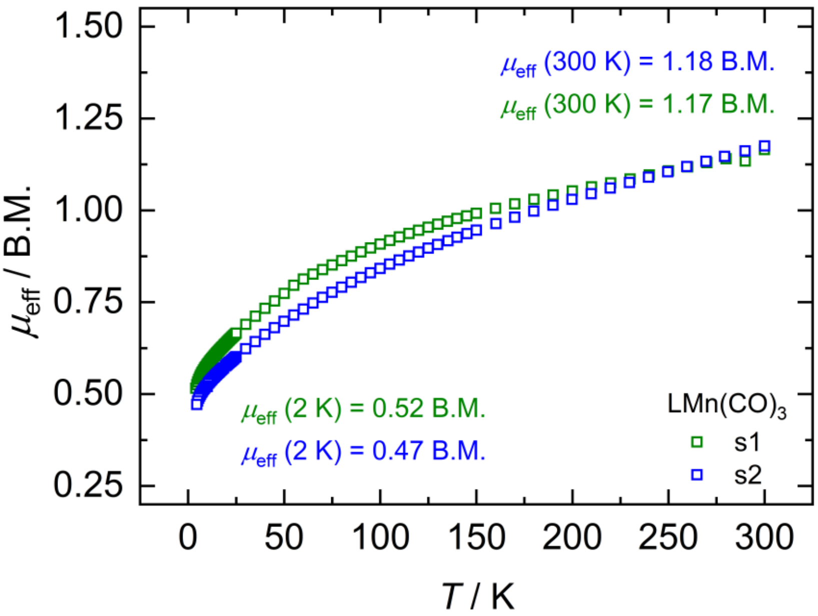

Figure S15: Temperature-dependent SQUID magnetization data (2–300 K at 1 T) for two independently synthesized batches of **5**, sample 1 (green squares) and sample 2 (blue squares), plotted as a function of the effective magnetic moment (μ_{eff}) *vs*. temperature (*T*).

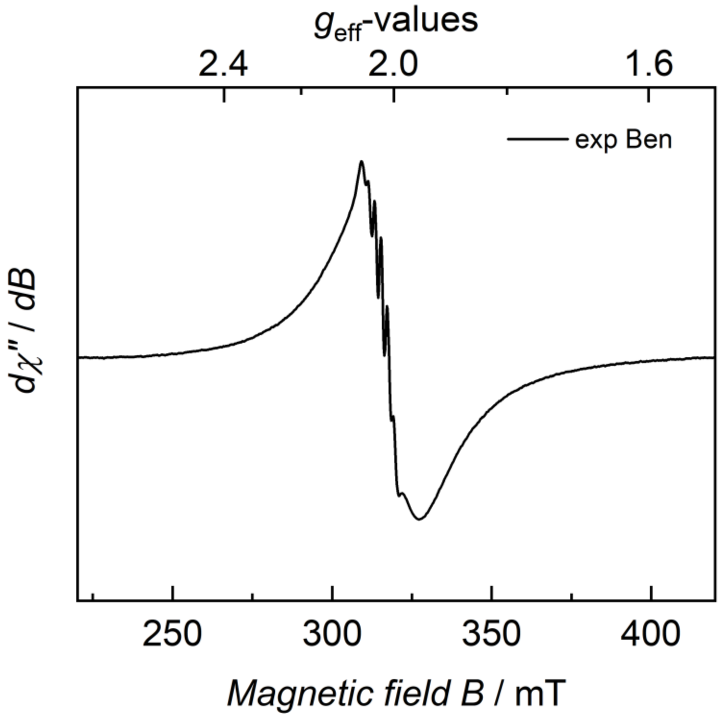

Figure S16: CW X-band EPR spectra of **5** recorded as a 5 mM solution in benzene (black trace), and in toluene (blue trace) at 293 K. Experimental conditions: microwave frequency

ν = 8.959 GHz, modulation amplitude = 1.0 mT, microwave power = 1.0 mW, modulation frequency = 100 kHz, time constant = 0.1 s.

2.1.6 Compound [SiNSi]Mn(XylylNC)$_2$(dmpe) (6): Compound **4** (50mg, 0.056 mmol) and 2.0 equiv. 2,6-dimethylphenyl isocyanide (14.7 mg, 0.112 mmol) were weighed inside the glove-box in a 50 mL schlenk tube. To this 10 mL of toluene was added at room temperature. The reaction mixture was stirred overnight, affording a dark green solution. The volatiles were removed under vacuum. The residue was washed with (2×3ml) cold hexane and dried to give a yield of 80% (52.0 mg, 0.045 mmol).

Melting Point (T/°C): 150 (decomp.)

Evans (THF-d_8, 200 MHz, 298 K): 1.86 B.M.

SQUID: $\mu_{eff,\ plateau}$ = 1.83 B.M. at 300 K

APCI-MS m/z (%): calculated for $[M]^+$ $[C_{63}H_{93}MnN_9P_2Si_2]^+$ = 1148.5943, found = 1148.5951.

Elemental analysis: Calcd. for $C_{63}H_{93}MnN_9P_2Si_2$: C, 65.71; H, 8.32; N, 10.95. Found: C, 65.68; H, 8.29; N, 10.91.

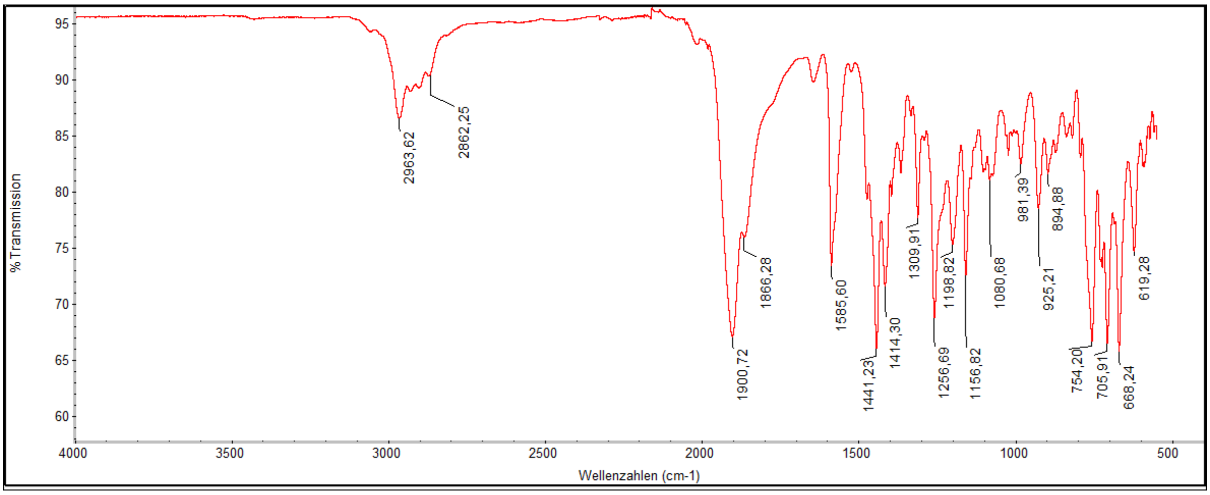

Figure S17: FT-IR spectrum of compound **6**.

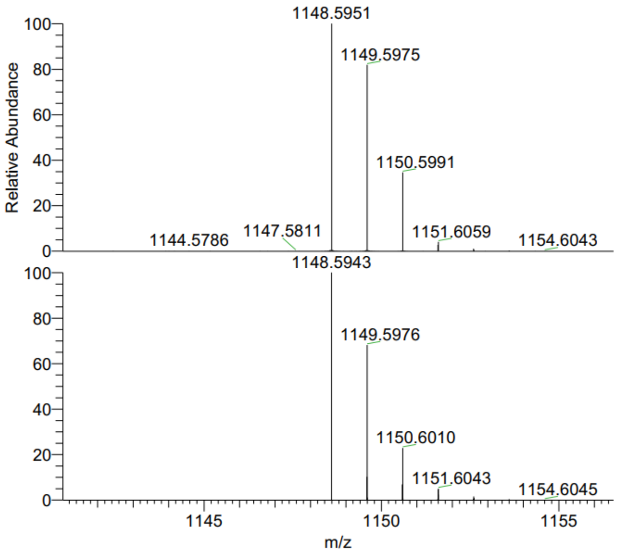

Figure S18: APCI-MS spectrum of compound **6** (Top: observed spectrum, bottom: calculated spectrum).

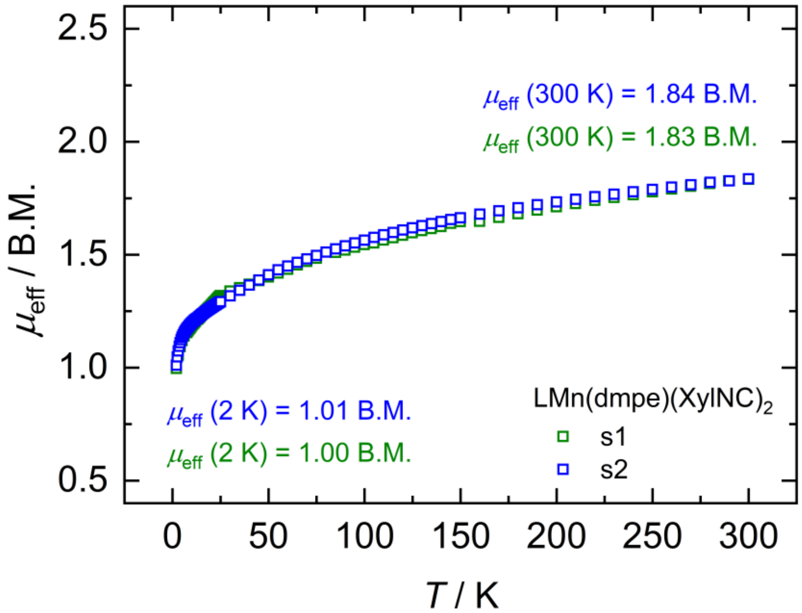

Figure S19: Temperature-dependent SQUID magnetization data (2–300 K at 1 T) for two independently synthesized batches of **6**, sample 1 (green squares) and sample 2 (blue squares), plotted as a function of the effective magnetic moment (μ_{eff}) *vs*. temperature (*T*)..

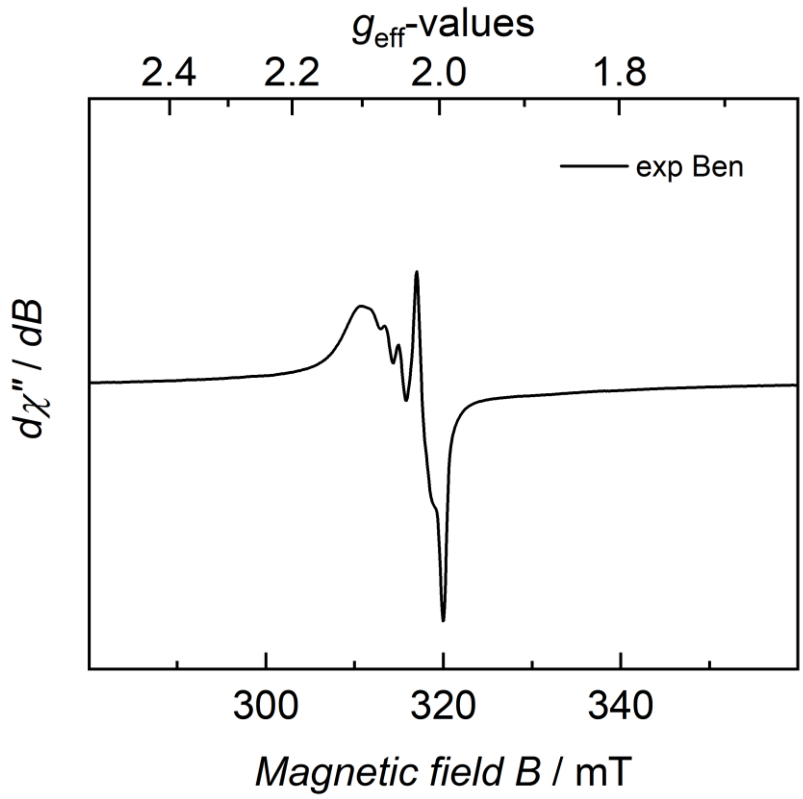

Figure S20: CW X-band EPR spectrum of **6** recorded as a 5 mM solution in benzene at 293 K (black trace). Experimental conditions: microwave frequency ν = 8.959 GHz, modulation amplitude = 1.0 mT, microwave power = 1.0 mW, modulation frequency = 100 kHz, time constant = 0.1 s.

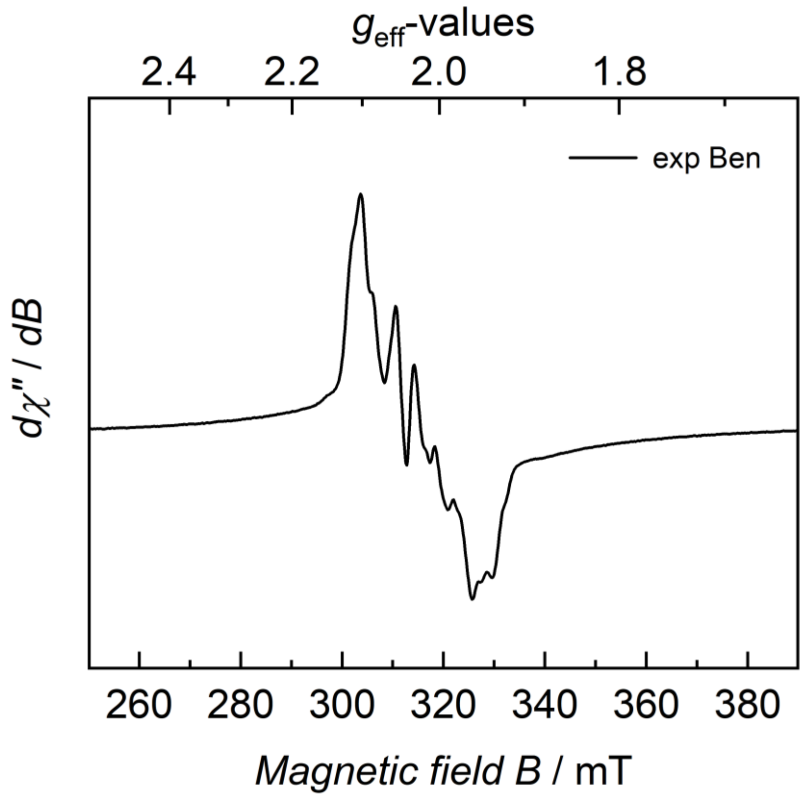

Figure S21: CW X-band EPR spectrum of **6** recorded as a 5 mM solution in benzene at 95 K (black trace). Experimental conditions: microwave frequency ν = 8.959 GHz, modulation amplitude = 1.0 mT, microwave power = 1.0 mW, modulation frequency = 100 kHz, time constant = 0.1 s.

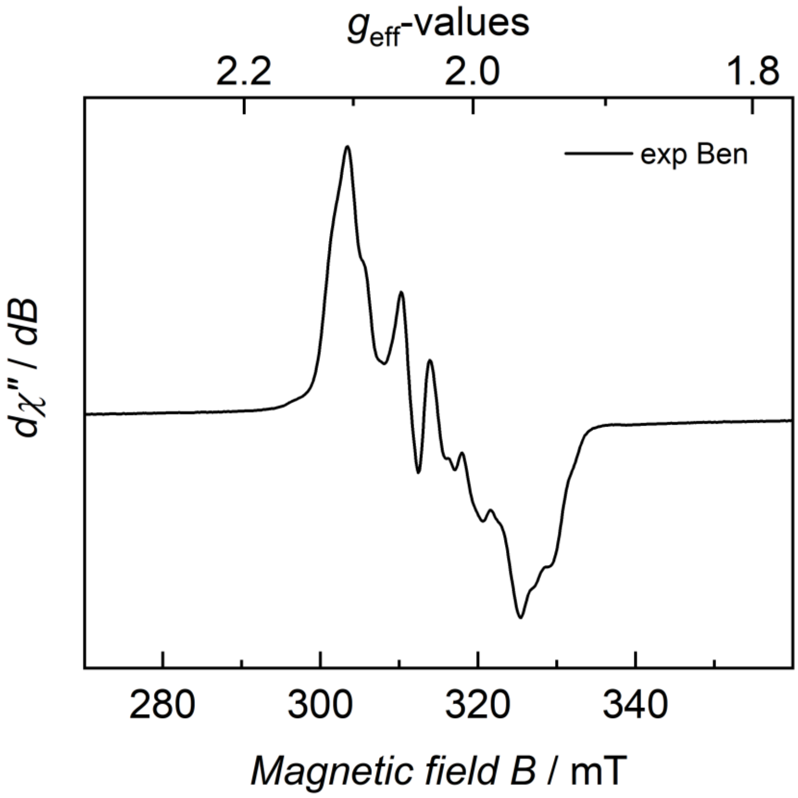

Figure S22: CW X-band EPR spectrum of **6** recorded as a 5 mM solution in benzene at 9 K (black trace). Experimental conditions: microwave frequency ν = 8.959 GHz, modulation amplitude = 1.0 mT, microwave power = 1.0 mW, modulation frequency = 100 kHz, time constant = 0.1 s.

3. Experimental conditions for Catalytic hydroboration of N-heteroarenes with HBpin using [SiNSi]Mn(dmpe) (4) as a precatalyst

3.1 Optimization of reaction conditions

In a glove box, suitable catalyst (**1-6**) (5.0 mol%, 5.0 µmol) and reducing agent (H–BR$_2$, 0.2 mmol) was added to a solution containing quinoline (0.1 mmol) in 0.45 mL C_6D_6 in a J. Young type NMR tube. After taken out of the glovebox and heated at specified temperature for specified time. Reaction progress was monitored by ^{1}H-NMR spectroscopy.

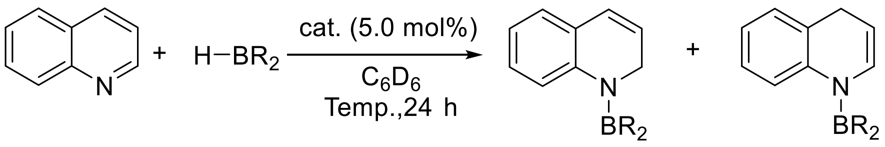

Entry	Catalyst	H-BR$_2$	Temp.(°C)	Conv.(%)
1	1	HBpin	50	<5
**2**	**4**	**HBpin**	**50**	**97(85:15)**
3	2	HBpin	50	<5
4	5	HBpin	50	17(85:15)
5	6	HBpin	50	30(90:10)
6	**4**	HBcat	50	40(20:80)
7	**4**	9-BBN	50	55(20:80)
8	**4**	HBpin	25	<5

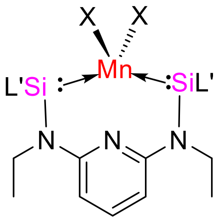

X =Cl, **1**
X= Br, **2**

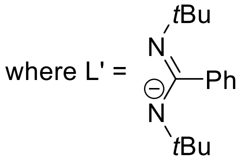

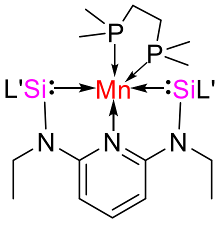

4

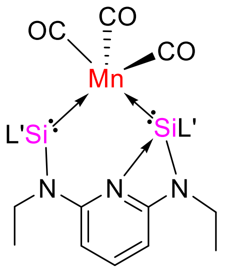

5

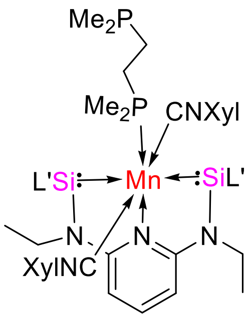

6

Reaction conditions: substrate (0.1 mmol), cat. (5 mol%), C_6D_6 (0.45 mL) at 50 °C; yield was determined by ^{1}H NMR using mesitylene as internal standard

Table S1: Optimization of reaction parameters for hydroboration of N-heteroarenes.

3.2 Preparative scale reaction

In a N_2 filled glovebox, a 25 mL schlenk tube was charged with quinoline (118 µL, 1.0 mmol), HBpin (2.0 equiv, 2.0 mmol), and 5 mol% of compound **4** (4.4 mg, 0.05 mmol). To this 1.0 mL of C_6D_6 was added. The flask was sealed and taken outside. The reaction mixture was heated at 50 °C for 24 hours. After cooling the flask to room temperature, the volatiles were removed. The residue was dissolved in hexane and product was obtained upon recrystallization.

Major: ^{1}H NMR (500 MHz, C_6D_6): δ(ppm) = 7.82 (d, *J* = 8.1 Hz, 1H), 7.12 – 7.08 (m, 1H), 6.86 – 6.78 (m, 2H), 6.26 (d, *J* = 9.5 Hz, 1H), 5.59 – 5.55 (m, 1H), 4.16 (dd, *J* = 4.1, 1.4 Hz, 2H), 1.04 (s, 12H). ^{13}C NMR (126 MHz, C_6D_6) δ(ppm) = 141.9, 127.8, 126.7, 126.6, 124.2, 121.6, 120.9, 82.5, 43.3, 24.3. ^{11}B NMR (160 MHz, C_6D_6): δ(ppm) = 23.9.

Minor: ^{1}H NMR (500 MHz, C_6D_6): δ(ppm) = 8.16 (d, *J* = 8.3 Hz, 1H), 6.95 – 6.90 (m, 2H), 6.89 – 6.86 (m, 2H), 4.86 – 4.81 (m, 1H), 3.32 (d, *J* = 1.7 Hz, 2H), 1.02 (s, 12H). ^{11}B NMR (160 MHz, C_6D_6): δ(ppm) = 24.7.

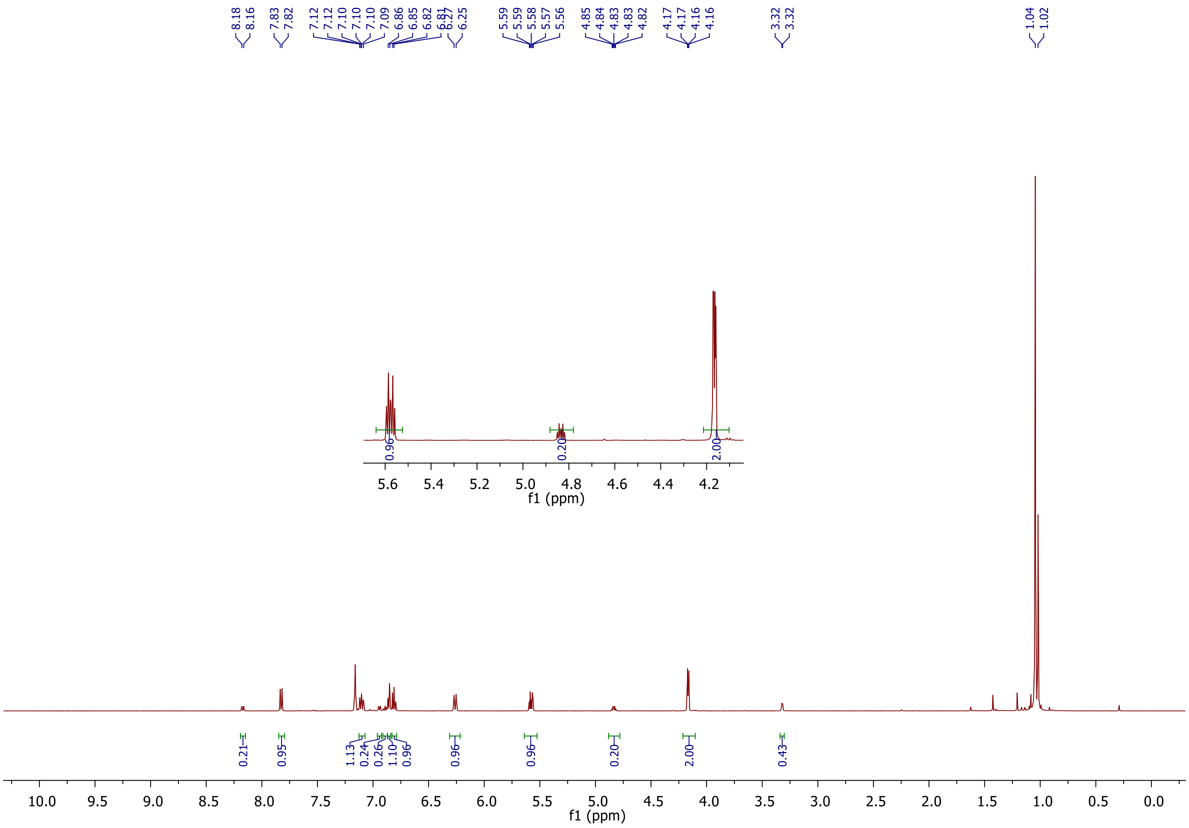

Figure S23: ^{1}H NMR (500 MHz, C_6D_6) spectrum of **8a** isolated from a preparative scale reaction.

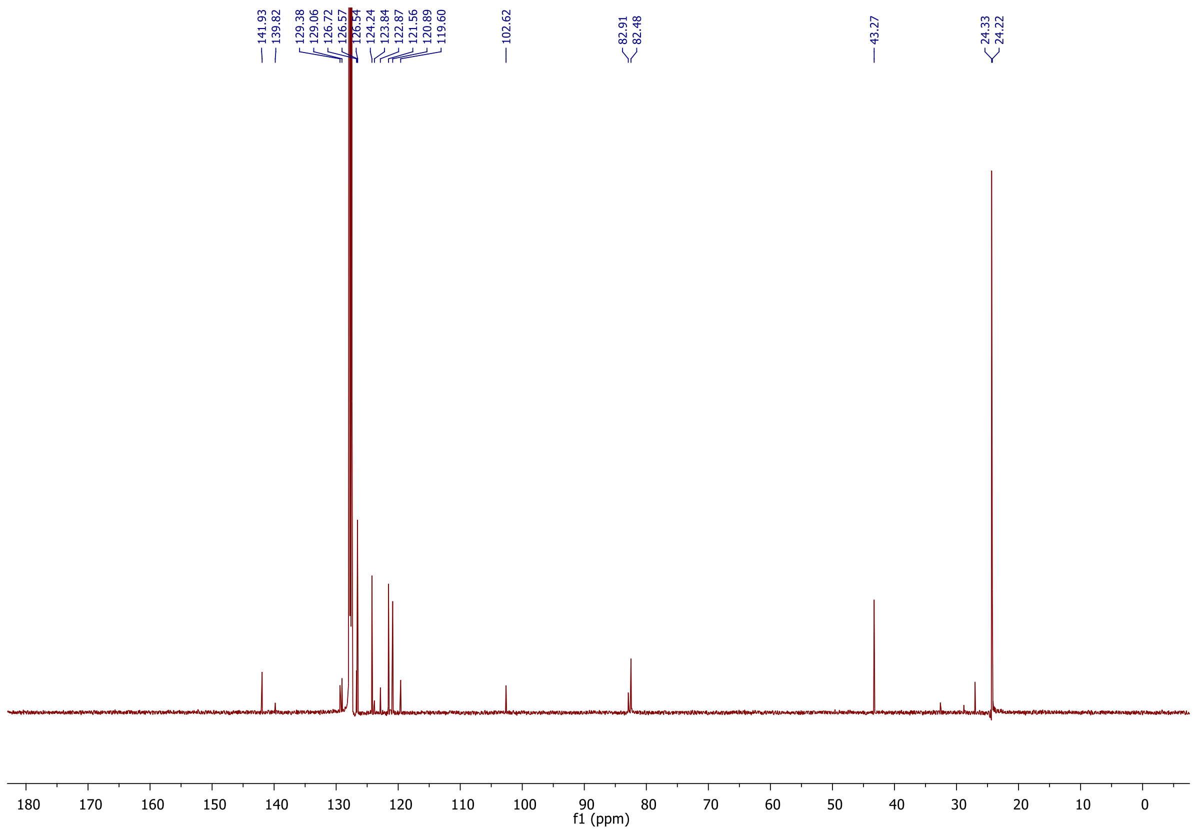

Figure S24: ^{13}C NMR (126 MHz, C_6D_6) spectrum of **8a** isolated from a preparative scale reaction.

3.3 Mercury Test Experiment

A 25 mL Schlenk tube was charged with quinoline (26.4 µL, 0.223 mmol), mesitylene (26.4 µL, 0.091 mmol, internal standard) and catalyst complex **4** [SiNSi]Mn(dmpe) (9.9 mg, 11.1 µmol) and 1 mL C_6D_6 was added into the Schlenk tube. Then mercury (250 mg, 1.15 mmol) was added into the mixture. After stirring the reaction mixture at 50 °C for 12 h, conversion was determined by ^1H-NMR spectroscopy. The hydroboration of quinoline was unaffected with >97% conversion in the presence of Hg, indicating a homogeneous process.

3.4 General procedure for the Catalytic hydroboration of N-heteroarenes with HBpin using [SiNSi]Mn(dmpe) (4) as a Precatalyst

In a glove box, 5 mol% [SiNSi]Mn(dmpe) (4.4 mg, 5.0 µmol) was added to a solution containing N-heteroarene (0.1 mmol) in 0.5 mL C_6D_6 in a J. Young type NMR tube. Then, pinacolborane HBpin (0.2-0.4 mmol) was added to the resulting mixture. After taken out of the glovebox and heated at 50 °C for specified time, it was measured by NMR spectroscopy. The NMR yields were calculated using mesitylene as an internal standard.

3.5 Spectroscopic Data of the Catalytic Hydroboration of N-heteroarenes with HBpin using [SiNSi]Mn(dmpe) (4) as a Precatalyst

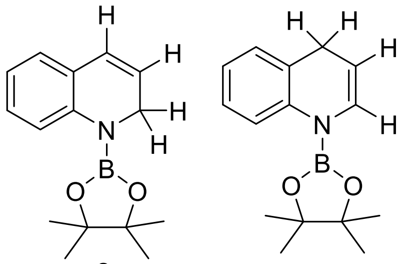

8a **8a'**

1-(4,4,5,5-Tetramethyl-1,3,2-dioxaborolan-2-yl)-1,2-dihydroquinoline (8a) and 1-(4,4,5,5-Tetramethyl-1,3,2-dioxaborolan-2-yl)-1,4-dihydroquinoline (8a'). **8a**: ^{1}H NMR (500 MHz, C_6D_6): δ(ppm) = 7.82 (d, *J* = 8.1 Hz, 1H), 7.12 – 7.08 (m, 1H), 6.86 – 6.78 (m, 2H), 6.26 (d, *J* = 9.5 Hz, 1H), 5.59 – 5.55 (m, 1H), 4.16 (dd, *J* = 4.1, 1.4 Hz, 2H), 1.04 (s, 12H). ^{11}B NMR (160 MHz, C_6D_6): δ(ppm) = 23.9.

8a': ^{1}H NMR (500 MHz, C_6D_6): δ(ppm) = 8.16 (d, *J* = 8.3 Hz, 1H), 6.93 (dt, *J* = 8.1, 1.8 Hz, 1H), 6.89 (td, *J* = 7.3, 1.2 Hz, 1H), 4.86 – 4.81 (m, 1H), 3.32 (d, *J* = 1.7 Hz, 2H), 1.02 (s, 12H). ^{11}B NMR (160 MHz, C_6D_6): δ(ppm) = 24.7.

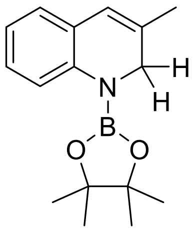

8b

3-methyl-1-(4,4,5,5-tetramethyl-1,3,2-dioxaborolan-2-yl)-1,2-dihydroquinoline (8b). ^{1}H NMR (500 MHz, C_6D_6): δ(ppm) = 7.86 (d, *J* = 8.1 Hz, 1H), 7.10 (t, *J* = 7.5 Hz, 1H), 6.89 – 6.83 (m, 1H), 6.01 (s, 1H), 4.09 (s, 2H), 1.51 (s, 3H), 1.06 (s, 12H). ^{11}B NMR (160 MHz, C_6D_6): δ(ppm) = 24.1.

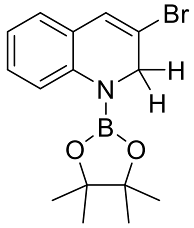

8c

3-bromo-1-(4,4,5,5-tetramethyl-1,3,2-dioxaborolan-2-yl)-1,2-dihydroquinoline (8c). ^{1}H NMR (500 MHz, C_6D_6): δ(ppm) = 7.71 (d, J = 8.1 Hz, 1H), 7.06 – 7.03 (m, 1H), 6.75 – 6.72 (m,

1H), 6.64 (d, 1H), 6.52 (s, J = 6.4 Hz, 1H), 4.41 (s, 2H), 1.00 (s, 12H). ^{11}B NMR (160 MHz, C_6D_6): δ(ppm) = 23.7.

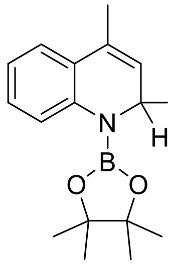

8d

4-methyl-1-(4,4,5,5-tetramethyl-1,3,2-dioxaborolan-2-yl)-1,2-dihydroquinoline (8d). ^{1}H NMR (500 MHz, C_6D_6): δ(ppm) = 7.84 (dd, *J* = 8.1, 1.0 Hz, 1H), 7.09 (dd, *J* = 7.7, 1.4 Hz, 1H), 6.88 (td, *J* = 7.5, 1.2 Hz, 1H), 5.44 (td, *J* = 4.3, 1.4 Hz, 1H), 4.14 – 4.12 (m, 2H), 1.80 (s, 3H), 1.06 (s, 12H). ^{11}B NMR (160 MHz, C_6D_6): δ(ppm) = 24.0.

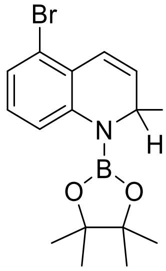

8e

5-bromo-1-(4,4,5,5-tetramethyl-1,3,2-dioxaborolan-2-yl)-1,2-dihydroquinoline (8e). ^{1}H NMR (500 MHz, C_6D_6): δ(ppm) = 7.62 (d, *J* = 8.1 Hz, 1H), 7.05 (d, *J* = 7.9 Hz, 1H), 6.85 (d, *J* = 9.6 Hz, 1H), 6.73 – 6.71 (m, 1H), 5.61 – 5.57 (m, 1H), 3.96 (d, *J* = 3.5 Hz, 2H), 1.03 (s, 12H). ^{11}B NMR (160 MHz, C_6D_6): δ(ppm) = 23.7.

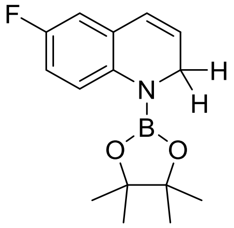

8f

6-fluoro-1-(4,4,5,5-tetramethyl-1,3,2-dioxaborolan-2-yl)-1,2-dihydroquinoline (8f). ^{1}H NMR (500 MHz, C_6D_6): δ(ppm) = 7.60 (m, 1H), 6.76 (td, *J* = 8.7, 2.9 Hz, 1H), 6.53 (dd, *J* = 8.7, 2.9 Hz, 1H), 6.02 (d, *J* = 9.6 Hz, 1H), 5.56 – 5.52 (m, 1H), 4.05 (dd, *J* = 4.0, 1.2 Hz, 2H), 1.04 (s, 12H). ^{11}B NMR (160 MHz, C_6D_6): δ(ppm) = 23.9.

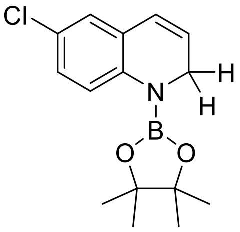

8g

6-chloro-1-(4,4,5,5-tetramethyl-1,3,2-dioxaborolan-2-yl)-1,2-dihydroquinoline (8g). ^{1}H NMR (500 MHz, C_6D_6) δ(ppm) = 7.59 (d, *J* = 8.5 Hz, 1H), 7.04 (t, *J* = 8.3 Hz, 1H), 6.80 (s, 1H), 5.97 (d, *J* = 9.2 Hz, 1H), 5.48 (d, *J* = 8.8 Hz, 1H), 4.04 (s, 2H), 1.01 (s, 12H). ^{11}B NMR (160 MHz, C_6D_6): δ(ppm) = 24.1.

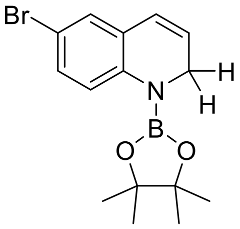

8h

6-bromo-1-(4,4,5,5-tetramethyl-1,3,2-dioxaborolan-2-yl)-1,2-dihydroquinoline (8h). ^{1}H NMR (500 MHz, C_6D_6): δ(ppm) = 7.53 (d, *J* = 8.6 Hz, 1H), 7.18 (d, *J* = 2.1 Hz, 1H), 6.95 (d, *J* = 2.1 Hz, 1H), 5.96 (d, *J* = 9.5 Hz, 1H), 5.48-5.45 (m, 1H), 4.04 (d, *J* = 2.8 Hz, 2H), 1.02 (s, 12H). ^{11}B NMR (160 MHz, C_6D_6): δ(ppm) = 23.9.

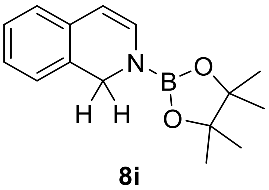

8i

2-(4,4,5,5-tetramethyl-1,3,2-dioxaborolan-2-yl)-1,2-dihydroisoquinoline (8i). ^{1}H NMR (500 MHz, C_6D_6) δ(ppm) = 7.00 (t, *J* = 7.5 Hz, 1H), 6.89 (t, *J* = 7.4 Hz, 1H), 6.83 (dd, *J* = 12.8, 7.5 Hz, 2H), 6.73 (d, *J* = 7.4 Hz, 1H), 5.64 (d, *J* = 7.5 Hz, 1H), 4.64 (s, 2H), 1.03 (s, 12H). ^{11}B NMR (160 MHz, C_6D_6): δ(ppm) = 23.8.

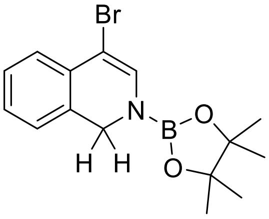

8j

4-bromo-1-(4,4,5,5-tetramethyl-1,3,2-dioxaborolan-2-yl)-1,2-dihydroisoquinoline (8j). ^{1}H NMR (500 MHz, C_6D_6): δ(ppm) = 7.53 – 7.43 (m, 1H), 7.01 – 6.97 (m, 1H), 6.90 – 6.82 (m, 1H), 6.63 – 6.56 (m, 1H), 4.45 (s, 2H), 1.0 (s, 12H). ^{11}B NMR (160 MHz, C_6D_6): δ(ppm) = 23.6.

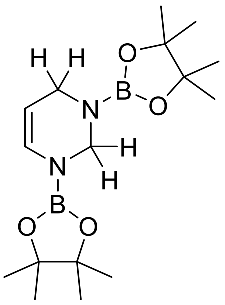

8k

1,3-bis(4,4,5,5-tetramethyl-1,3,2-dioxaborolan-2-yl)-1,2,3,4-tetrahydropyrimidine (8k). ^{1}H NMR (500 MHz, C_6D_6): δ(ppm) = 6.80 (d, J = 6.5 Hz, 2H), 4.65 – 4.62 (m, 2H), 3.87 – 3.75 (m, 2H), 1.06 (s, 12H), 1.02 (s, 12H). ^{11}B NMR (160 MHz, C_6D_6): δ(ppm) = 23.9.

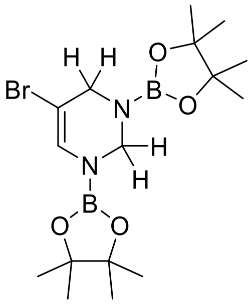

8l

5-bromo-1,3-bis(4,4,5,5-tetramethyl-1,3,2-dioxaborolan-2-yl)-1,2,3,4-tetrahydropyrimidine (8l). ^{1}H NMR (500 MHz, C_6D_6): δ(ppm) = 7.09 – 7.06 (m, 1H), 4.57 (s, 2H), 3.99 (d, J = 1.2 Hz, 2H), 1.02 (s, 12H). ^{11}B NMR (160 MHz, C_6D_6): δ(ppm) = 23.6.

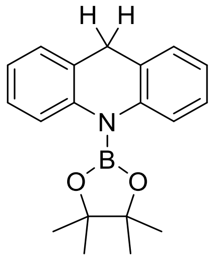

8m

10-(4,4,5,5-tetramethyl-1,3,2-dioxaborolan-2-yl)-4a,9,9a,10-tetrahydroacridine (8m). ^{1}H NMR (500 MHz, C_6D_6): δ(ppm) = 7.84 (d, J = 8.1 Hz, 2H), 7.20 – 7.13 (m, 2H), 7.03 – 6.91 (m, 4H), 3.55 (s, 2H), 1.07 (s, 12H). ^{11}B NMR (160 MHz, C_6D_6): δ(ppm) = 25.0.

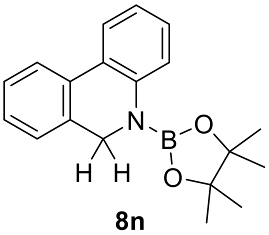

8n

5-(4,4,5,5-Tetramethyl-1,3,2-dioxaborolan-2-yl)-5,6-dihydrophenanthridine (8n). ^{1}H NMR (500 MHz, C_6D_6): δ(ppm) = 7.92 (d, *J* = 8.1 Hz, 1H), 7.65 (d, *J* = 7.6 Hz, 1H), 7.54 (d, *J* = 7.7 Hz, 1H), 7.20 (t, *J* = 7.6 Hz, 1H), 7.10 (t, *J* = 7.4 Hz, 1H), 7.02 – 6.91 (m, 3H), 4.57 (s, 2H), 1.03 (s, 12H). ^{11}B NMR (160 MHz, C_6D_6): δ(ppm) = 24.0.

3.6 Mechanistic studies

3.6.1 General procedure for the synthesis of DBpin

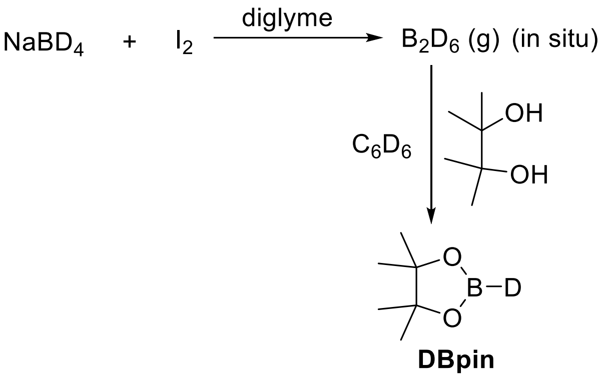

DBpin was synthesized according to literature procedure.[8]

^{1}H NMR (500 MHz, C_6D_6): δ(ppm) = 1.00 (s, 9H, CH_3). **^{11}B NMR** (160 MHz, C_6D_6): δ 27.2.

3.6.2 Deuterium labelling experiment

In a J. young NMR tube, quinoline (0.1 mmol), DBpin (0.2 mmol, 2.0 equiv) and 5 mol% of **4** (4.4 mg, 5.0 µmol) was added. To this 0.45 mL of C_6D_6 was added. The NMR tube was taken out and heated at 50 °C for 24 h. The reaction was monitored by ^{1}H NMR spectroscopy.

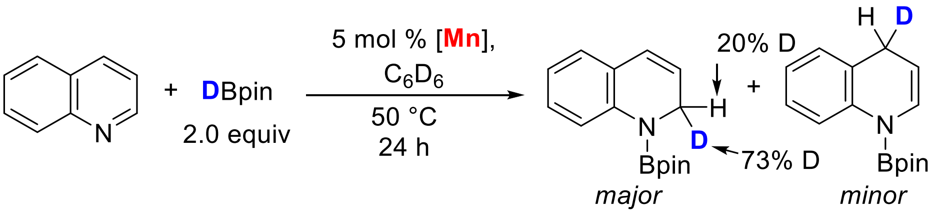

^{1}H NMR (500 MHz, C_6D_6): δ(ppm) = 7.82 (d, J = 8.1 Hz, 1H), 7.12 – 7.08 (m, 1H), 6.86 – 6.78 (m, 2H), 6.26 (d, J = 9.5 Hz, 1H), 5.57 (dd, J = 9.5, 4.1 Hz, 1H), 4.15 (d, J = 5.7 Hz, 0.27H), 4.11 (d, J = 1.7 Hz, 0.79), 1.05 (s, 12H). **^{2}H NMR** (77 MHz, C_6D_6) δ(ppm) = 4.12 (d, J = 2.2 Hz, 1H), 3.25 (d, J = 3.5 Hz, 0.14H).

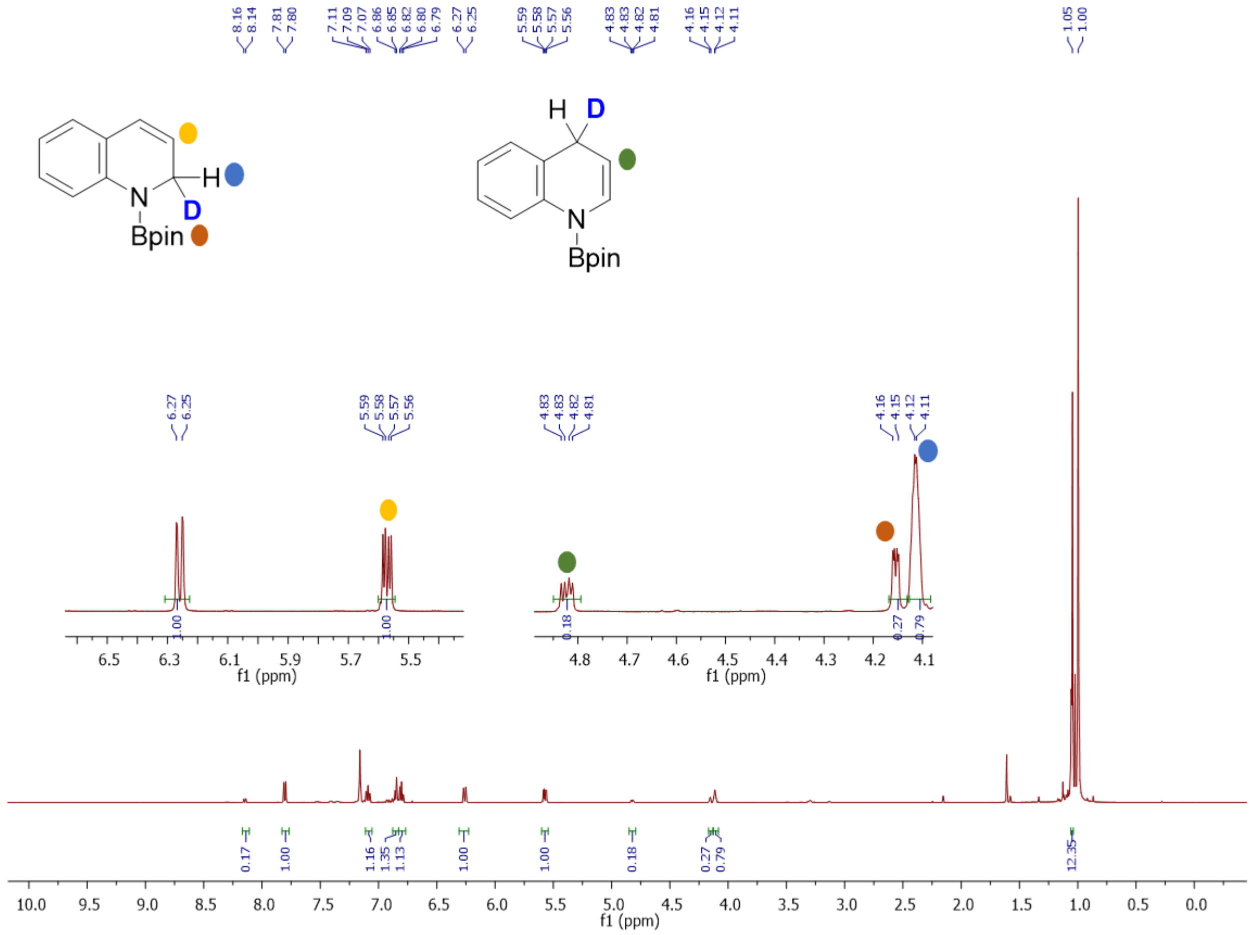

Figure S25: ^{1}H NMR (500 MHz, C_6D_6) spectrum of the deuterium-labelling experiment for quinoline with DBpin using [SiNSi]Mn(dmpe) (**4**) as a pre-catalyst.

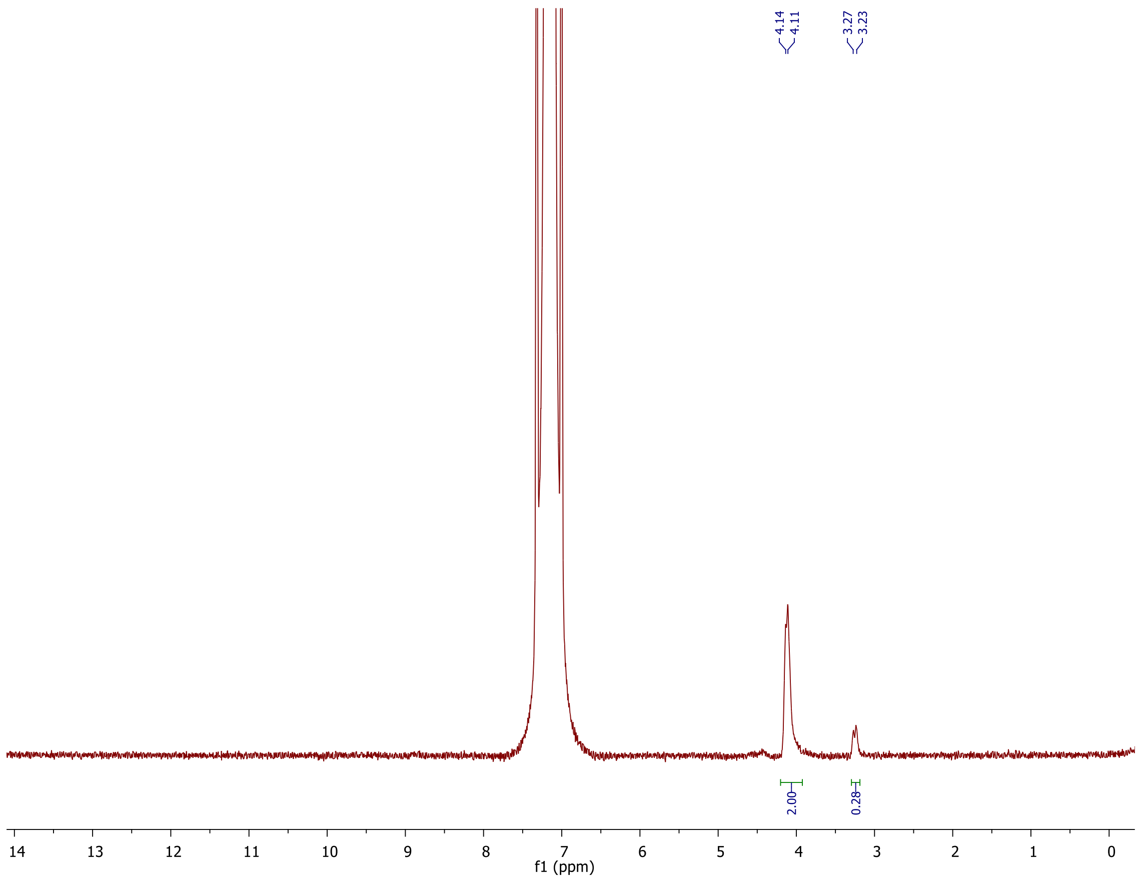

Figure S26: ^{2}H-NMR (77 MHz, C_6D_6) spectrum of the deuterium-labelling experiment for quinoline with DBpin using [SiNSi]Mn(dmpe) (**4**) as a precatalyst

3.6.3 NMR study of reaction of 4 with pinacolborane (HBpin)

In a N_2 filled glove box, 20 mg **4** (0.02 mmol) and 11.5 µL of HBpin (0.08 mmol), were dissolved in 0.5 mL C_6D_6 in a J. Young NMR tube. The NMR tube was taken out and heated at 50 °C. the reaction progress was monitored by ^{1}H-NMR. After 6 h, formation of a new diamagnetic species was indicated in ^{1}H NMR spectrum with a signal at -9.6 ppm (Figure S32). ^{1}H-^{31}P HMQC NMR spectrum exhibited a correlation NMR signal corresponding to the hydridic signal (Figure S28).

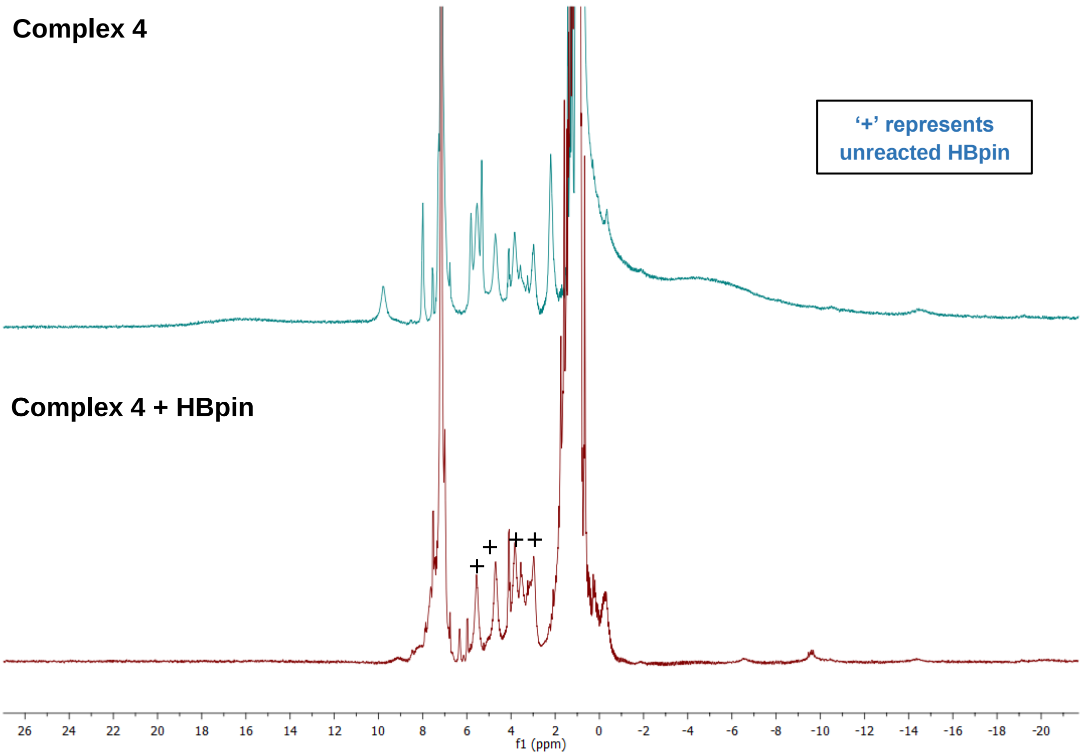

Figure S27: ^{1}H-NMR (200 MHz, C_6D_6) spectrum of the reaction mixture from stoichiometric reaction of HBpin with [SiNSi]Mn(dmpe) (**4**).

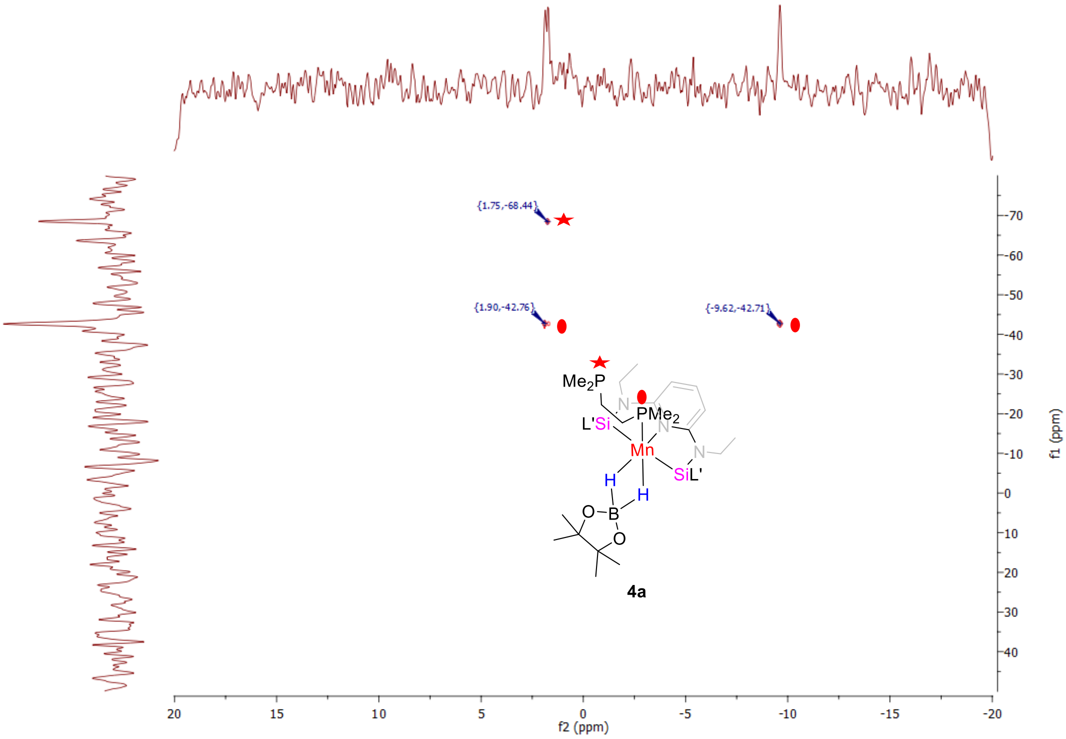

Figure S28: ^{1}H-^{31}P HMQC NMR (400 MHz, C_6D_6) spectrum of the reaction mixture from stoichiometric reaction of HBpin with [SiNSi]Mn(dmpe) (**4**).

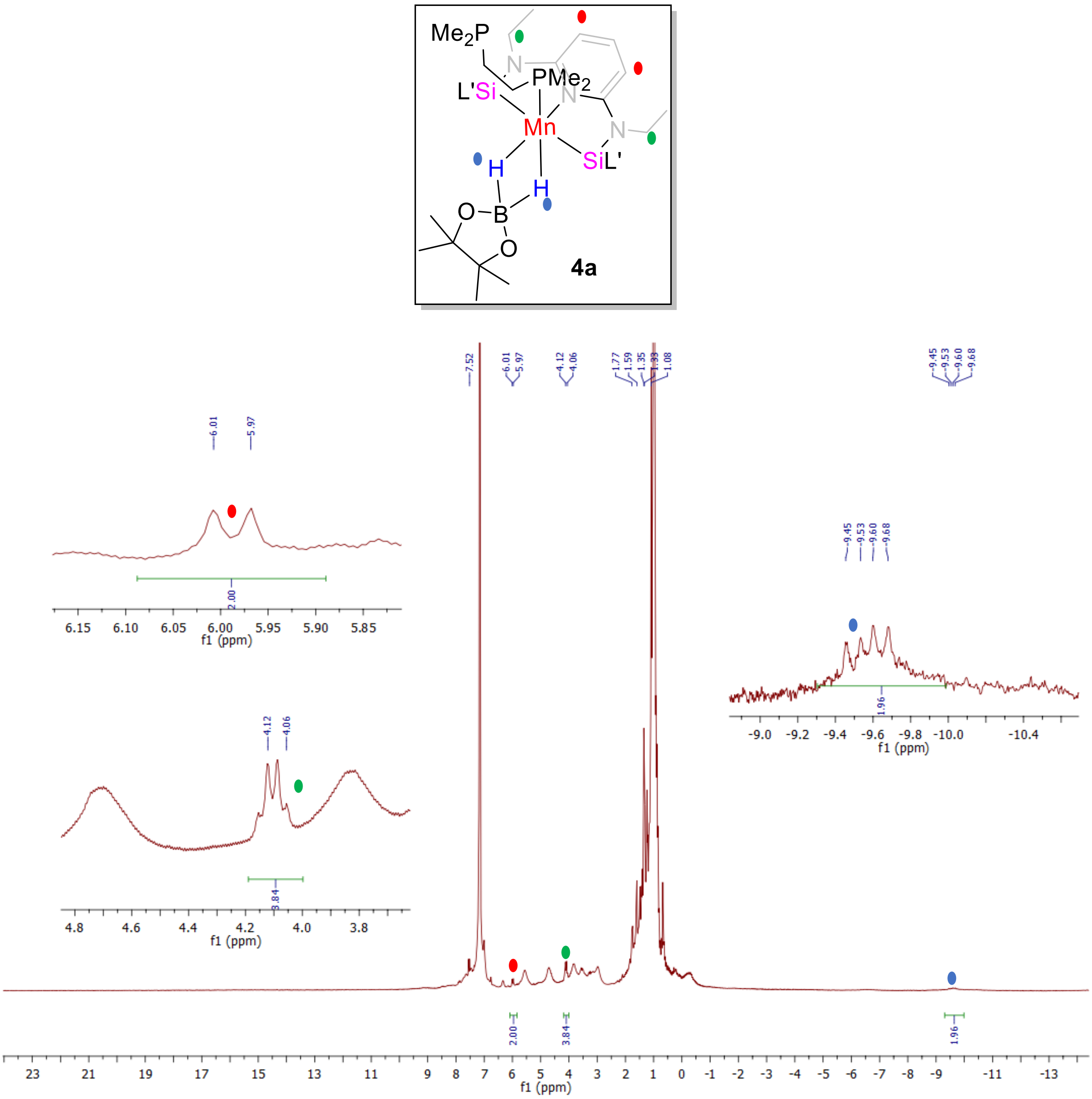

Figure S29: ^{1}H NMR (200 MHz, C_6D_6) spectrum of the reaction mixture from stoichiometric reaction of HBpin with [SiNSi]Mn(dmpe) (**4**).

3.6.4 Kinetic isotope effect:

Two NMR-samples were prepared, each containing 5 mol% of **4** (4.4 mg, 5.0 µmol) in 0.5 mL C_6D_6 containing mesitylene as internal standard (0.05 mmol, 6.9 µL), quinoline (0.1 mmol, 12.8 µL) and 2.0 equivalents of HBpin or DBpin. ^{1}H-NMR spectra were obtained at regular intervals approx. 1 h and the NMR tubes were shaken after each measurement. The concentration of quinoline was plotted against time and the data points were fitted with a linear function (R^2 = 0.99/0.98).

	**4**	quinoline	HBpin	mesitylene
millimoles	0.005	0.10	0.20	0.05
Concentration (mol/L)	0.010	0.22	0.40	0.10

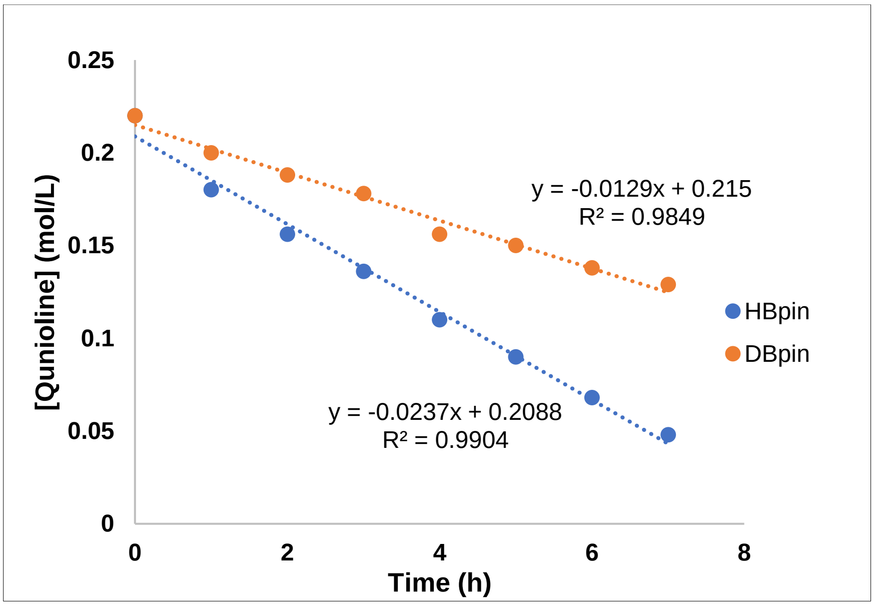

Figure S30: KIE for hydroboration of quinoline.

The KIE was calculated using the following equation:

$$KIE = \frac{k_H}{k_D} = \frac{-0.0237}{-0.0129} = 1.84$$

4 Cyclic voltammetry experiments

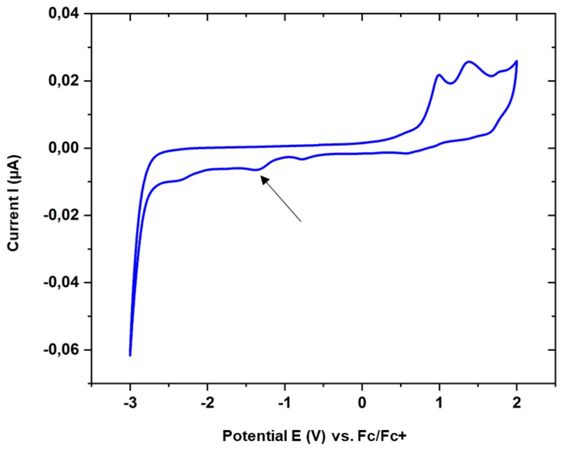

(a)

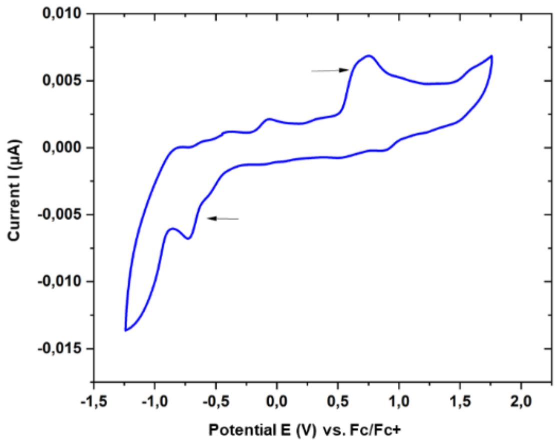

(b)

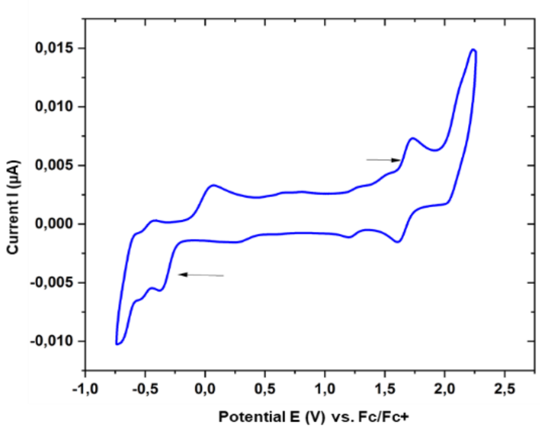

(c)

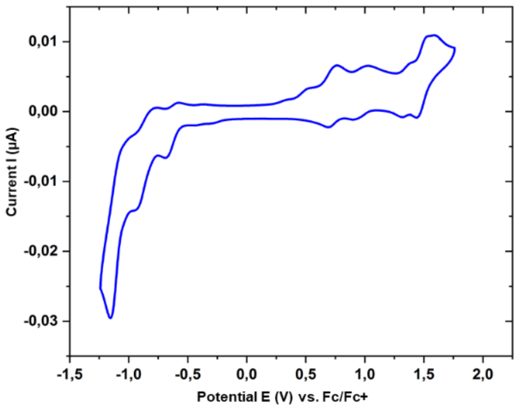

(d)

Figure S31: CV of complex **2** (a), complex **3** (b), complex **5** (c) and complex **6** (d) (1 mM in THF/ 0.3 M $TBAPF_6$) recorded at a scan rate v = 100 mV·s

5 Selected NMR spectrum

Signals designated by (*) in ^{1}H-NMR correspond to mesitylene (internal standard) and (+) in both ^{1}H-NMR and ^{11}B-NMR correspond to the unreacted HBpin.

8a: ^{1}H-NMR (500 MHz, C_6D_6):

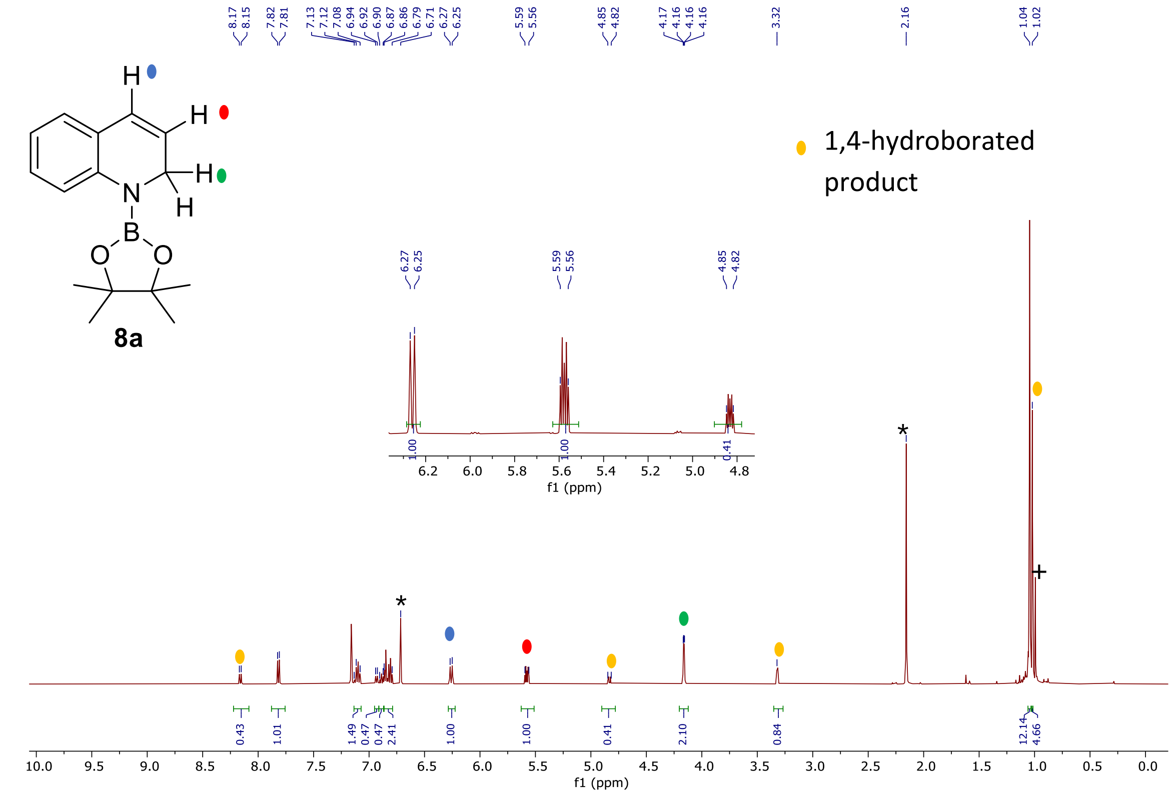

^{11}B{^{1}H}-NMR (160 MHz, C_6D_6):

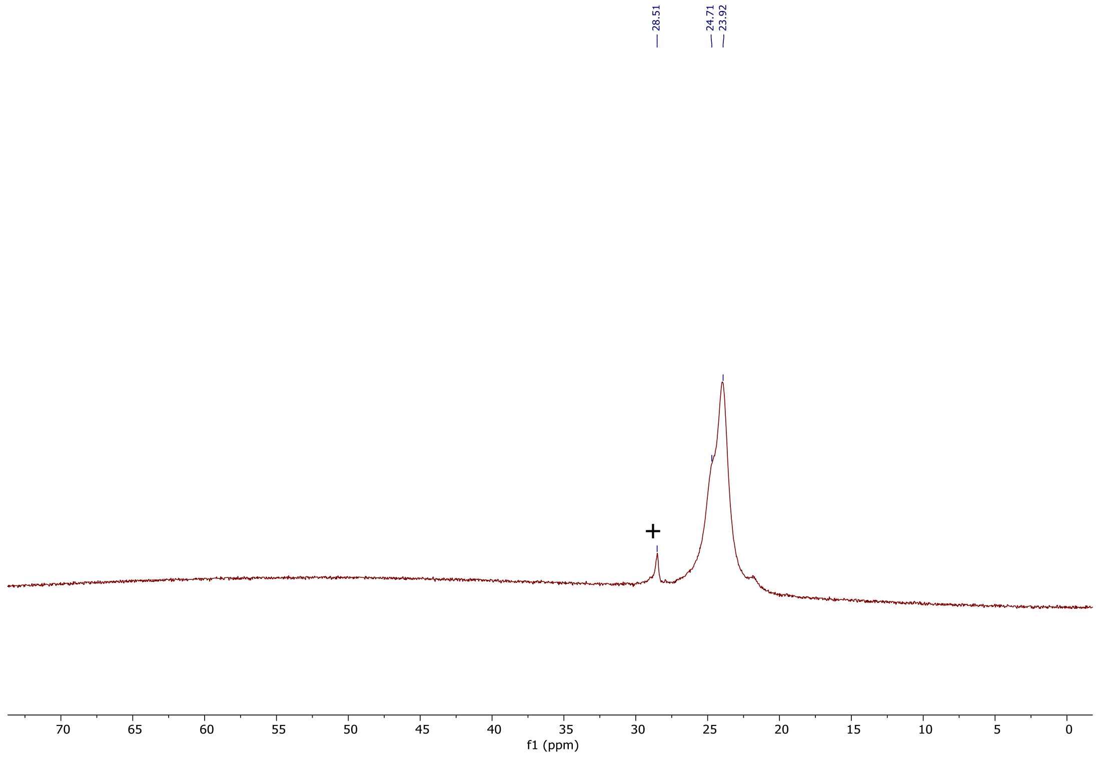

8b: ^{1}H-NMR (500 MHz, C_6D_6):

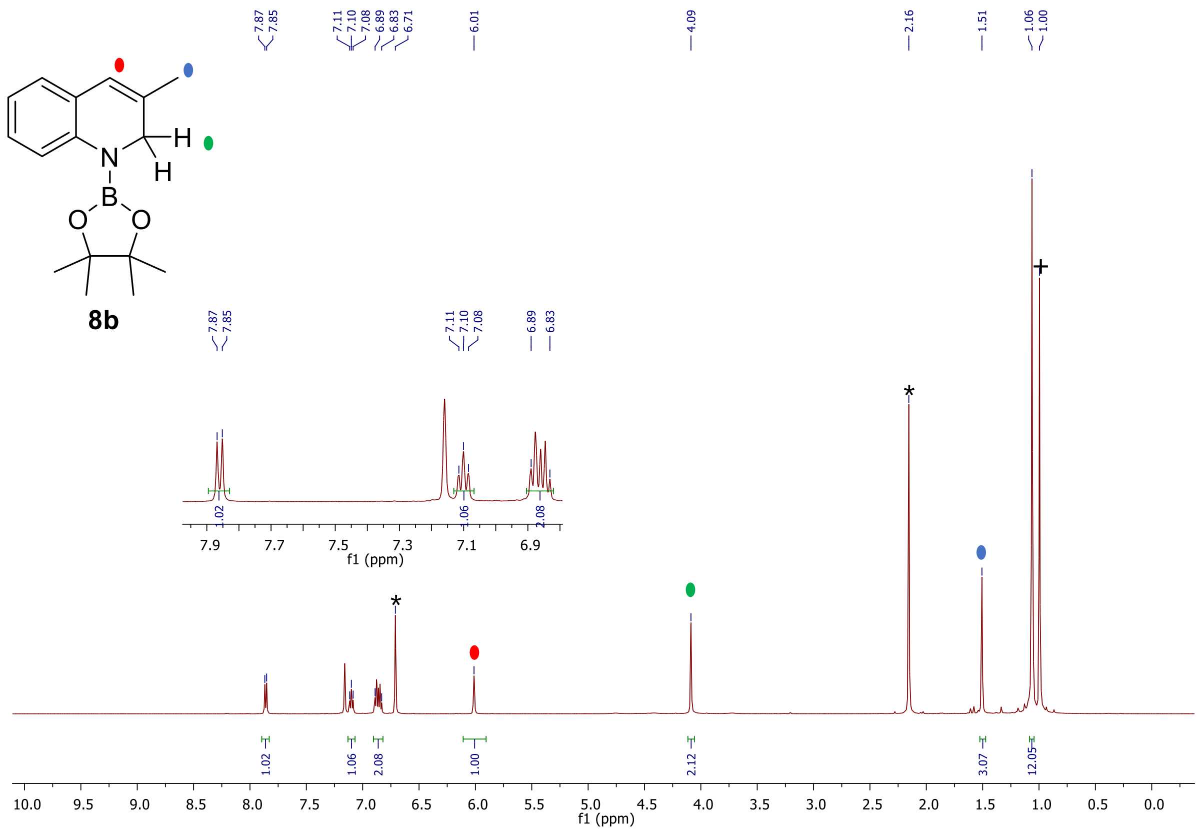

^{11}B{^{1}H}-NMR (160 MHz, C_6D_6):

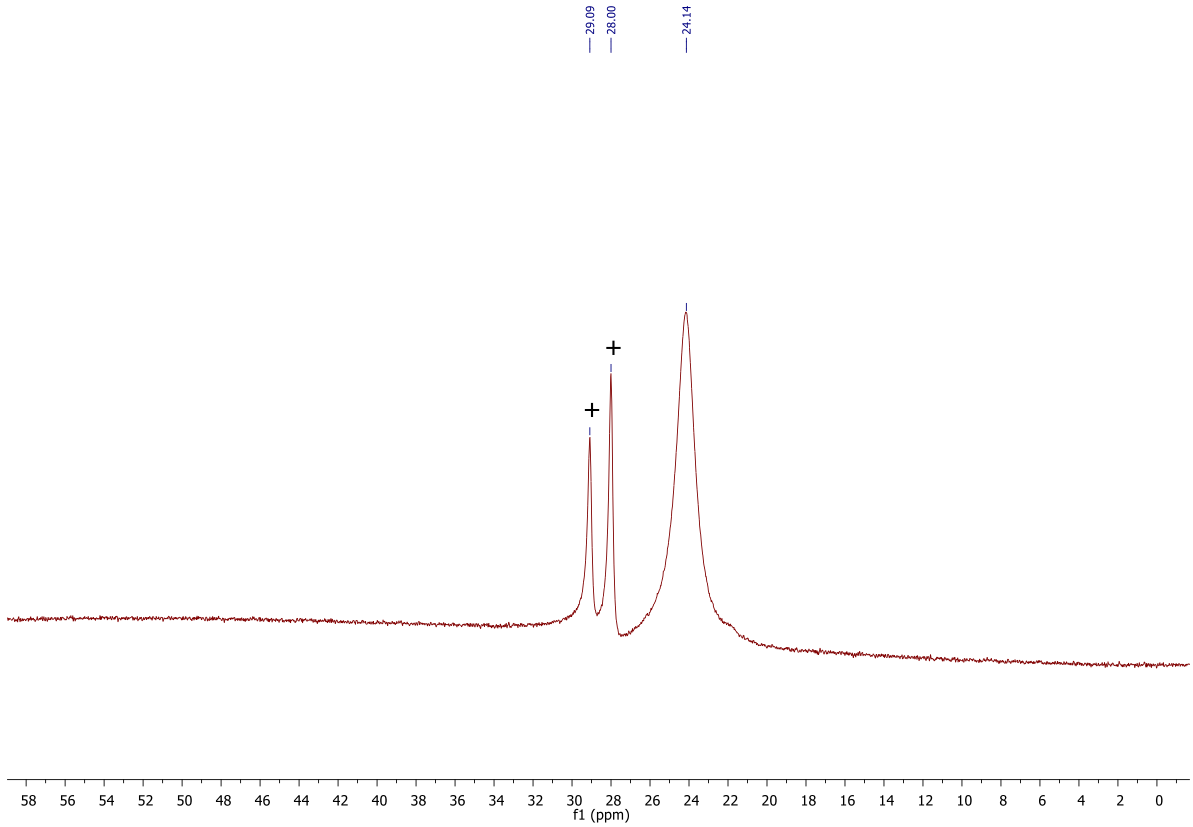

8c ^{1}H-NMR (500 MHz, C_6D_6):

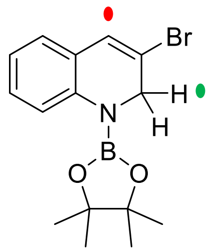

8c

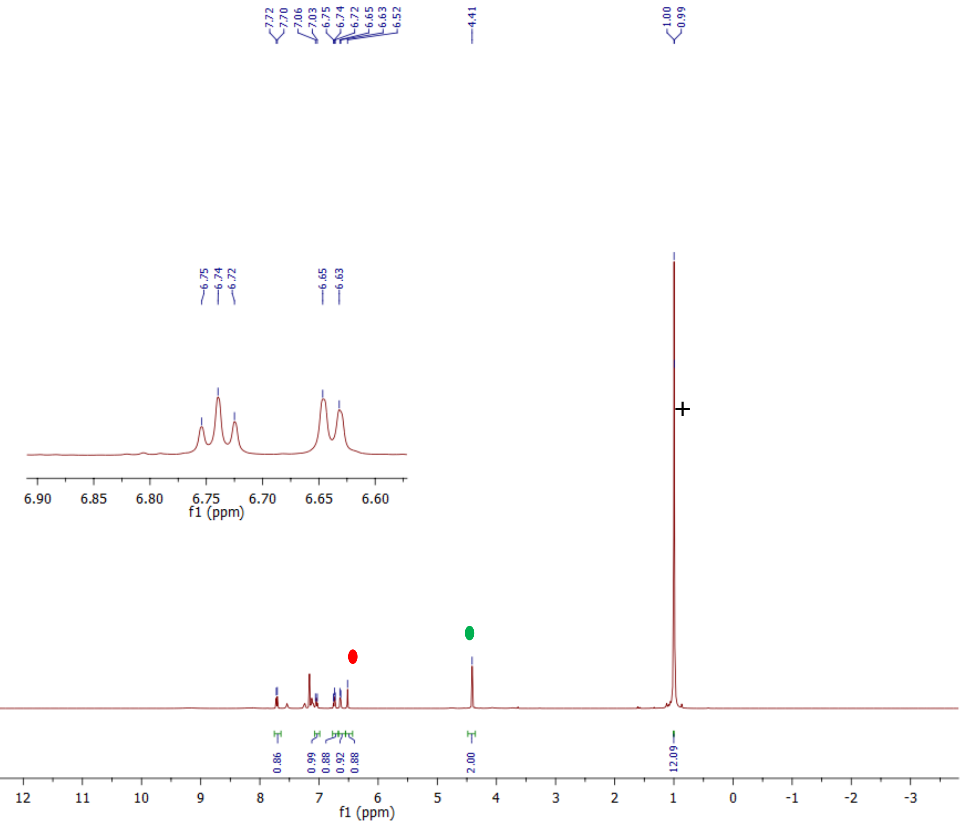

^{11}B{^{1}H}-NMR (160 MHz, C_6D_6):

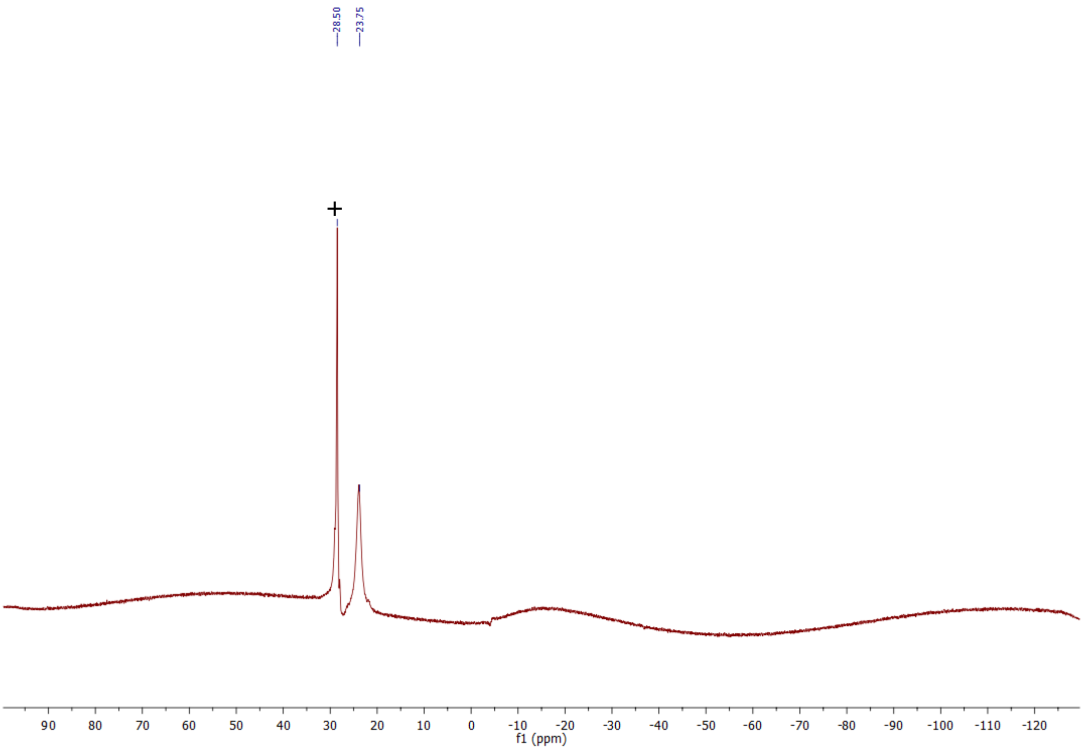

8d ^{1}H-NMR (500 MHz, C_6D_6):

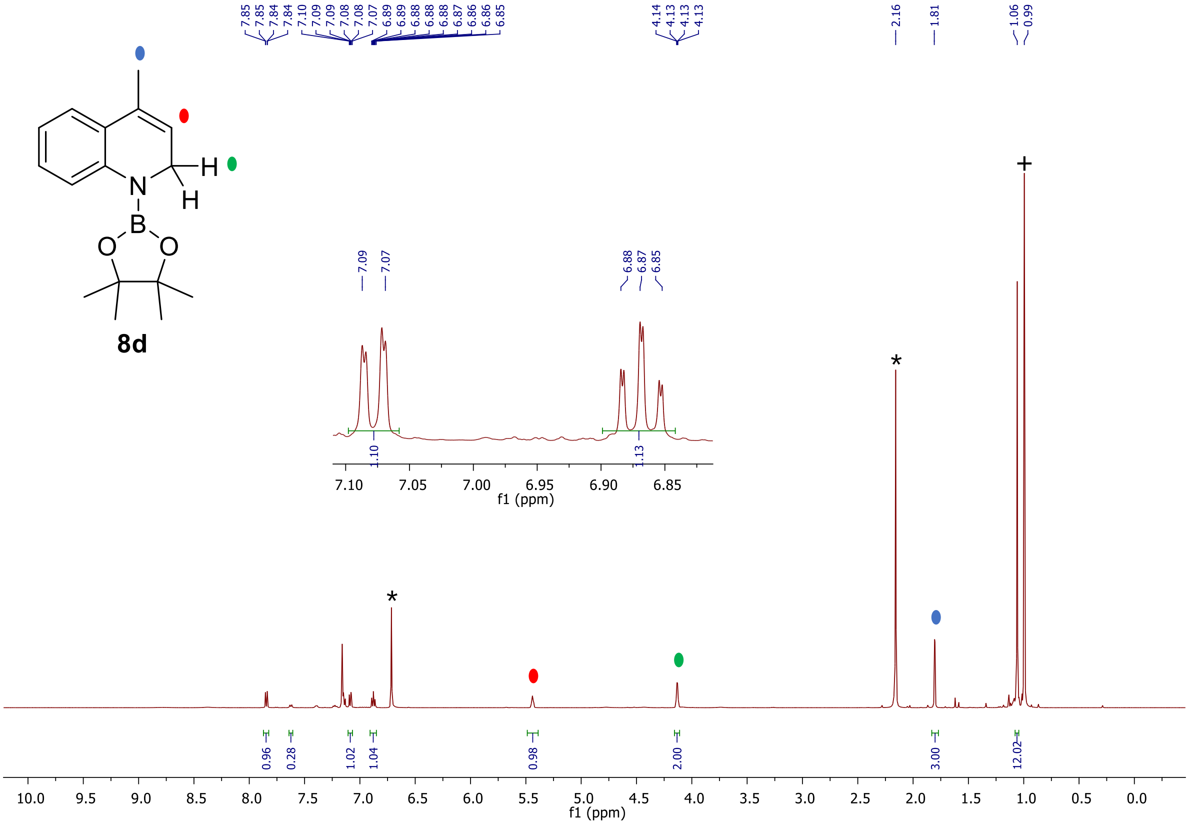

^{11}B-NMR (160 MHz, C_6D_6):

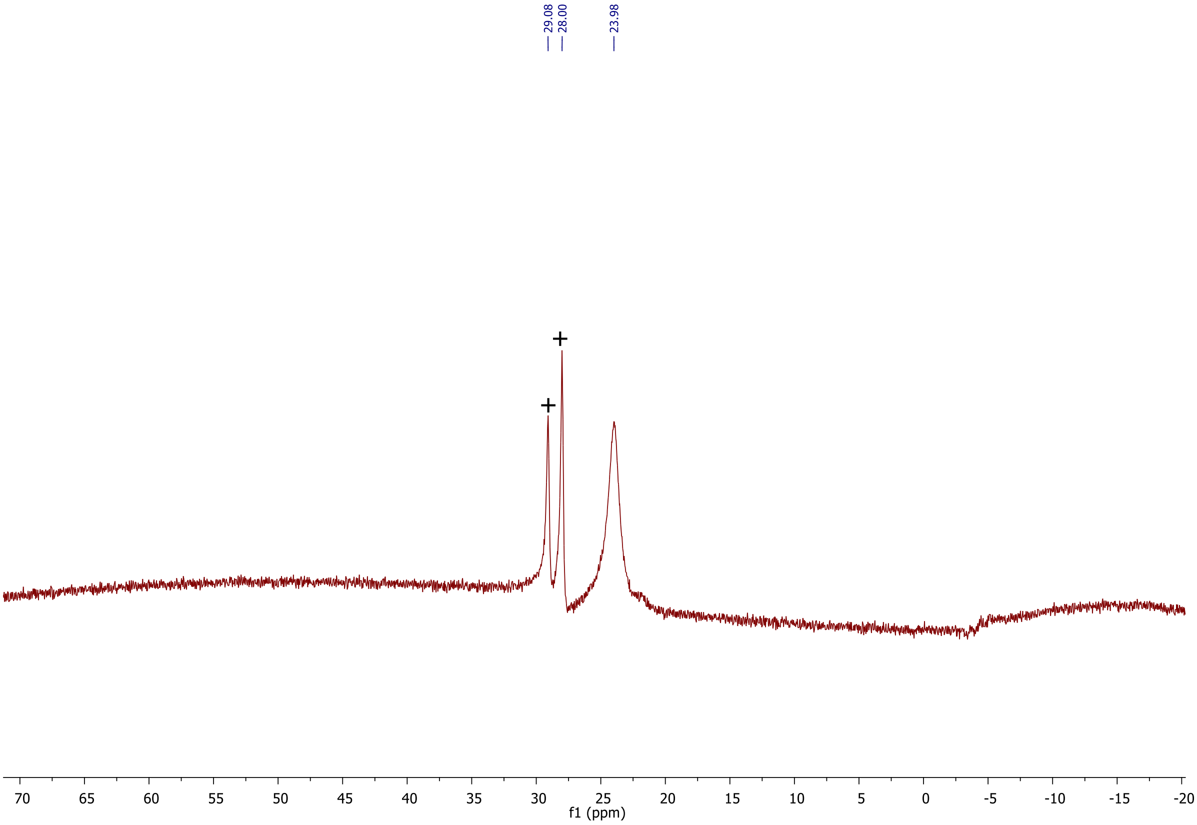

8e: ^{1}H-NMR (500 MHz, C_6D_6):

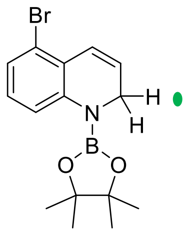

8e

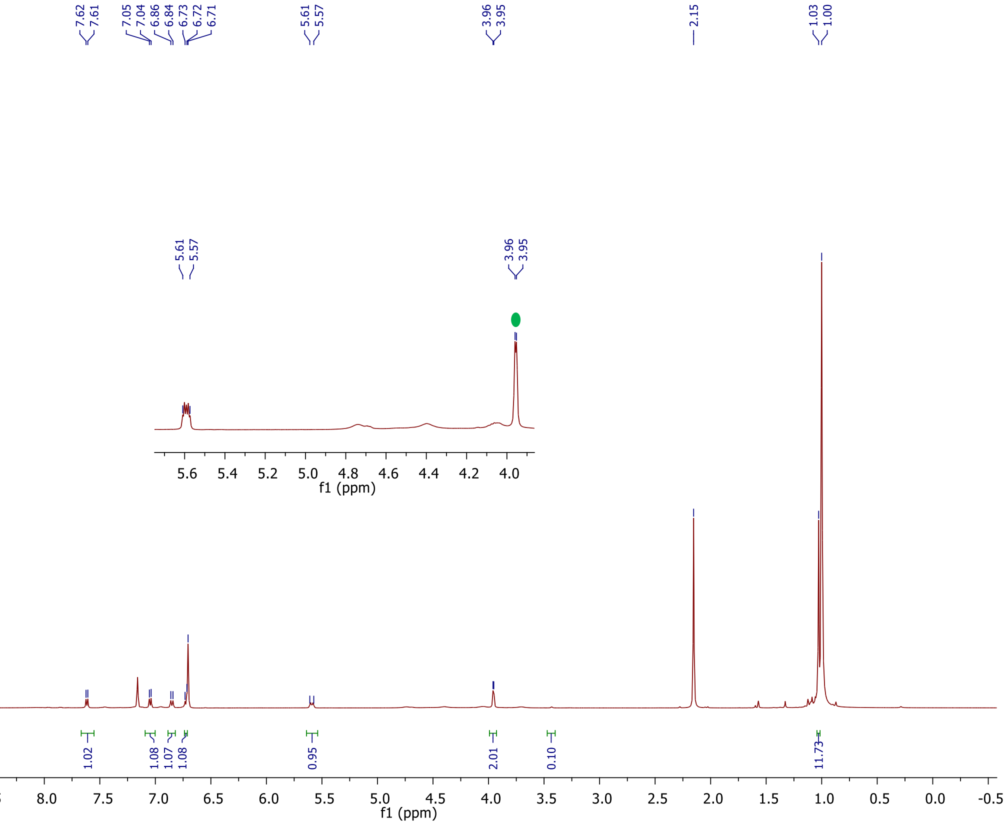

^{11}B-NMR (160 MHz, C_6D_6):

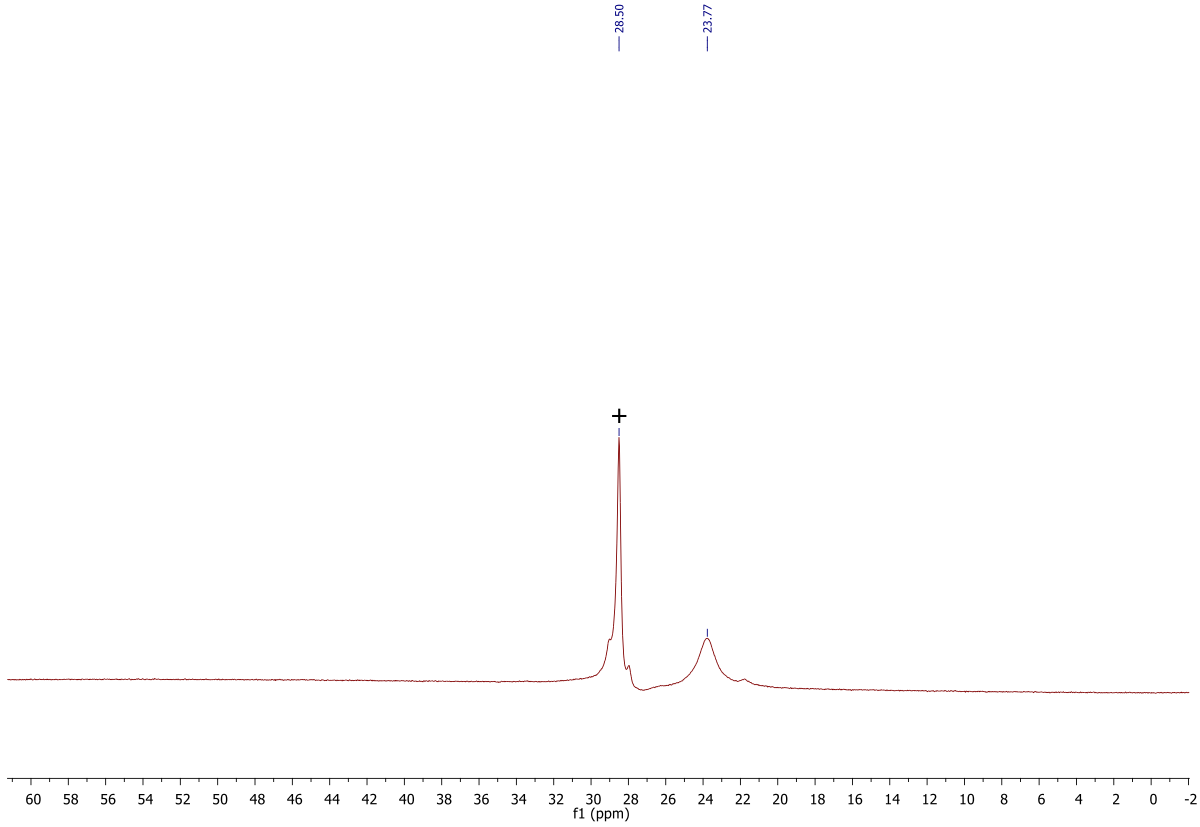

8f: ^{1}H-NMR (500 MHz, C_6D_6):

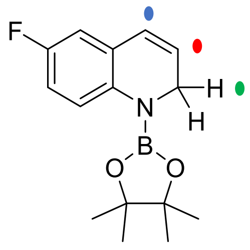

8f

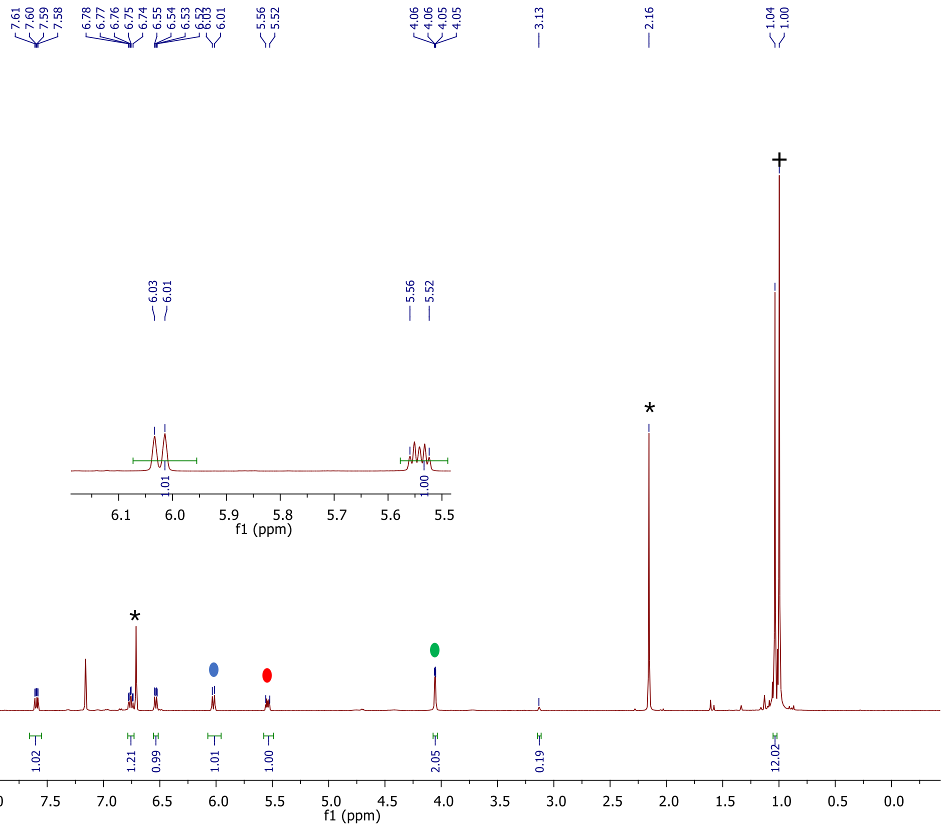

^{11}B-NMR (160 MHz, C_6D_6):

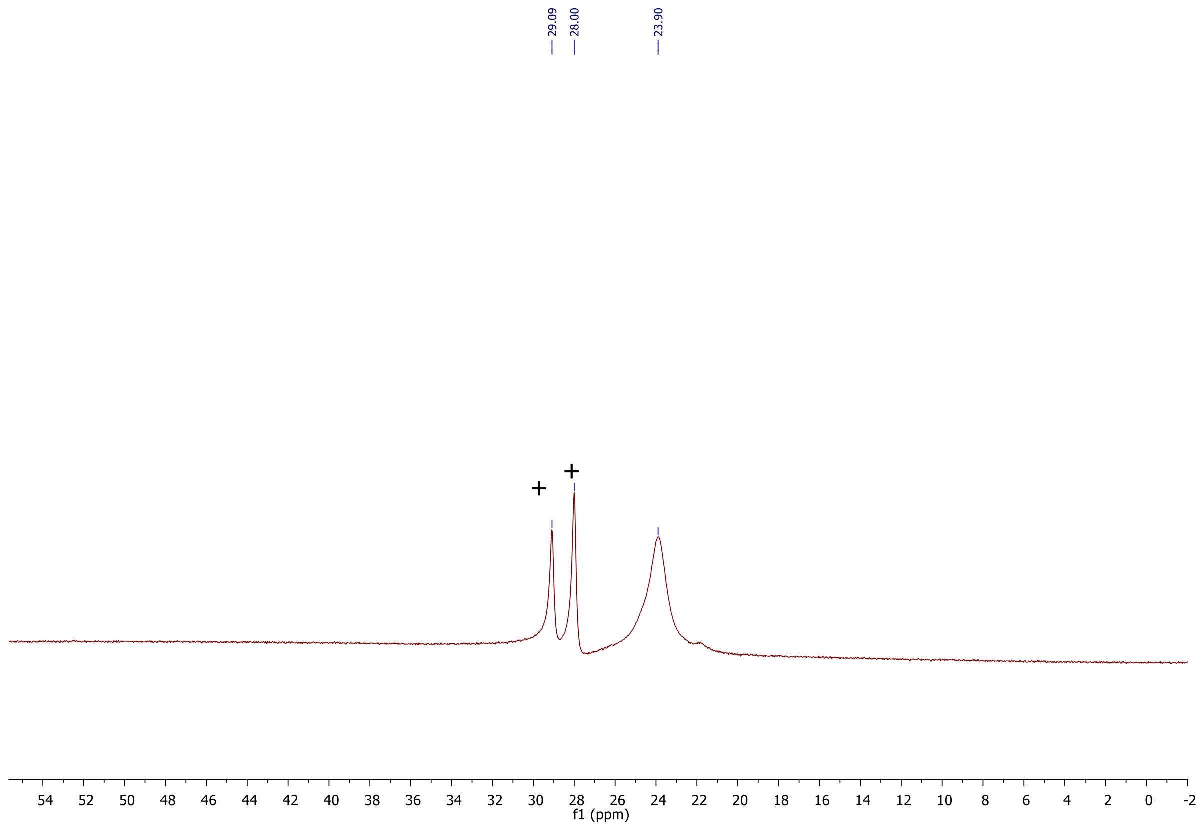

8g: ^{1}H-NMR (500 MHz, C_6D_6):

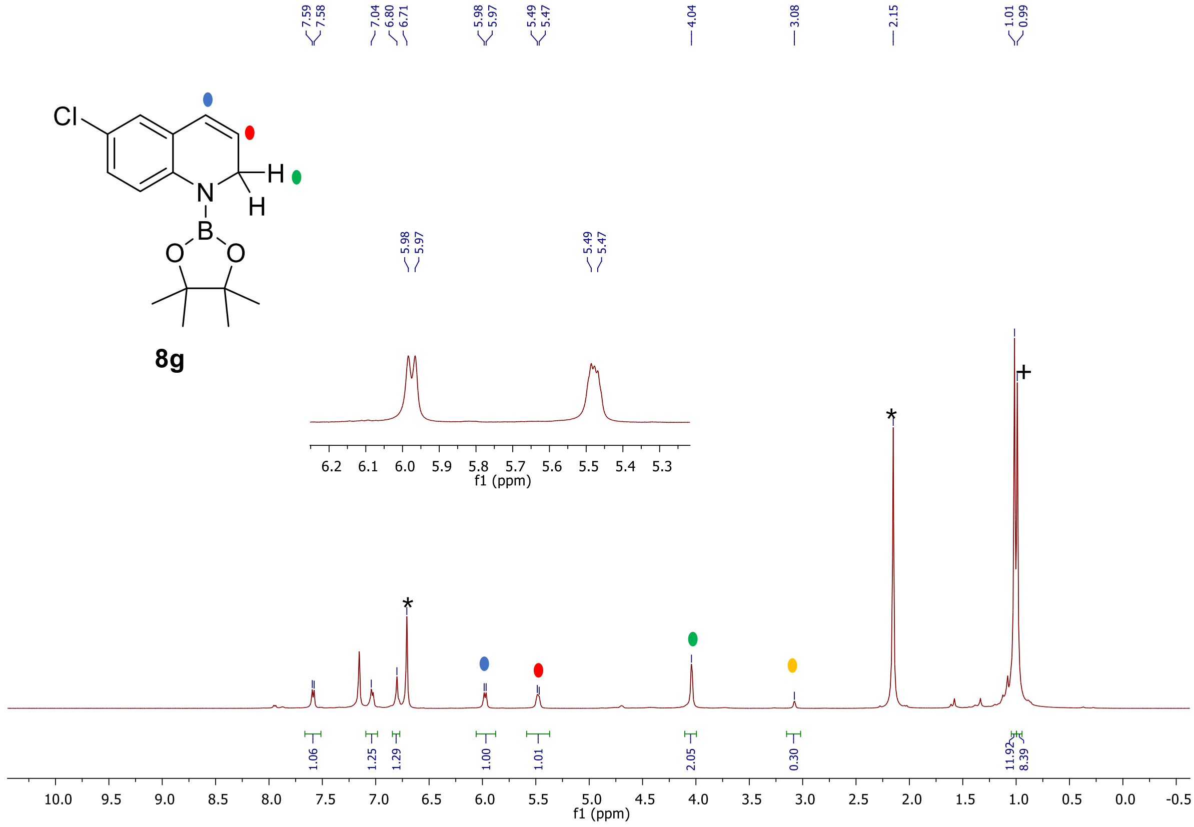

^{11}B-NMR (160 MHz, C_6D_6):

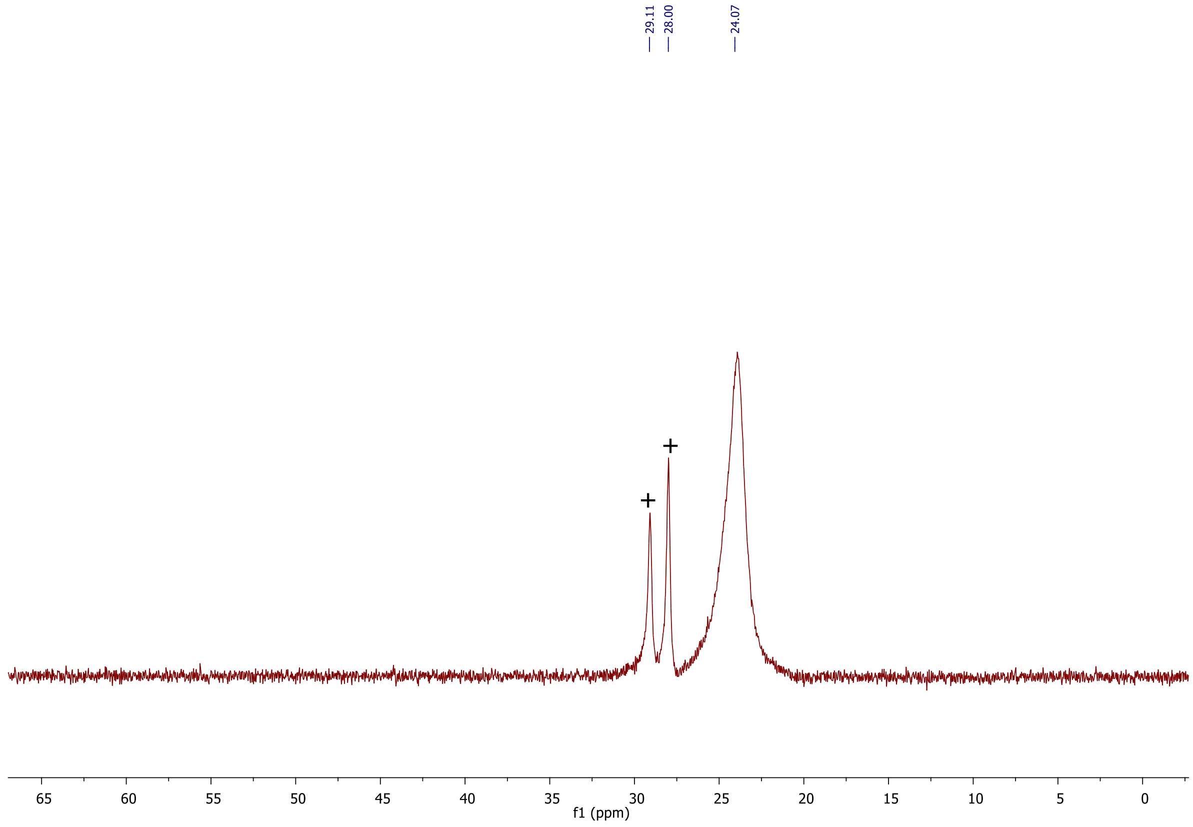

8h: ^{1}H-NMR (500 MHz, C_6D_6):

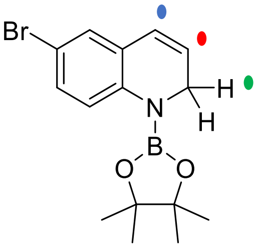

8h

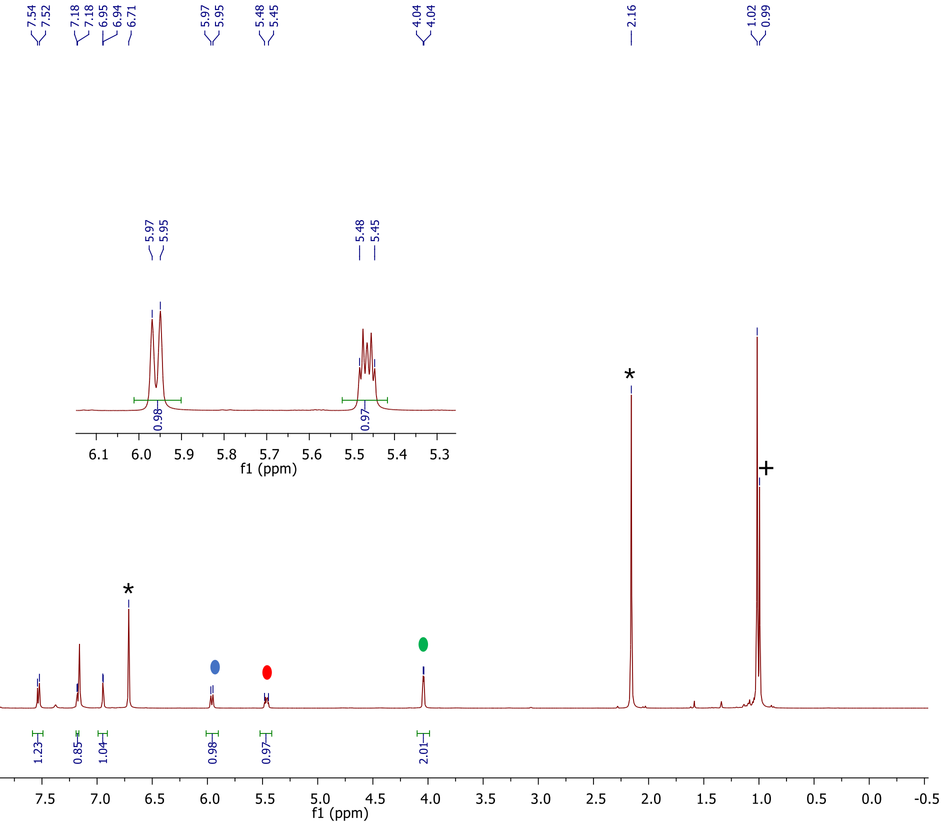

^{11}B{^{1}H}-NMR (160 MHz, C_6D_6):

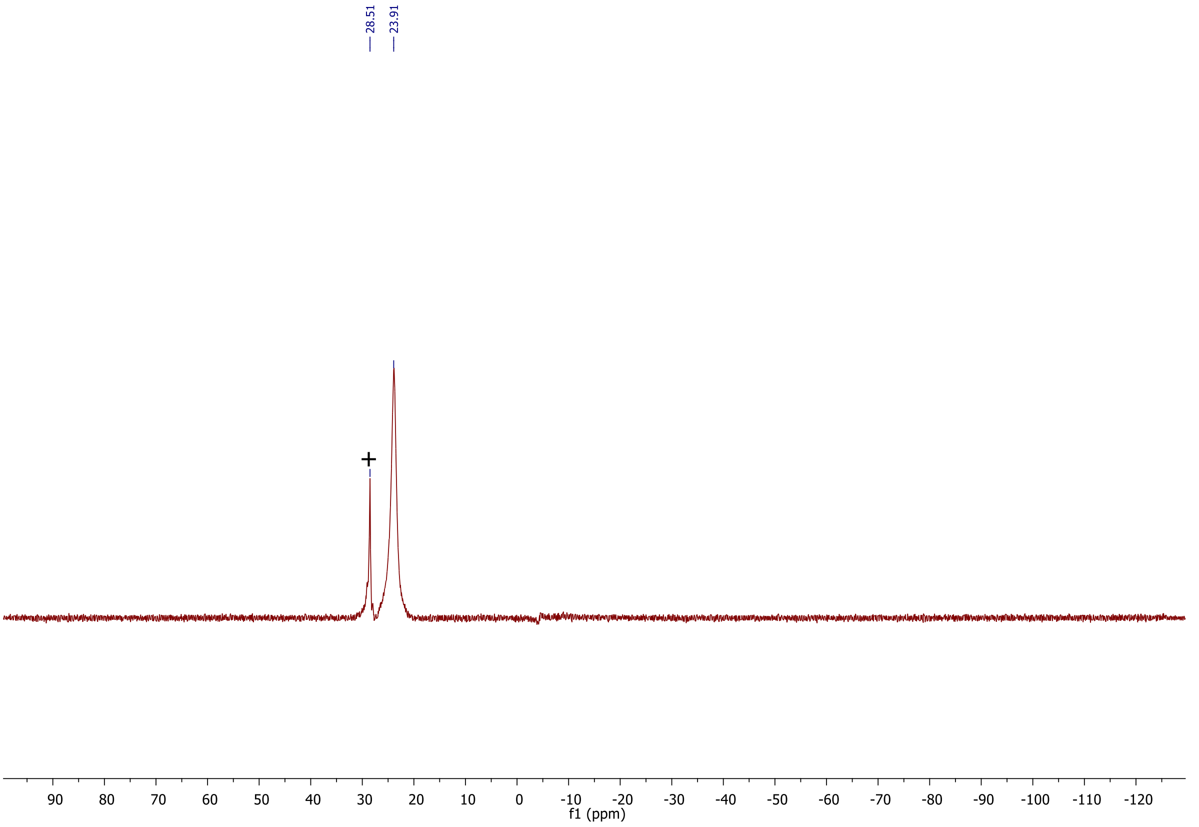

<u>8i</u>: ^{1}H-NMR (500 MHz, C_6D_6):

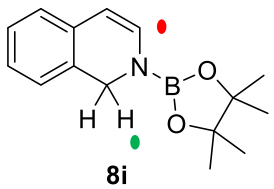

8i

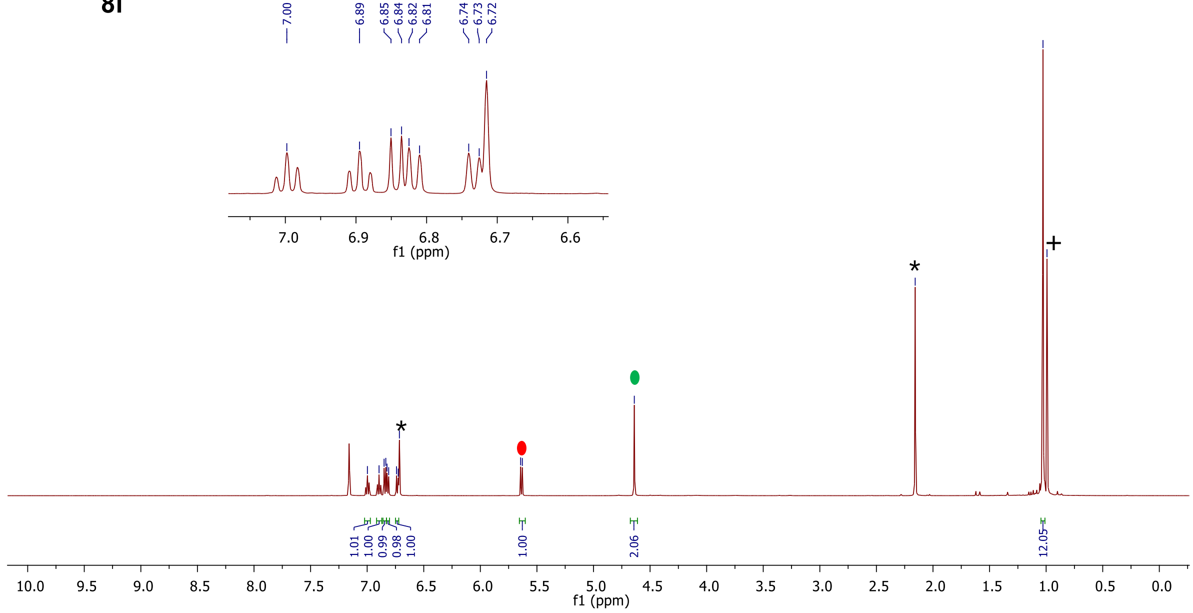

^{11}B{^{1}H}-NMR (160 MHz, C_6D_6):

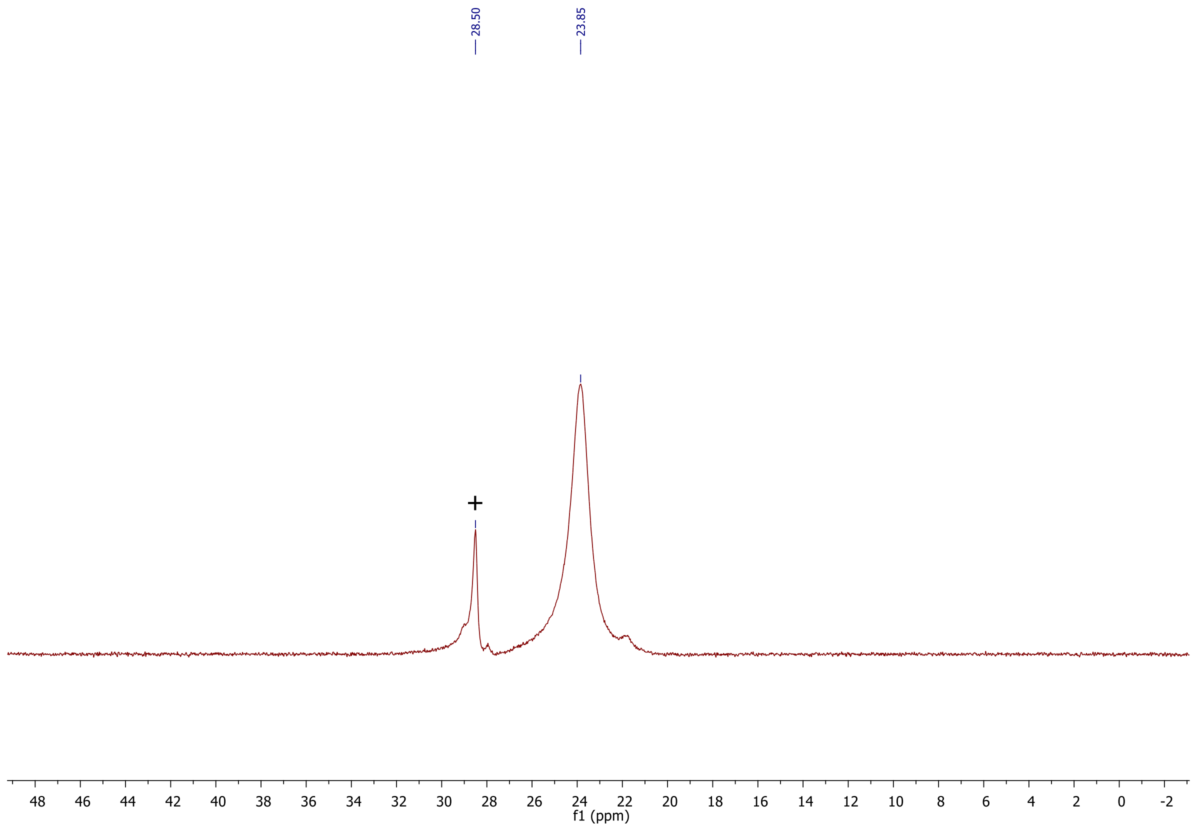

8j: ^{1}H-NMR (500 MHz, C_6D_6):

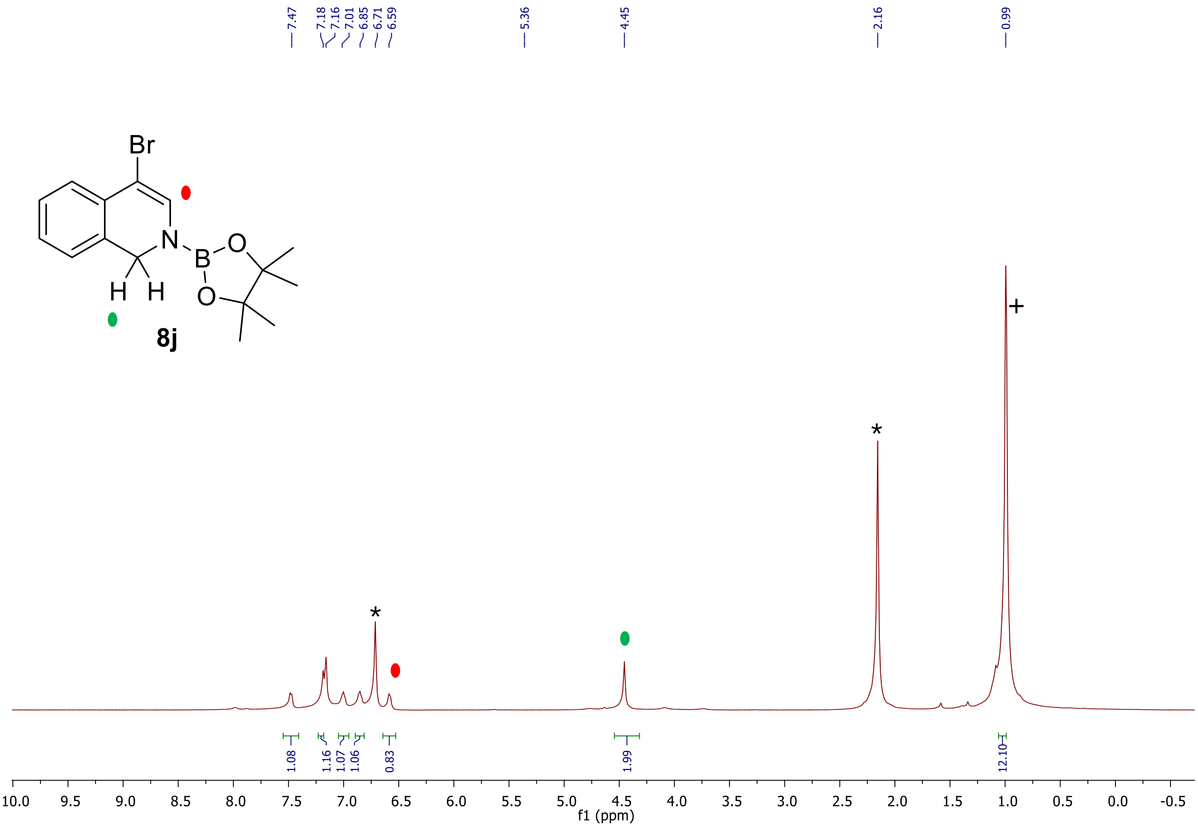

^{11}B-NMR (160 MHz, C_6D_6):

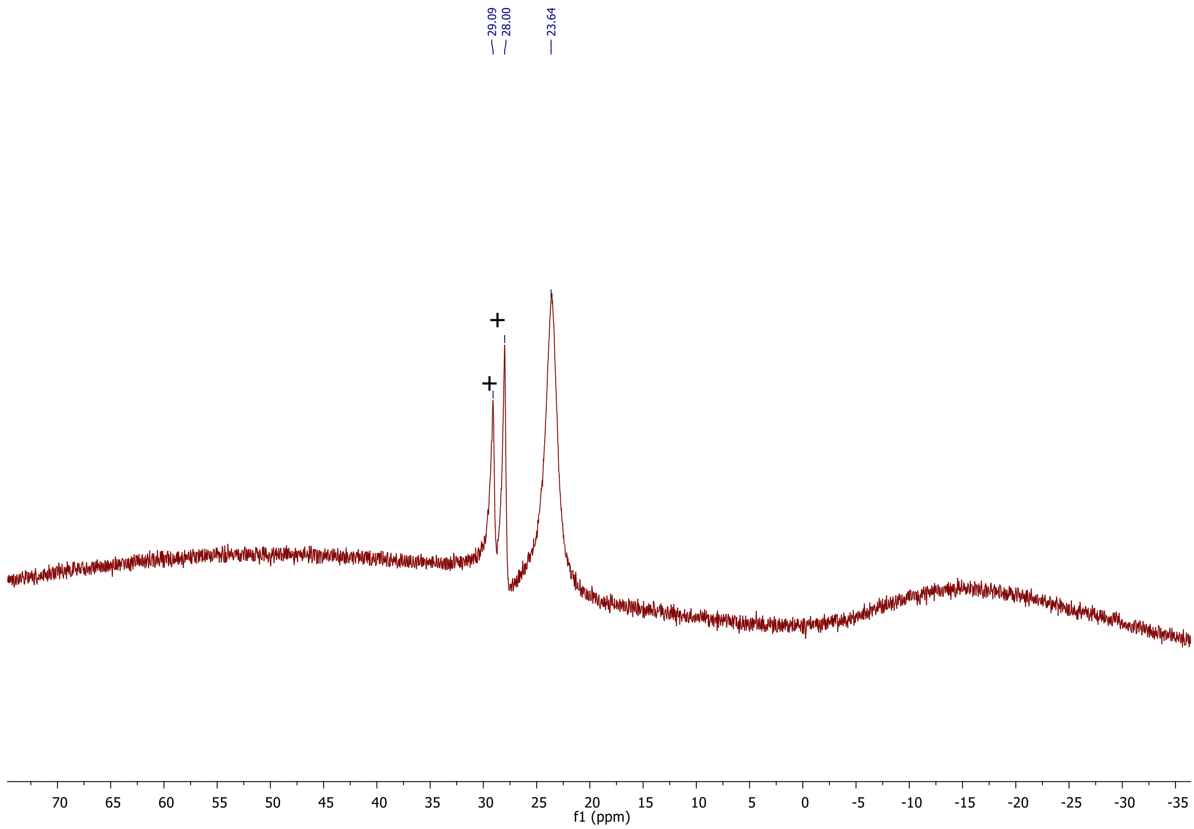

8k: ^{1}H-NMR (500 MHz, C_6D_6):

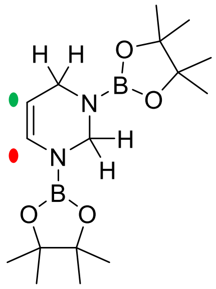

8k

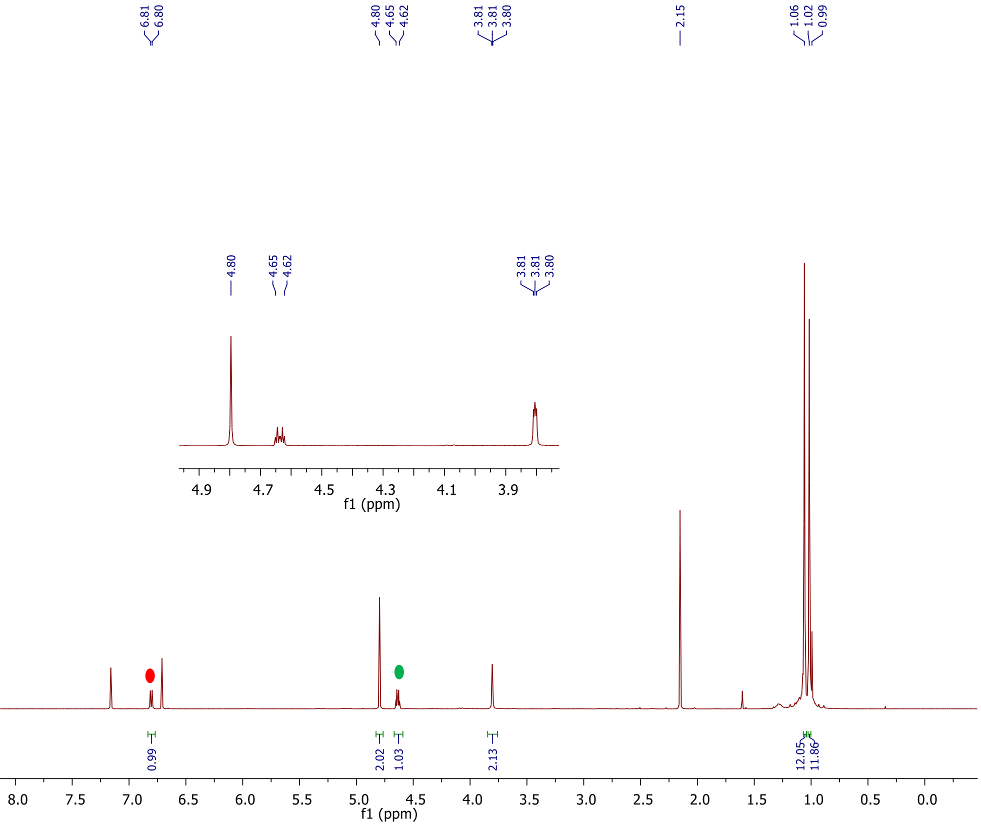

^{11}B-NMR (160 MHz, C_6D_6):

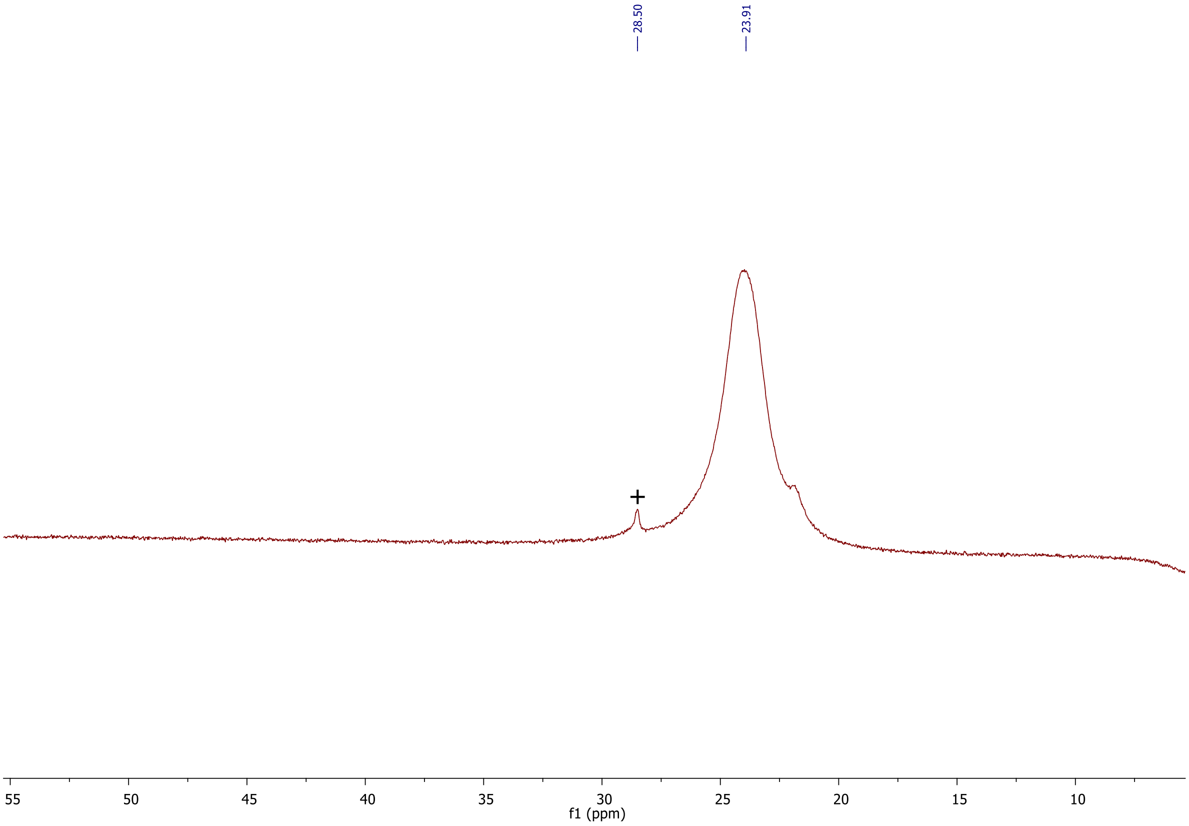

<u>8l</u>: ^{1}H-NMR (500 MHz, C_6D_6):

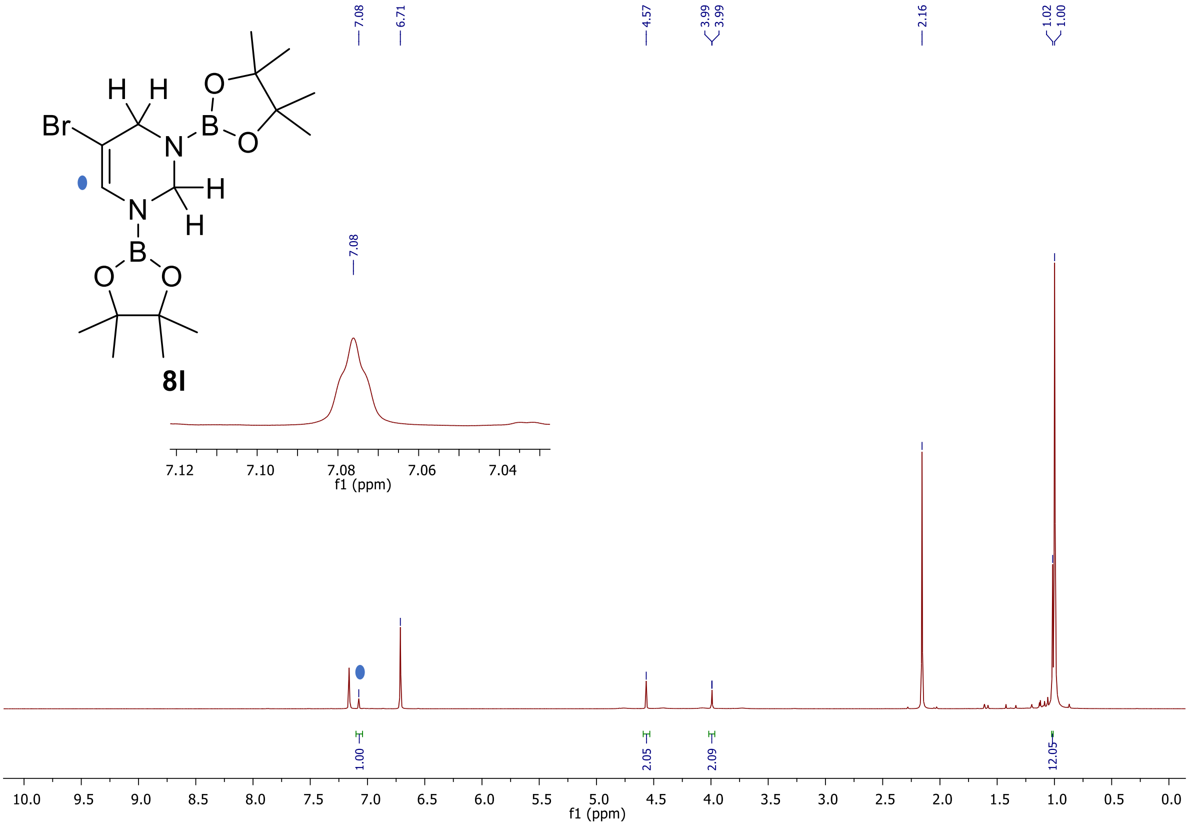

^{11}B-NMR (160 MHz, C_6D_6):

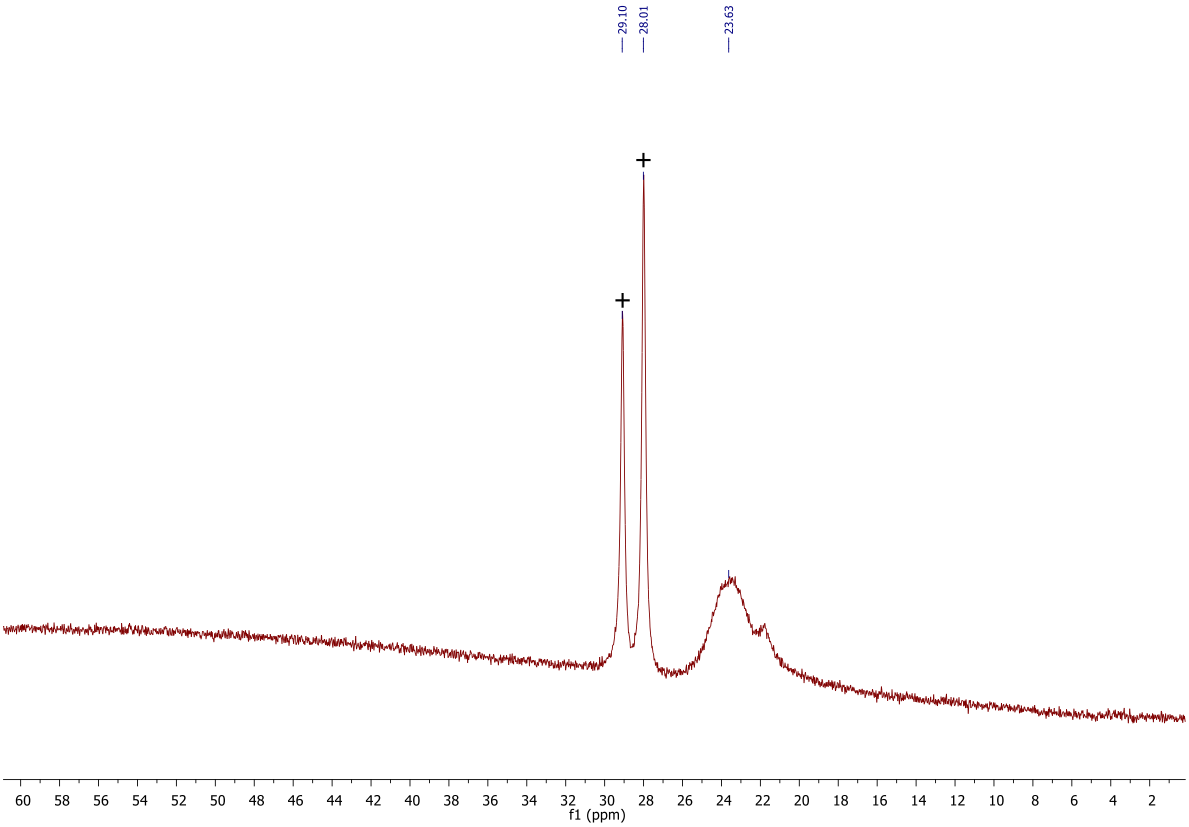

8m: ^{1}H-NMR (500 MHz, C_6D_6):

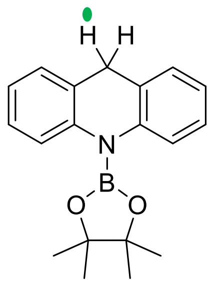

8m

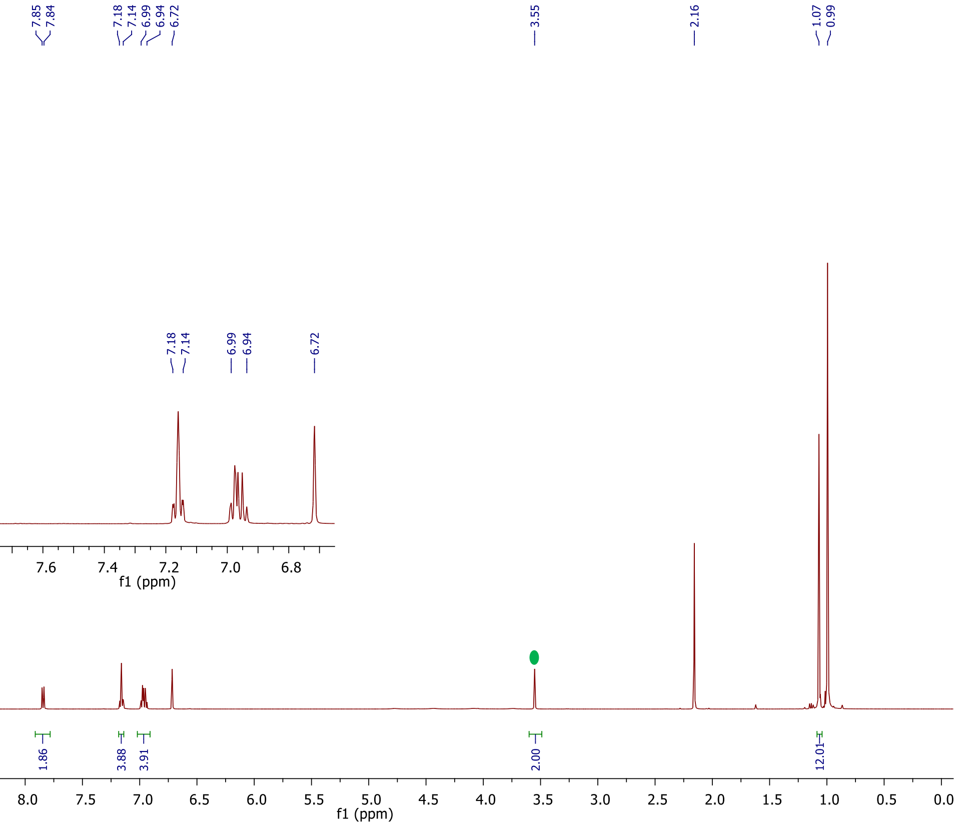

^{11}B-NMR (160 MHz, C_6D_6):

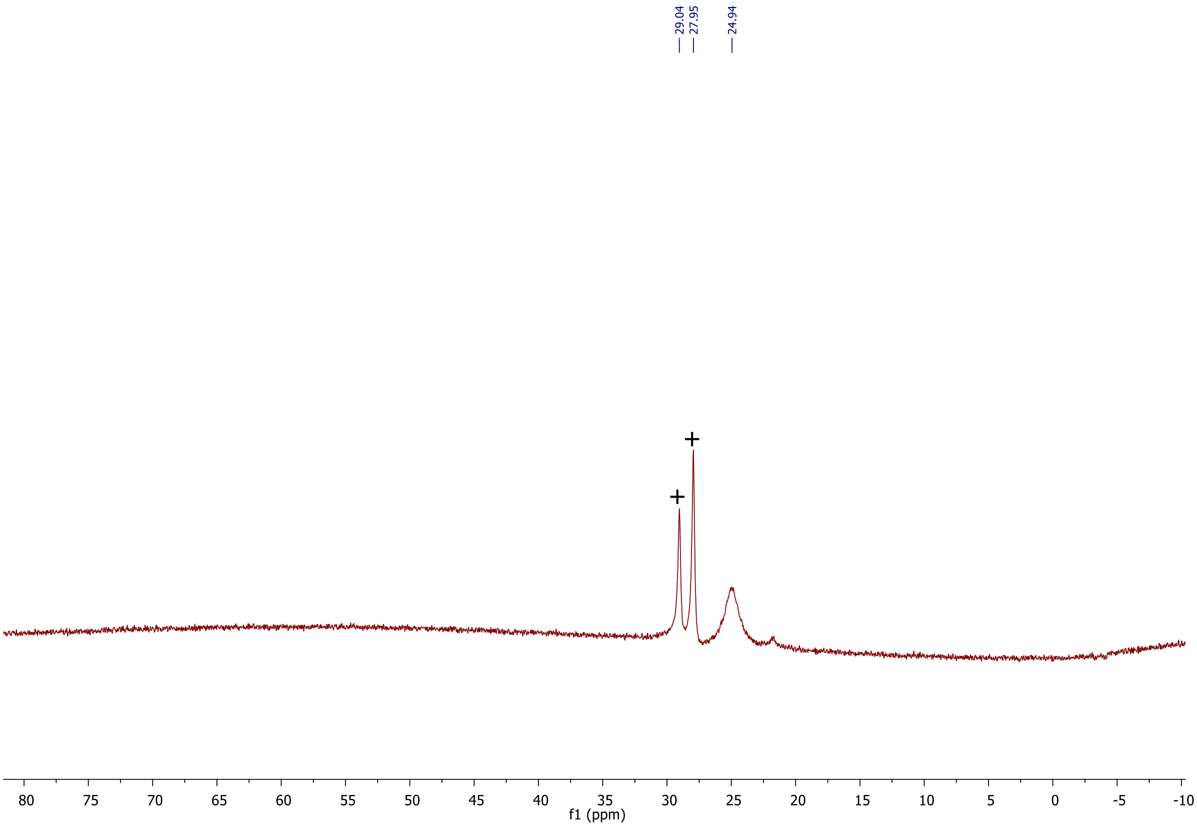

8n: ^{1}H-NMR (500 MHz, C_6D_6):

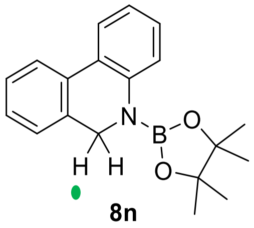

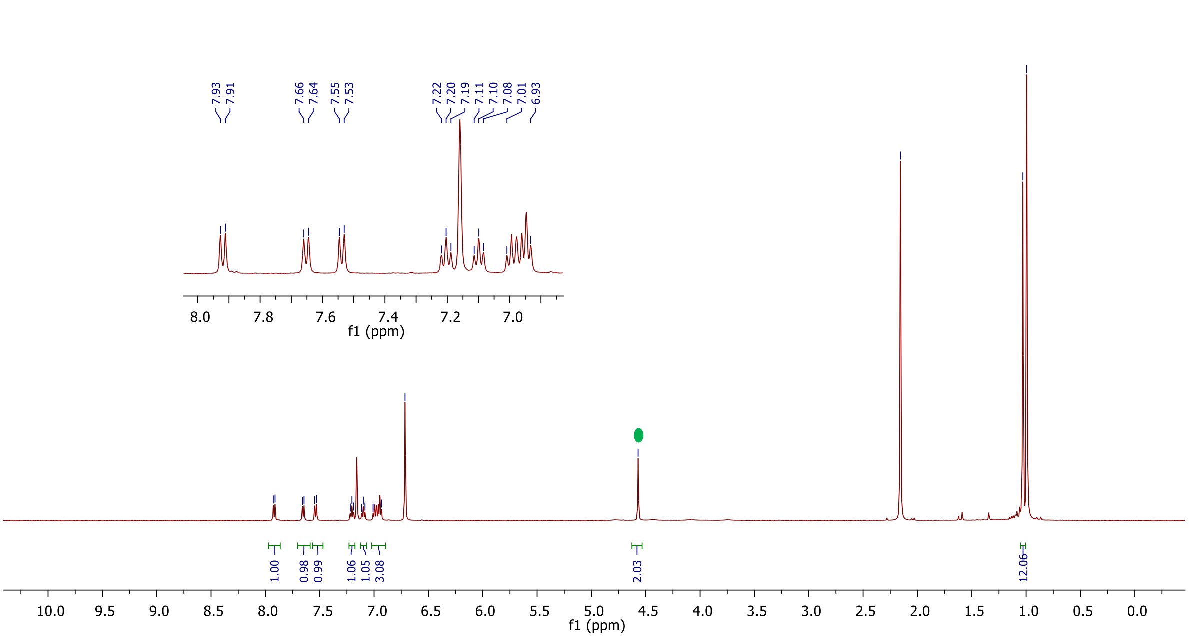

^{11}B-NMR (160 MHz, C_6D_6):

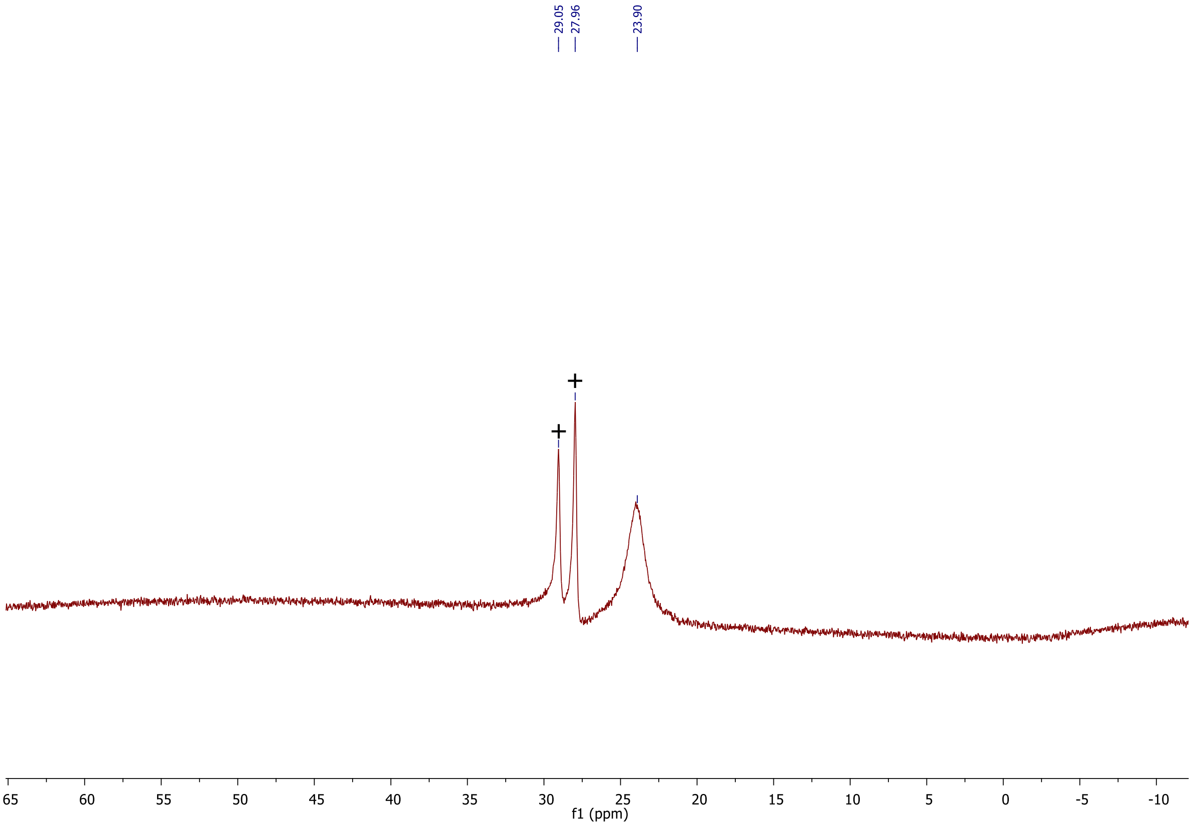

6 X-ray crystallographic data

6.1 Compound 2•THF (CCDC 2175816)

Table S2. Crystal data and structure refinement for 2

Empirical formula	$C_{43}H_{67}Br_2MnN_7OSi_2$	
Formula weight	968.97	
Temperature	150(2) K	
Wavelength	1.54184 Å	
Crystal system	Orthorhombic	
Space group	Pbca	
Unit cell dimensions	a = 16.52850(10) Å	a= 90°.
	b = 17.37800(10) Å	b= 90°.
	c = 34.2234(2) Å	g= 90°.
Volume	9830.06(10) Å^3	
Z	8	
Density (calculated)	1.309 Mg/m^3	
Absorption coefficient	4.848 mm^{-1}	
F(000)	4040	
Crystal size	0.270 x 0.120 x 0.080 mm^3	
Theta range for data collection	2.582 to 73.917°.	
Index ranges	-20<=h<=20, -21<=k<=21, -42<=l<=36	
Reflections collected	68256	
Independent reflections	9930 [R(int) = 0.0296]	
Completeness to theta = 67.684°	100.0 %	
Refinement method	Full-matrix least-squares on F^2	
Data / restraints / parameters	9930 / 0 / 519	
Goodness-of-fit on F^2	1.028	
Final R indices [I>2sigma(I)]	R1 = 0.0341, wR2 = 0.0838	
R indices (all data)	R1 = 0.0387, wR2 = 0.0878	
Largest diff. peak and hole	1.346 and -0.952 e.Å^{-3}	

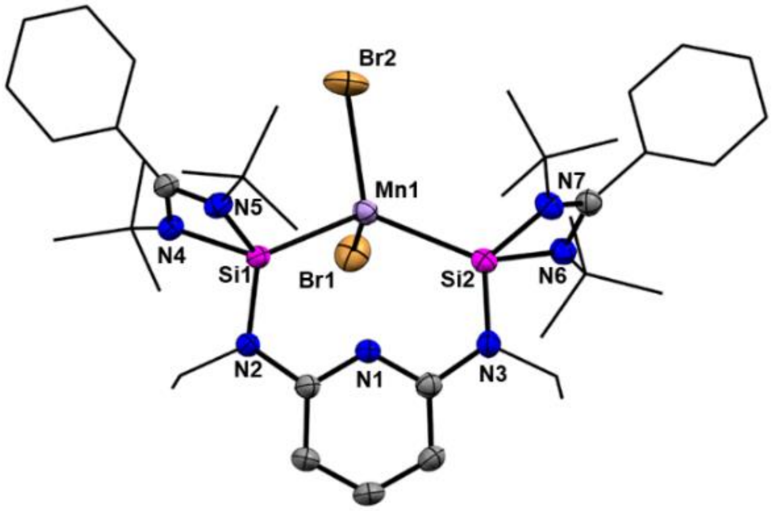

Figure S32: Molecular Structure of **2**. Thermal ellipsoids are drawn at 50% probability level. Hydrogen atoms are omitted for clarity.

Table S3. Bond lengths [Å] and angles [°] for 2.

Bond lengths [Å]		Bond angles [°]	
Br(1)-Mn(3)	2.4859(4)	Br(1)-Mn(1)-Br(2)	108.837(15)
Br(2)-Mn(3)	2.5054(4)	Br(1)-Mn(1)-Si(2)	106.940(19)
Mn(3)-Si(4)	2.5669(6)	Br(2)-Mn(1)-Si(2)	101.559(18)
Mn(3)-Si(5)	2.5996(7)	Br(1)-Mn(1)-Si(1)	106.492(18)
Si(4)-N(4)	1.7517(17)	Br(2)-Mn(1)-Si(1)	118.204(19)
Si(4)-N(1)	1.8378(18)	Si(2)-Mn(1)-Si(1)	114.28(2)
Si(4)-N(7)	1.8440(18)	N(4)-Si(2)-N(1)	105.20(8)

Compound 3 (CCDC 2175817)

Table S4. Crystal data and structure refinement for 3

Empirical formula	$C_{48}H_{82}MnN_7P_2Si_2$	
Formula weight	930.26	
Temperature	150(2) K	
Wavelength	1.54184 Å	
Crystal system	Monoclinic	
Space group	$P2_1/c$	
Unit cell dimensions	a = 13.5113(3) Å	a= 90°.
	b = 13.8328(3) Å	b= 92.402(2)°.
	c = 28.2254(5) Å	g= 90°.
Volume	5270.67(19) $Å^3$	
Z	4	
Density (calculated)	1.172 Mg/m^3	
Absorption coefficient	3.330 mm^{-1}	
F(000)	2008	
Crystal size	0.252 x 0.086 x 0.074 mm^3	
Theta range for data collection	3.134 to 67.496°.	
Index ranges	-16<=h<=16, -16<=k<=16, -33<=l<=27	
Reflections collected	35838	
Independent reflections	9507 [R(int) = 0.0515]	
Completeness to theta = 67.496°	100.0 %	
Absorption correction	Semi-empirical from equivalents	
Max. and min. transmission	1.00000 and 0.31548	
Refinement method	Full-matrix least-squares on F^2	
Data / restraints / parameters	9507 / 0 / 564	
Goodness-of-fit on F^2	1.024	
Final R indices [I>2sigma(I)]	R1 = 0.0445, wR2 = 0.1058	
R indices (all data)	R1 = 0.0654, wR2 = 0.1198	
Extinction coefficient	n/a	
Largest diff. peak and hole	0.531 and -0.354 e.$Å^{-3}$	

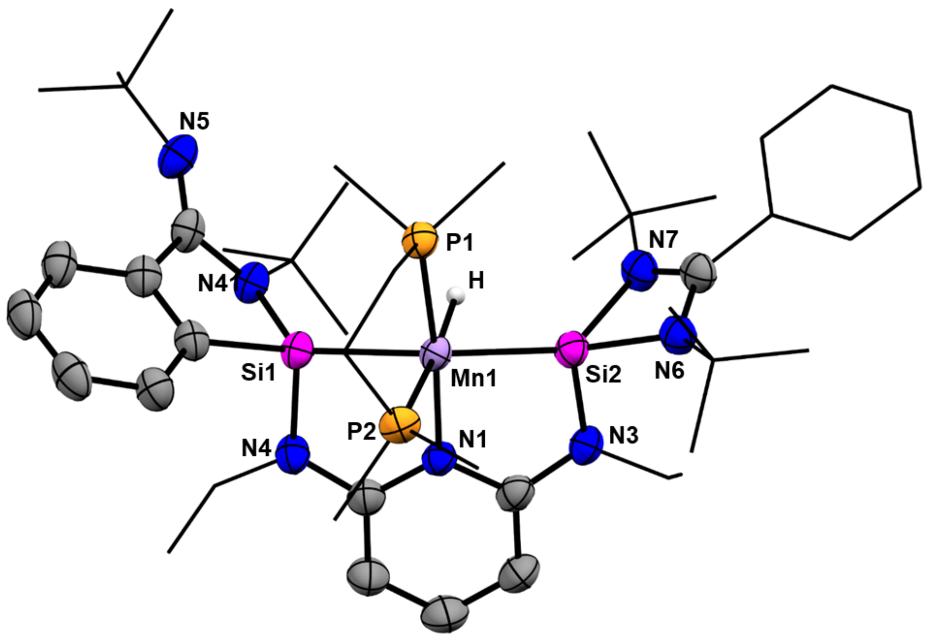

Figure S33: Molecular Structure of **3**. Thermal ellipsoids are drawn at 50% probability level. Hydrogen atoms are omitted for clarity except attached to Mn atom.

Table S5. Bond lengths [Å] and angles [°] for 3

**Bond lengths (Å)**		**Angles (°)**	
Mn(1)-N(1)	2.076(2)	N(1)-Mn(1)-Si(2)	82.04(6)
Mn(1)-Si(2)	2.2132(8)	N(1)-Mn(1)-P(1)	175.07(7)
Mn(1)-P(1)	2.2214(7)	Si(2)-Mn(1)-P(1)	102.32(3)
Mn(1)-P(2)	2.2531(8)	N(1)-Mn(1)-P(2)	95.55(6)
Mn(1)-Si(1)	2.3207(8)	Si(2)-Mn(1)-P(2)	116.61(3)
Si(2)-N(3)	1.776(2)	P(1)-Mn(1)-P(2)	84.59(3)
Si(2)-N(7)	1.888(2)	N(1)-Mn(1)-Si(1)	81.32(7)
Si(2)-N(6)	1.906(3)	Si(2)-Mn(1)-Si(1)	147.50(3)
Si(1)-N(2)	1.790(2)	P(1)-Mn(1)-Si(1)	93.75(3)
Si(1)-N(4)	1.813(2)	P(2)-Mn(1)-Si(1)	92.67(3)

Compound 4 (CCDC 2175818)

Table S6. Crystal data and structure refinement for 4

Empirical formula	$C_{45}H_{75}MnN_7P_2Si_2$	
Formula weight	887.18	
Temperature	150(2) K	
Wavelength	1.54184 Å	
Crystal system	Monoclinic	
Space group	$P2_1$	
Unit cell dimensions	a = 9.52200(10) Å	a= 90°.
	b = 18.7589(2) Å	b= 97.0220(10)°.
	c = 13.6576(2) Å	g= 90°.
Volume	2421.25(5) $Å^3$	
Z	2	
Density (calculated)	1.217 Mg/m^3	
Absorption coefficient	3.602 mm^{-1}	
F(000)	954	
Crystal size	0.280 x 0.170 x 0.090 mm^3	
Theta range for data collection	3.260 to 67.496°.	
Index ranges	-11<=h<=11, -22<=k<=22, -16<=l<=16	
Reflections collected	16262	
Independent reflections	8126 [R(int) = 0.0485]	
Completeness to theta = 67.496°	99.9 %	
Absorption correction	Semi-empirical from equivalents	
Max. and min. transmission	1.00000 and 0.11869	
Refinement method	Full-matrix least-squares on F^2	
Data / restraints / parameters	8126 / 1 / 533	
Goodness-of-fit on F^2	1.054	
Final R indices [I>2sigma(I)]	R1 = 0.0407, wR2 = 0.1048	
R indices (all data)	R1 = 0.0447, wR2 = 0.1093	
Absolute structure parameter	-0.014(5)	
Extinction coefficient	n/a	
Largest diff. peak and hole	1.158 and -0.392 e.$Å^{-3}$	

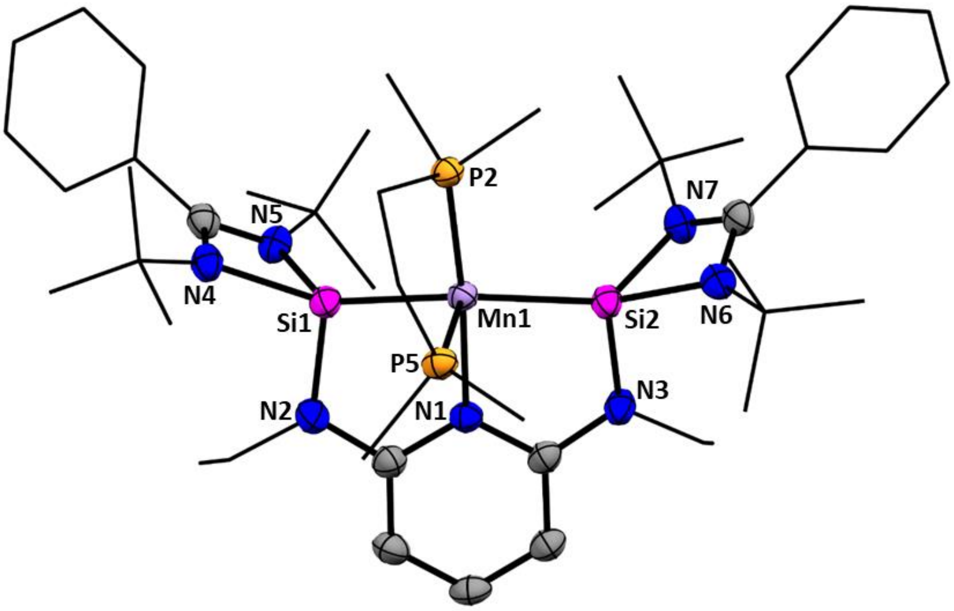

Figure S34: Molecular Structure of **4**. Thermal ellipsoids are drawn at 50% probability level. Hydrogen atoms are omitted for clarity.

Table S7. Bond lengths [Å] and angles [°] for 4

Bond lengths (Å)		Angles (°)	
Mn(1)-N(1)	2.111(3)	N(1)-Mn(1)-P(2)	175.36(11)
Mn(1)-P(2)	2.1765(11)	N(1)-Mn(1)-P(5)	93.38(10)
Mn(1)-P(5)	2.1901(13)	P(2)-Mn(1)-P(5)	86.11(5)
Mn(1)-Si(2)	2.2141(13)	N(1)-Mn(1)-Si(2)	79.44(10)
Mn(1)-Si(1)	2.2423(13)	P(2)-Mn(1)-Si(2)	96.04(5)
Si(1)-N(2)	1.770(4)	P(5)-Mn(1)-Si(2)	97.41(5)
Si(1)-N(4)	1.907(4)	N(1)-Mn(1)-Si(1)	80.27(10)
Si(1)-N(5)	1.926(4)	P(2)-Mn(1)-Si(1)	104.26(5)
Si(2)-N(3)	1.782(4)	P(5)-Mn(1)-Si(1)	109.00(5)
Si(2)-N(7)	1.914(4)	Si(2)-Mn(1)-Si(1)	147.43(5)
Si(2)-N(6)	1.921(4)	N(2)-Si(1)-N(4)	103.45(17)

Compound 5 (CCDC 2175819)

Table S8. Crystal data and structure refinement for 5•C_6H_6

Empirical formula	$C_{48}H_{65}MnN_7O_3Si_2$	
Formula weight	899.19	
Temperature	293(2) K	
Wavelength	1.54184 Å	
Crystal system	Monoclinic	
Space group	$P2_1$	
Unit cell dimensions	a = 10.1252(3) Å	a= 90°.
	b = 18.0882(5) Å	b= 103.606(3)°.
	c = 13.3966(5) Å	g = 90°.
Volume	2384.69(13) $Å^3$	
Z	2	
Density (calculated)	1.252 Mg/m^3	
Absorption coefficient	3.105 mm^{-1}	
F(000)	958	
Crystal size	0.260 x 0.190 x 0.130 mm^3	
Theta range for data collection	3.394 to 73.826°.	
Index ranges	-10<=h<=12, -22<=k<=16, -16<=l<=12	
Reflections collected	9625	
Independent reflections	6463 [R(int) = 0.0446]	
Completeness to theta = 67.684°	99.9 %	
Refinement method	Full-matrix least-squares on F^2	
Data / restraints / parameters	6463 / 1 / 564	
Goodness-of-fit on F^2	1.034	
Final R indices [I>2sigma(I)]	R1 = 0.0582, wR2 = 0.1444	
R indices (all data)	R1 = 0.0686, wR2 = 0.1577	
Absolute structure parameter	-0.017(8)	
Extinction coefficient	n/a	
Largest diff. peak and hole	0.736 and -0.627 e.$Å^{-3}$	

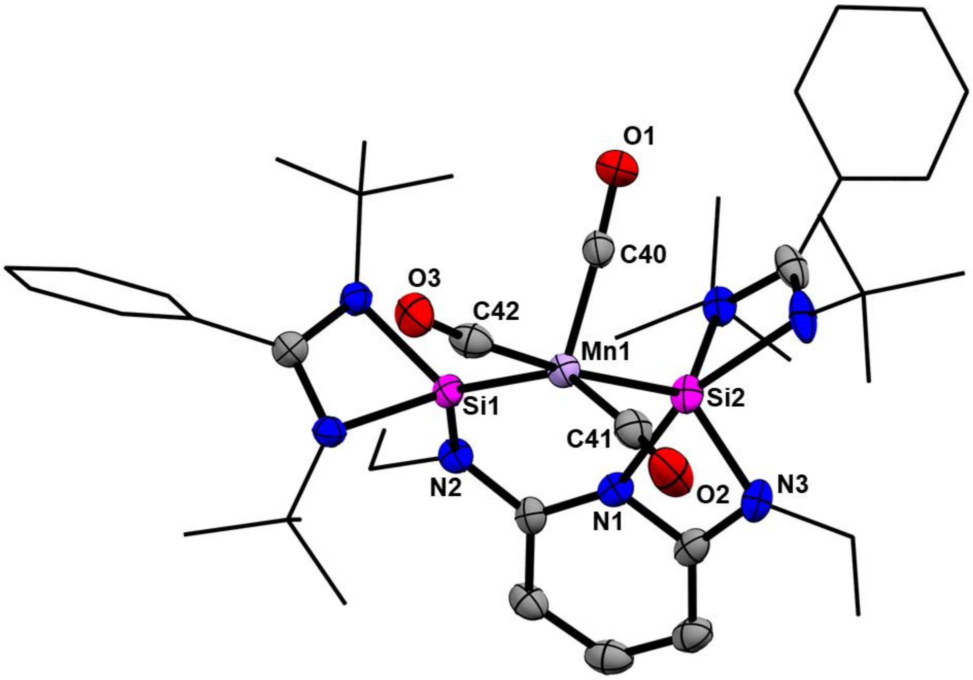

Figure S35: Molecular Structure of **5**. Thermal ellipsoids are drawn at 50% probability level. Hydrogen atoms are omitted for clarity.

Table S9. Bond lengths [Å] and angles [°] for 5

**Bond lengths (Å)**		**Angles (°)**	
Mn(1)-C(40)	1.776(7)	C(40)-Mn(1)-C(42)	90.8(3)
Mn(1)-C(42)	1.778(7)	C(40)-Mn(1)-C(41)	109.5(3)
Mn(1)-C(41)	1.801(7)	C(42)-Mn(1)-C(41)	92.9(3)
Mn(1)-Si(1)	2.2247(18)	C(40)-Mn(1)-Si(1)	106.4(2)
Mn(1)-Si(2)	2.3627(18)	C(42)-Mn(1)-Si(1)	85.9(2)
Si(1)-N(2)	1.781(5)	C(41)-Mn(1)-Si(1)	144.1(2)
Si(1)-N(7)	1.869(5)	C(40)-Mn(1)-Si(2)	83.8(2)
Si(1)-N(6)	1.874(6)	C(42)-Mn(1)-Si(2)	174.4(2)
Si(1)-C(6)	2.316(7)	C(41)-Mn(1)-Si(2)	90.6(2)
		Si(1)-Mn(1)-Si(2)	93.85(6)

7 Computational details

The DFT calculations were performed with Gaussian 16 (Revision A.03) program.[9] Geometry optimizations and frequency calculations were conducted at the PBE0[10]-D3BJ[11]/Def2-SVP[12]~ma-TZVP[13-14] level of theory in the gas phase. The ma-TZVP is the abbreviation of def2-TZVP with minimal augmentation, proposed by Truhlar and co-workers. All the principal interacting orbital (PIO)[15-16] and principal interacting spin orbitals (PISO)[17] analyses were performed by NBO 7.0 program[18] at the same level based on the optimized structure. All the orbitals were plotted with the help of Multiwfn[19] and VMD programs.[20] We can't locate the structure of $Mn(CO)_5$ in the quartet and sextet states by a full optimization as one of the CO ligands will dissociate during the optimization. The relative electronic energy of compound **$Mn(CO)_5$** in Table **S12** is obtained by a partial optimization with imaginary frequencies by fixing the Mn-C bonds at 1.836 and 1.837 Å in the quartet and sextet states, respectively, which are the average of Mn-C bonds in compounds **5** in the quartet and sextet states.

Table S10. The relative electronic energy (kcal mol^{-1}) of compound **4** in the three states.

State	**4**
doublet	0
quartet	3.2
sextet	4.5

Table S11. The relative electronic energy (kcal mol^{-1}) of compound **5** in the three states.

State	**5**
doublet	0
quartet	33.8
sextet	100.2

Table S12. The relative electronic energy (kcal mol^{-1}) of compound **$Mn(CO)_5$** in the two states.

State	**$Mn(CO)_5$**
doublet	0
quartet	54.3

sextet	130.7

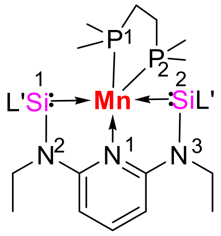

4

Table S13. Key distances (Å) and bond angles (°) of experimental and DFT-optimized structures of compound **4**.[a]

	Exp.	PBE0
Mn-Si1	2.214	2.194
Mn-Si2	2.242	2.211
Mn-N1	2.111	2.127
Mn-P1	2.177	2.182
Mn-P2	2.190	2.203
∠N1-Mn-Si1	79.4	79.1
∠Si1-Mn-P1	96.0	97.1
∠P1-Mn-P2	86.1	86.6
∠P2-Mn-Si2	109.0	107.3
∠N1-Mn-Si2	80.3	80.4
RD(%)[a]	0	0.8

[a] RD = $\frac{\sum_{i=1}^{n}\frac{|BL(DFT) - BL(Exp)|}{BL(Exp)} * 100\%}{n}$, BL means bond length.

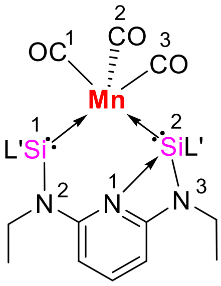

5

Table S14. Key distances (Å) and and bond angles (°) of experimental and DFT-optimized structures of compound **5**.[a]

	Exp.	PBE0
Mn-Si1	2.225	2.263
Mn-Si2	2.363	2.349
Mn-C1	1.778	1.764
Mn-C2	1.774	1.764
Mn-C3	1.803	1.799
N1-Si2	2.044	2.150
∠C1-Mn-Si1	85.9	83.4
∠C1-Mn-C2	90.8	93.3
∠C1-Mn-C3	93.0	89.8
∠C2-Mn-C3	109.5	109.4
∠C3-Mn-Si2	90.5	94.7
RD(%)[a]	0	1.8

[a] RD = $\frac{\sum_{i=1}^{n}\frac{|BL(DFT)-BL(Exp)|}{BL(Exp)}*100\%}{n}$, BL means bond length.

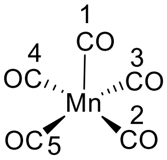

Table S15. Key distances (Å) and bond angles (°) of DFT-optimized structures of the compound **$Mn(CO)_5$** with C_{4v} symmetry.

	PBE0
Mn-C1	1.810
Mn-C2	1.849
∠C2-Mn-C3	89.3
∠C1-Mn-C2	96.3

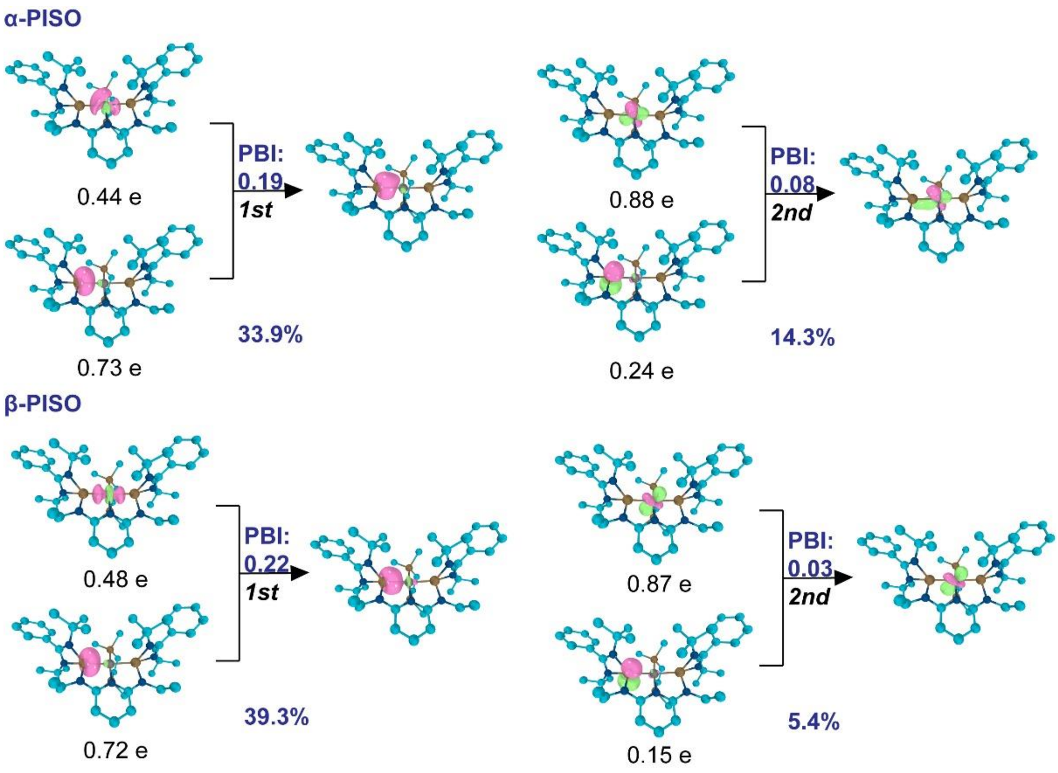

Figure S36: PISO analysis on the bonding modes of Mn-Si1 in the compound **4**. Hydrogen atoms in 3D structures are omitted for clarity. The PISO analysis is performed to the Mn and Si1 atom, rather than an entire molecule, in order to bring the interaction of interest to top. Each PISO pair results in a bonding PISMO (principal interacting spin molecular orbital). The PBI is used to quantify the strength of the interaction. The total PBI value of Mn-Si1 is 0.56, with the contribution of 0.29 from α system and 0.27 form β system. The isosurfaces with 0.080 au isovalue are plotted for the PISO pairs. Here 1st and 2nd mean the first and second PISO pairs, respectively.

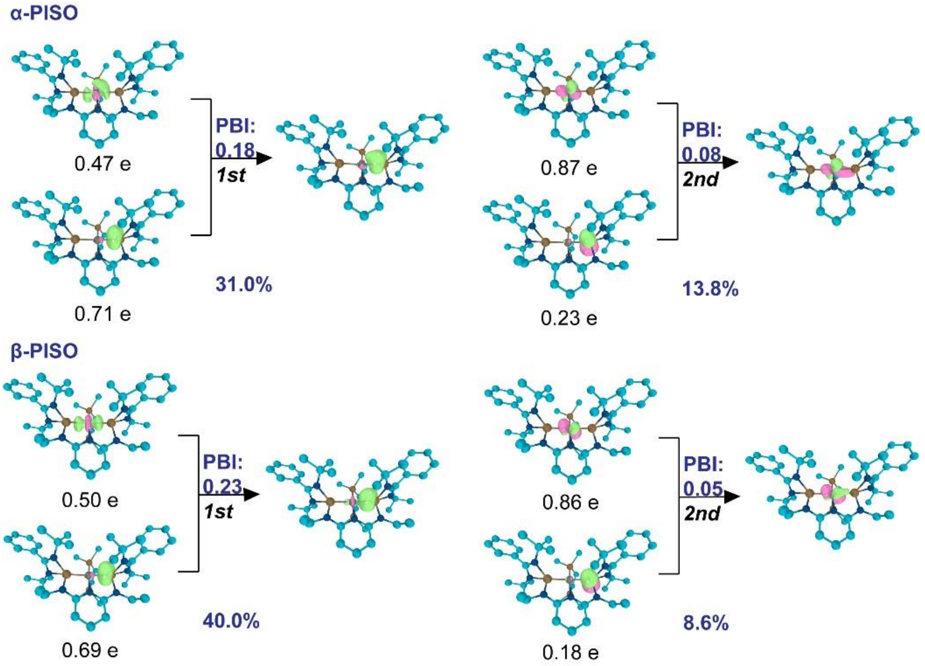

Figure S37: PISO analysis on the bonding modes of Mn-Si2 in the compound **4**. Hydrogen atoms in 3D structures are omitted for clarity. The PISO analysis is performed to the Mn and Si1 atom, rather than an entire molecule, in order to bring the interaction of interest to top. Each PISO pair results in a bonding PISMO (principal interacting spin molecular orbital). The PBI is used to quantify the strength of the interaction. The total PBI value of Mn-Si2 is 0.58, with the contribution of 0.28 from α system and 0.29 form β system. The isosurfaces with 0.080 au isovalue are plotted for the PISO pairs. Here 1st and 2nd mean the first and second PISO pairs, respectively.

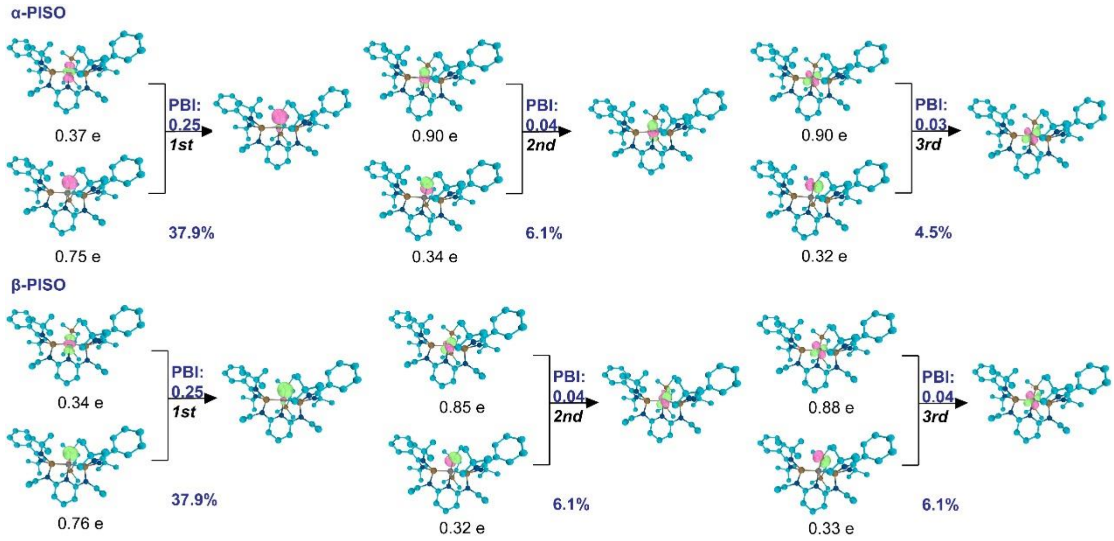

Figure S38: PISO analysis on the bonding modes of Mn-P1 in the compound **4**. Hydrogen atoms in 3D structures are omitted for clarity. The PISO analysis is performed to the Mn and P1 atom, rather than an entire molecule, in order to bring the interaction of interest to top.

Each PISO pair results in a bonding PISMO (principal interacting spin molecular orbital). The PBI is used to quantify the strength of the interaction. The total PBI value of Mn-P1 is 0.66, with the contribution of 0.33 from α system and 0.33 form β system. The isosurfaces with 0.080 au isovalue are plotted for the PISO pairs. Here 1st, 2nd and 3rd mean the first, second and third PISO pairs, respectively.

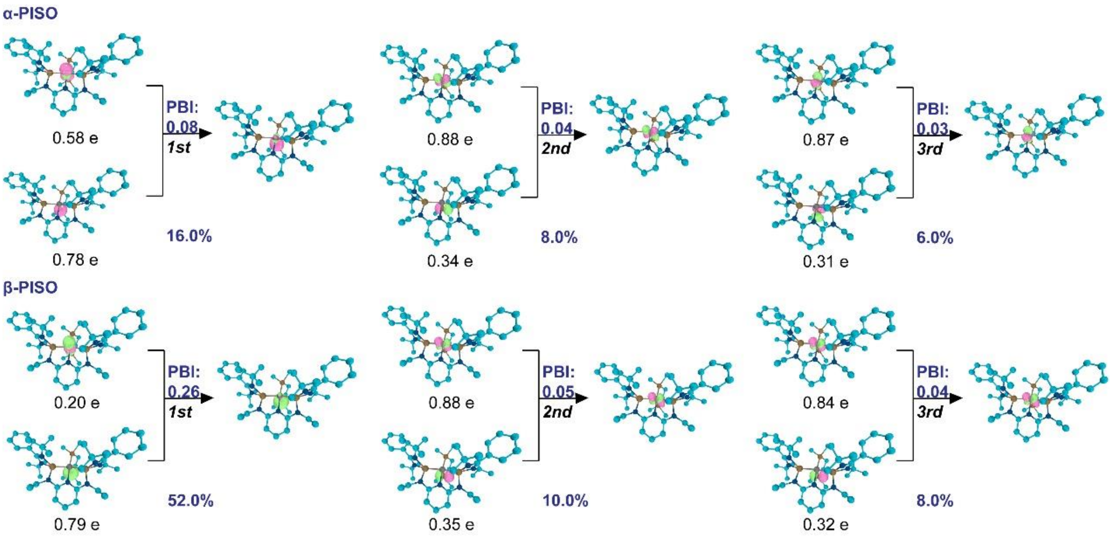

Figure S39: PISO analysis on the bonding modes of Mn-P2 in the compound **4**. Hydrogen atoms in 3D structures are omitted for clarity. The PISO analysis is performed to the Mn and P2 atom, rather than an entire molecule, in order to bring the interaction of interest to top. Each PISO pair results in a bonding PISMO (principal interacting spin molecular orbital). The PBI is used to quantify the strength of the interaction. The total PBI value of Mn-P2 is 0.50, with the contribution of 0.16 from α system and 0.34 form β system. The isosurfaces with 0.080 au isovalue are plotted for the PISO pairs. Here 1st, 2nd and 3rd mean the first, second and third PISO pairs, respectively.

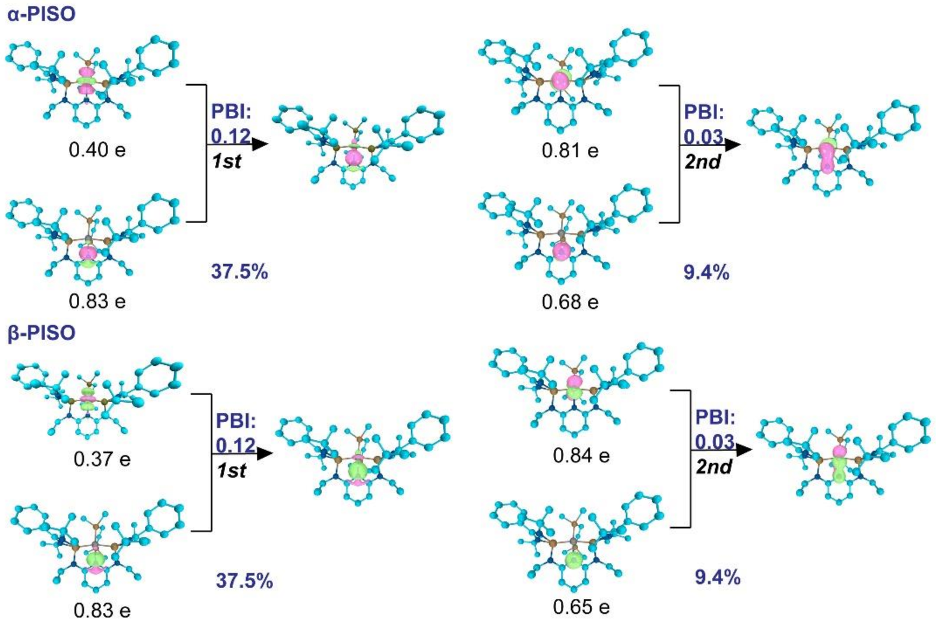

Figure S40: PISO analysis on the bonding modes of Mn-N1 in the compound **4**. Hydrogen atoms in 3D structures are omitted for clarity. The PISO analysis is performed to the Mn and N1 atom, rather than an entire molecule, in order to bring the interaction of interest to top. Each PISO pair results in a bonding PISMO (principal interacting spin molecular orbital). The PBI is used to quantify the strength of the interaction. The total PBI value of Mn-N1 is 0.32, with the contribution of 0.16 from α system and 0.16 form β system. The isosurfaces with 0.080 au isovalue are plotted for the PISO pairs. Here 1st and 2nd mean the first and second PISO pairs, respectively.

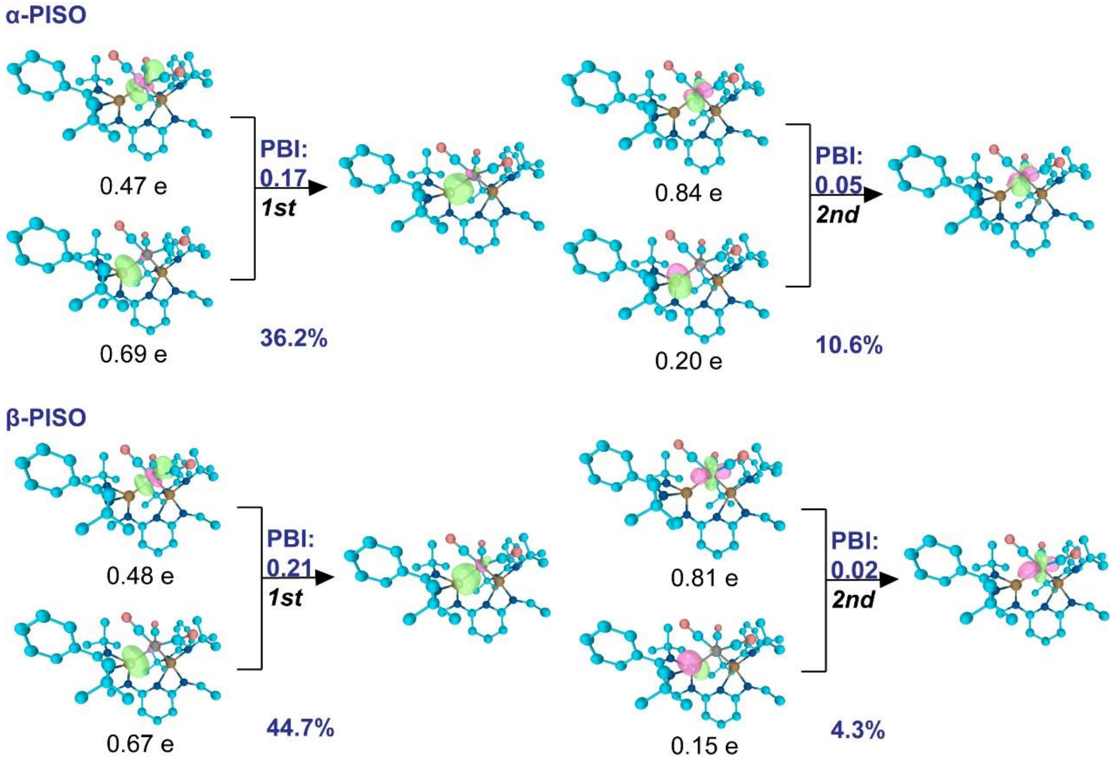

Figure S41: PISO analysis on the bonding modes of Mn-Si1 in the compound **5**. Hydrogen atoms in 3D structures are omitted for clarity. The PISO analysis is performed to the Mn and Si1 atom, rather than an entire molecule, in order to bring the interaction of interest to top. Each PISO pair results in a bonding PISMO (principal interacting spin molecular orbital). The PBI is used to quantify the strength of the interaction. The total PBI value of Mn-Si1 is 0.47, with the contribution of 0.23 from α system and 0.24 form β system. The isosurfaces with 0.080 au isovalue are plotted for the PISO pairs. Here 1st and 2nd mean the first and second PISO pairs, respectively.

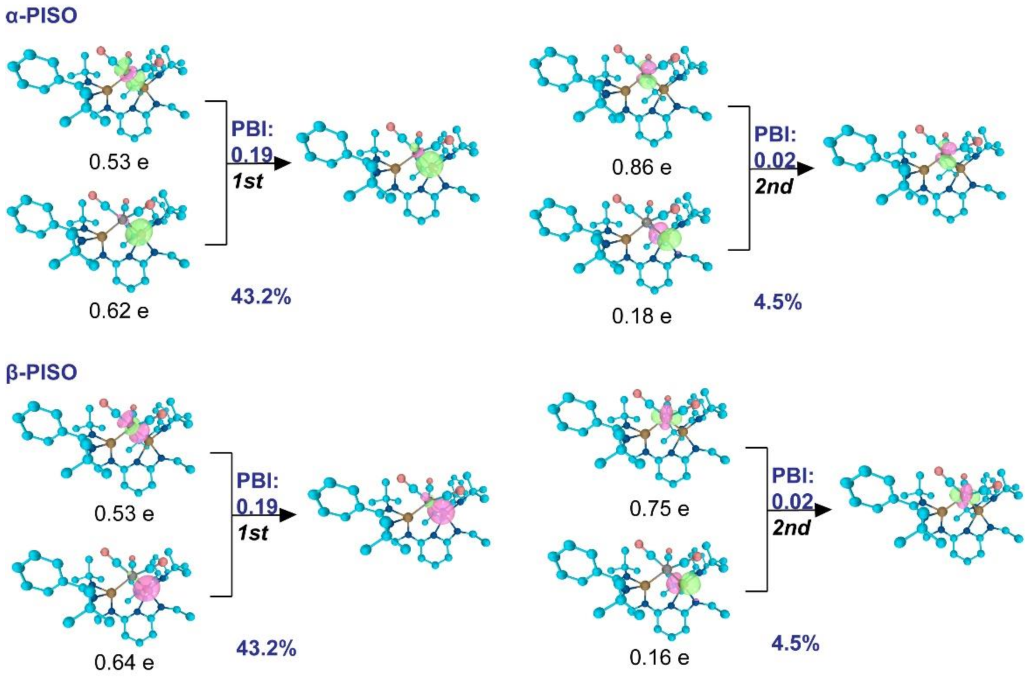

Figure S42: PISO analysis on the bonding modes of Mn-Si2 in the compound **5**. Hydrogen atoms in 3D structures are omitted for clarity. The PISO analysis is performed to the Mn and Si2 atom, rather than an entire molecule, in order to bring the interaction of interest to top. Each PISO pair results in a bonding PISMO (principal interacting spin molecular orbital). The PBI is used to quantify the strength of the interaction. The total PBI value of Mn-Si2 is 0.44, with the contribution of 0.22 from α system and 0.22 form β system. The isosurfaces with 0.080 au isovalue are plotted for the PISO pairs. Here 1st and 2nd mean the first and second PISO pairs, respectively.

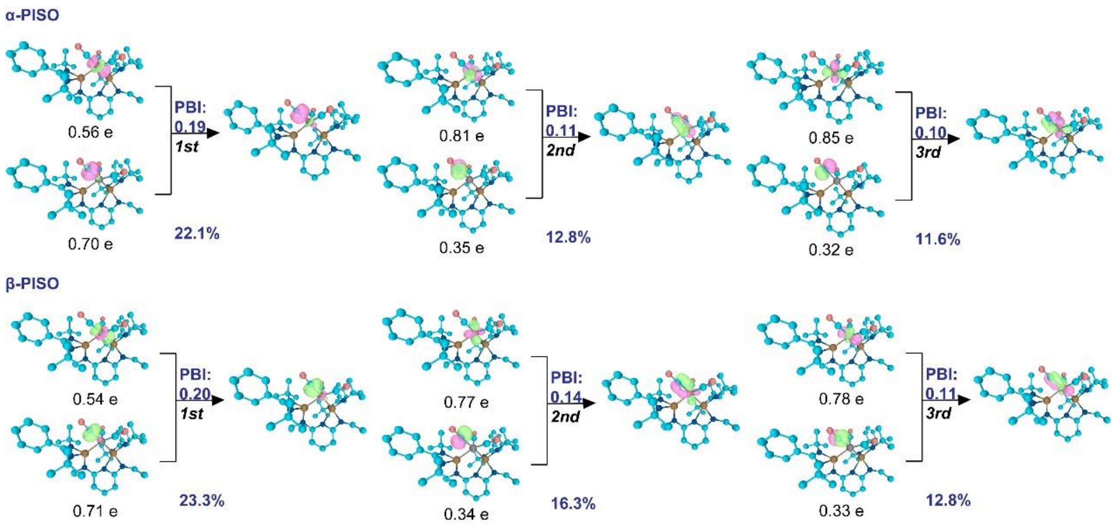

Figure S43: PISO analysis on the bonding modes of Mn-C1 in the compound **5**. Hydrogen atoms in 3D structures are omitted for clarity. The PISO analysis is performed to the Mn and C1 atom, rather than an entire molecule, in order to bring the interaction of interest to top. Each PISO pair results in a bonding PISMO (principal interacting spin molecular orbital). The PBI is used to quantify the strength of the interaction. The total PBI value of Mn-C1 is 0.86, with the contribution of 0.41 from α system and 0.45 form β system. The isosurfaces with 0.080 au isovalue are plotted for the PISO pairs. Here 1st, 2nd and 3rd mean the first, second and third PISO pairs, respectively.

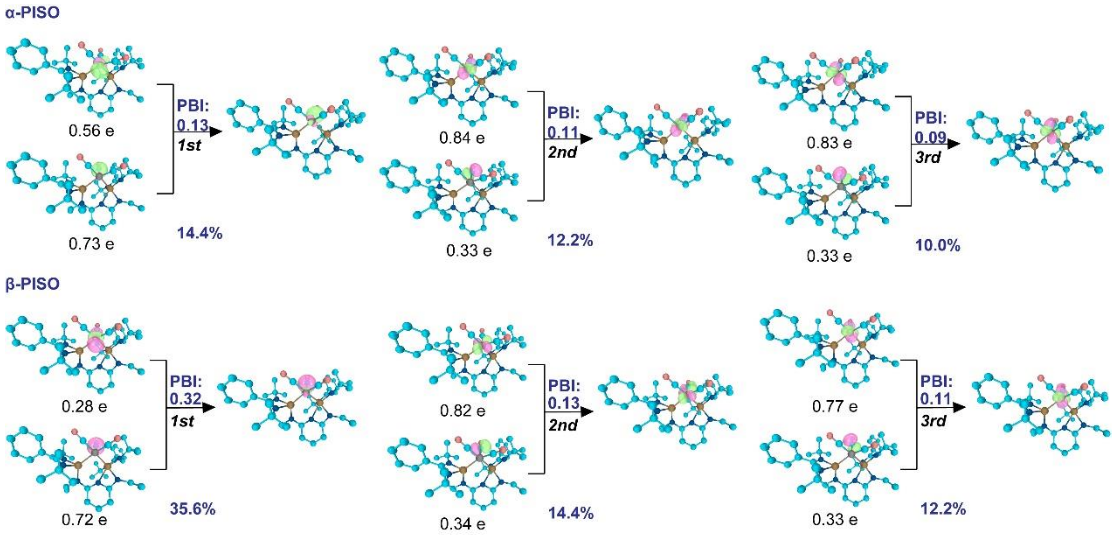

Figure S44: PISO analysis on the bonding modes of Mn-C2 in the compound **5**. Hydrogen atoms in 3D structures are omitted for clarity. The PISO analysis is performed to the Mn and C2 atom, rather than an entire molecule, in order to bring the interaction of interest to top. Each PISO pair results in a bonding PISMO (principal interacting spin molecular orbital). The PBI is used to quantify the strength of the interaction. The total PBI value of Mn-C2 is 0.86, with the contribution of 0.34 from α system and 0.52 form β system. The isosurfaces with

0.080 au isovalue are plotted for the PISO pairs. Here 1st, 2nd and 3rd mean the first, second and third PISO pairs, respectively.

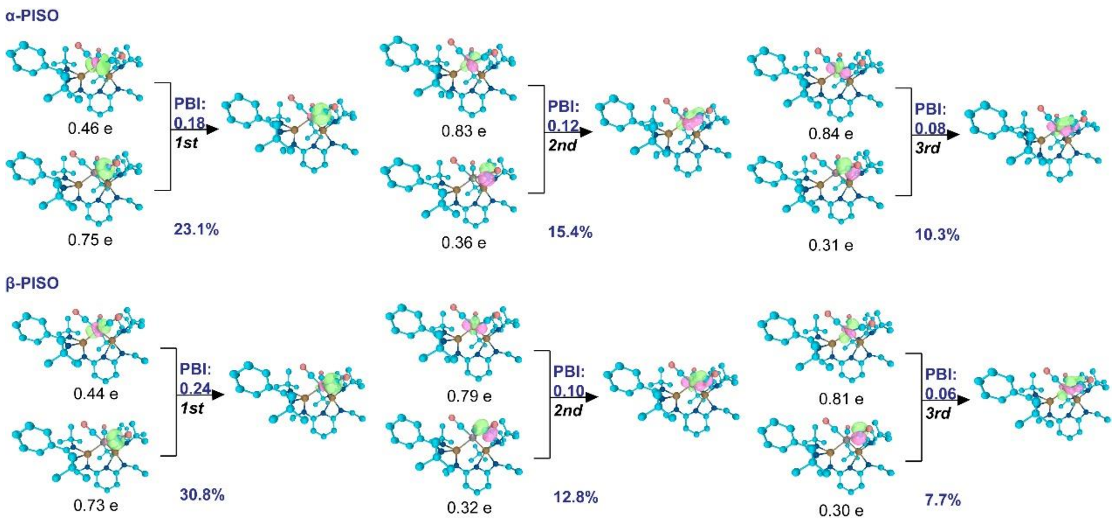

Figure S45: PISO analysis on the bonding modes of Mn-C3 in the compound **5**. Hydrogen atoms in 3D structures are omitted for clarity. The PISO analysis is performed to the Mn and C3 atom, rather than an entire molecule, in order to bring the interaction of interest to top. Each PISO pair results in a bonding PISMO (principal interacting spin molecular orbital). The PBI is used to quantify the strength of the interaction. The total PBI value of Mn-C3 is 0.78, with the contribution of 0.39 from α system and 0.39 form β system. The isosurfaces with 0.080 au isovalue are plotted for the PISO pairs. Here 1st, 2nd and 3rd mean the first, second and third PISO pairs, respectively.

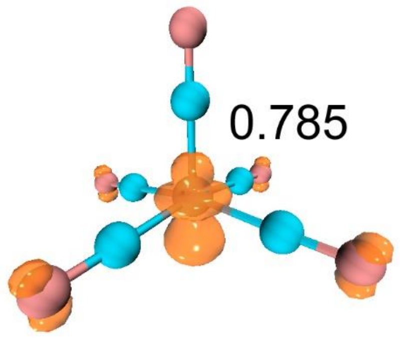

Figure S46: The spin density population of $Mn(CO)_5$ with 0.010 au isovalue.

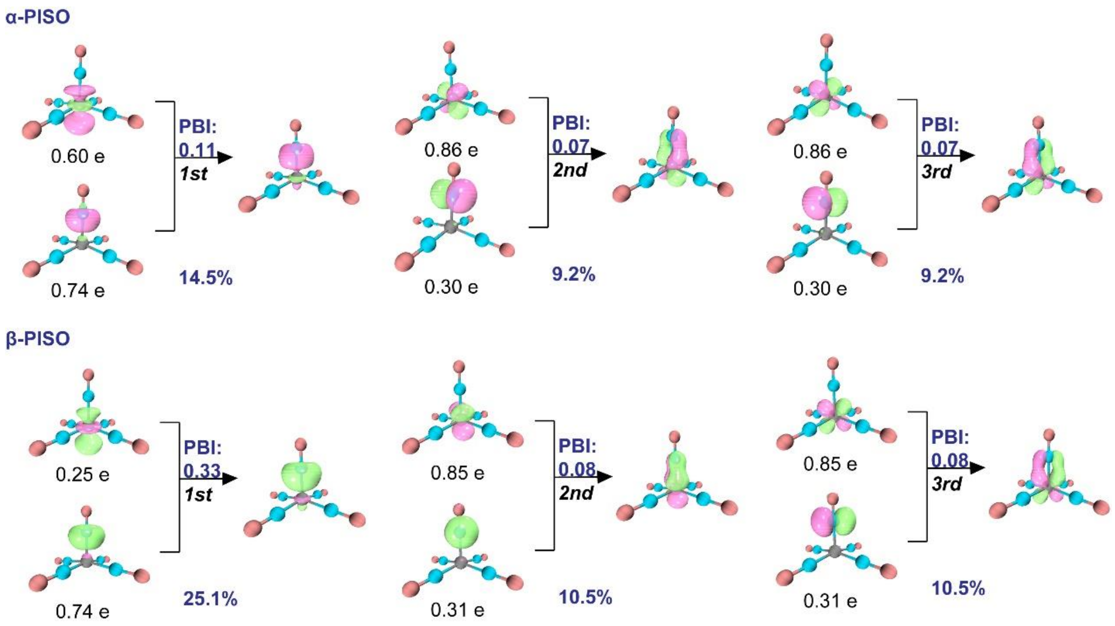

Figure S47: PISO analysis on the bonding modes of Mn-C1 in the compound $Mn(CO)_5$. The PISO analysis is performed to the Mn and C1 atom, rather than an entire molecule, in order to bring the interaction of interest to top. Each PISO pair results in a bonding PISMO (principal interacting spin molecular orbital). The PBI is used to quantify the strength of the interaction. The total PBI value of Mn-C1 is 0.76, with the contribution of 0.26 from α system and 0.50 form β system. The isosurfaces with 0.080 au isovalue are plotted for the PISO pairs. Here 1st, 2nd and 3rd mean the first, second and third PISO pairs, respectively.

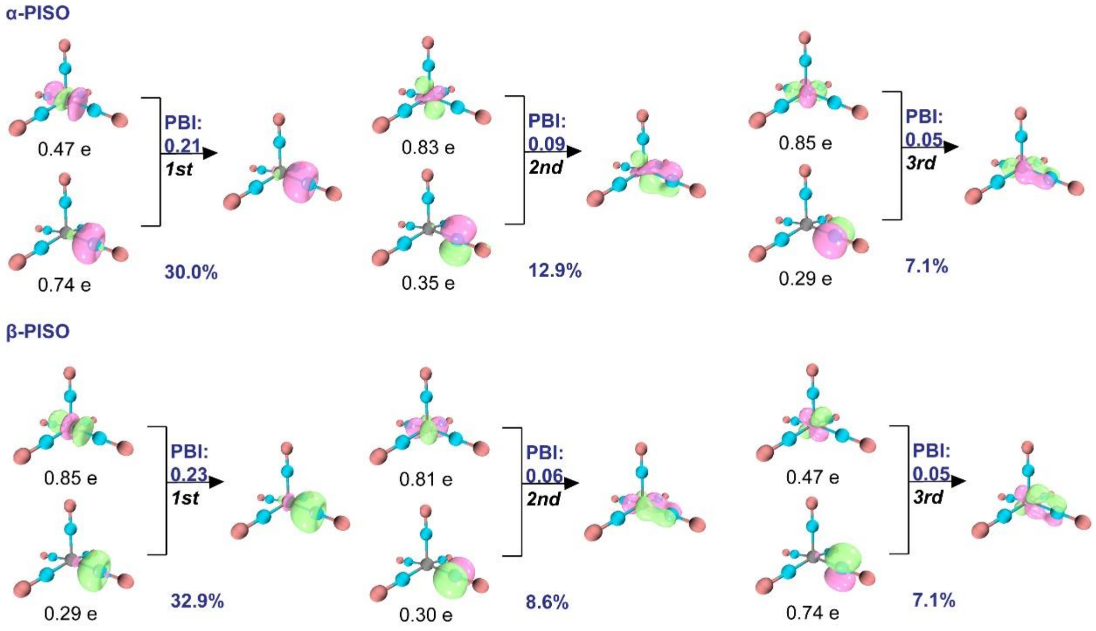

Figure S48: PISO analysis on the bonding modes of Mn-C2 in the compound $Mn(CO)_5$. The PISO analysis is performed to the Mn and C2 atom , rather than an entire molecule, in order to bring the interaction of interest to top. Each PISO pair results in a bonding PISMO (principal

interacting spin molecular orbital). The PBI is used to quantify the strength of the interaction. The total PBI value of Mn-C2 is 0.70, with the contribution of 0.36 from α system and 0.34 form β system. The isosurfaces with 0.080 au isovalue are plotted for the PISO pairs. Here 1st, 2nd and 3rd mean the first, second and third PISO pairs, respectively.

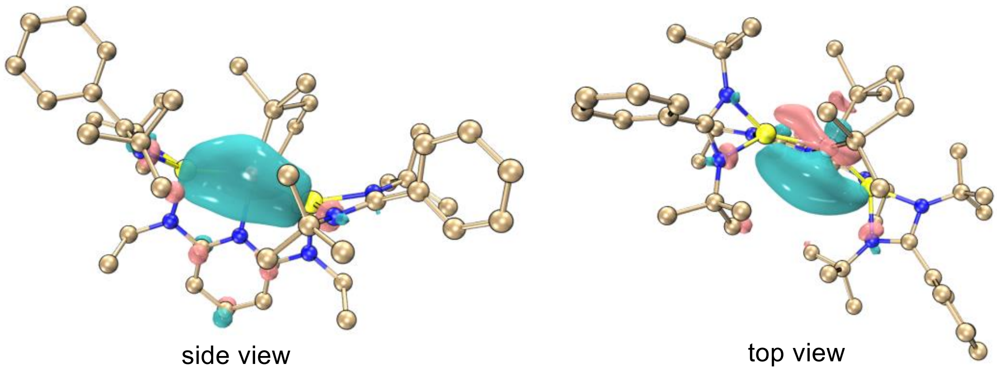

side view
-2.96 eV

top view

Figure S49: The highest singly occupied molecular orbital (HSOMO) of the compound **4**. The isosurfaces with 0.080 au isovalue are plotted for the orbital.

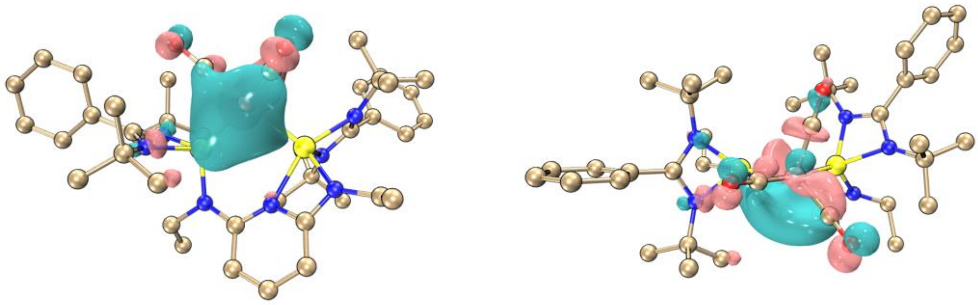

side view
-4.51 eV

top view

Figure S50: The highest singly occupied molecular orbital (HSOMO) of the compound **5**. The isosurfaces with 0.080 au isovalue are plotted for the orbital.

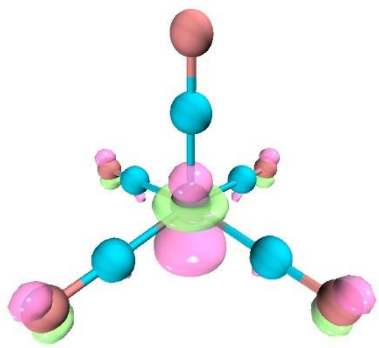

Figure S51: The highest singly occupied molecular orbital (HSOMO) of the compound **$Mn(CO)_5$**. The isosurfaces with 0.080 au isovalue are plotted for the orbital.

Cartesian Coordinates

4.doublet

PBE0-D3BJ/Def2-SVP~ma-TZVP

E = -4551.120168 a.u.

Mn	-0.05891000	-0.63115000	-0.45035700
P	-0.01105400	0.64104200	-2.22302900
Si	-2.12232800	-0.43681100	0.32086500
Si	2.08659400	-0.54835200	0.00275500
P	-0.03817400	-2.34191400	-1.83858800
N	0.05372200	-1.96599500	1.20144200
N	-2.21513600	-1.62497600	1.66290300
N	-3.96530300	-0.39760200	-0.26992500
N	3.83245100	-0.18038900	-0.75136900
N	2.38000700	-2.03587700	0.97539700
N	-3.10354100	1.08407100	1.02190100
N	3.08798300	0.74676500	1.03689200
C	3.67687100	-2.56260700	1.33661900
H	3.66927000	-3.66468700	1.25805000
H	4.38880000	-2.21699900	0.57345500
C	1.82292400	2.78004600	1.44655100
H	2.44320900	3.38929900	0.77191600
H	1.39941800	3.44433800	2.21523200
H	1.00235300	2.33649600	0.86346300
C	-5.26237900	1.73732300	-0.08488500
C	4.62610600	0.49982300	-2.99769600
H	3.59480500	0.73180200	-3.29760200
H	5.17087000	0.14818200	-3.88722600
H	5.10465500	1.42811700	-2.65708000

C	-4.83796900	-1.25002700	-1.07659100
C	7.31140000	2.58894900	0.80433400
H	8.23743900	2.42102900	1.35889300
C	0.25544700	-1.76352100	-3.61207000
H	0.94261000	-2.44637200	-4.13916200
H	-0.71921700	-1.84901800	-4.12064800
C	-3.37615600	-1.83359100	2.49991400
H	-3.65065900	-2.90476200	2.52068200
H	-4.22026300	-1.32584300	2.01063200
C	4.04900000	0.79945600	0.13575000
C	-1.46703100	-3.48141200	-2.18627000
H	-1.22035000	-4.22385500	-2.96344000
H	-2.34284900	-2.90654600	-2.50890600
H	-1.72470800	-4.01661300	-1.25995600
C	-1.53509500	1.31990600	-3.03952500
H	-2.02811900	2.00830400	-2.33702800
H	-2.22783800	0.49320500	-3.24373700
H	-1.31886500	1.85600900	-3.97773300
C	1.02505700	2.17820500	-2.32383300
H	1.09061100	2.57976600	-3.34844600
H	2.03436800	1.94036600	-1.95802000
H	0.59809900	2.94958100	-1.66562100
C	-4.48998800	-1.06530000	-2.55605800
H	-4.72541800	-0.04139900	-2.88218700
H	-5.05896000	-1.76714300	-3.18477800
H	-3.41735000	-1.23622900	-2.71771900
C	-4.56448600	-2.69204000	-0.63897400
H	-3.48784300	-2.89430600	-0.60079200

H	-5.02904700	-3.40261500	-1.33873700
H	-4.97874100	-2.87493400	0.36253800
C	-7.36524400	3.45060100	-0.75312300
H	-8.18891000	4.11897500	-1.01452700
C	-1.06283700	-3.45471700	2.75637400
H	-1.97920200	-3.76954400	3.25052300
C	4.04343800	-1.86344500	-2.44934700
H	4.14033400	-2.67873000	-1.71835500
H	4.55843600	-2.16778000	-3.37204200
H	2.97662100	-1.73349400	-2.67223300
C	0.73251600	-0.31926600	-3.65067100
H	0.51724100	0.16513700	-4.61841600
H	1.82036700	-0.26656500	-3.49867000
C	4.64789400	-0.57351400	-1.90395000
C	-1.06919000	-2.35221700	1.88107500
C	-7.38095600	2.76462600	0.46185800
H	-8.21712800	2.89402900	1.15282200
C	-4.14608900	0.81505500	0.24258300
C	-6.33178700	1.91482300	0.79809400
H	-6.33905800	1.37744600	1.74878500
C	2.65799500	1.66605600	2.08575400
C	4.94219500	3.01413900	-0.60304400
H	4.01078700	3.17913500	-1.14944900
C	1.23995500	-2.57449400	1.52188200
C	-6.32837000	-0.97287200	-0.86736000
H	-6.58719600	-0.98293200	0.20213000
H	-6.90513400	-1.76987200	-1.36066900
H	-6.65100700	-0.01470300	-1.29400300

C	0.12277900	-4.13644700	2.97293000
H	0.14231200	-5.00679200	3.63366900
C	6.32155100	1.61048300	0.80031100
H	6.46334200	0.67977200	1.35417400
C	6.09873500	-0.85766000	-1.49830400
H	6.63297100	0.05568000	-1.20648800
H	6.63955500	-1.30330700	-2.34696000
H	6.13837800	-1.56968200	-0.65994900
C	5.93406400	3.99270600	-0.59501400
H	5.77794300	4.92603600	-1.14075100
C	-1.51848900	1.99593800	2.54338200
H	-1.93059300	1.45379100	3.40591800
H	-1.01034200	2.89995800	2.90868400
H	-0.77904300	1.34676400	2.05208200
C	1.29242300	-3.68950700	2.37719900
H	2.23950800	-4.18229700	2.58392800
C	-2.01633600	3.19389400	0.41393000
H	-1.23869700	2.60533900	-0.09603500
H	-1.56471600	4.11912100	0.80406900
H	-2.78755100	3.47435800	-0.31961700
C	1.15884800	-3.74616500	-1.60795300
H	0.84396500	-4.29583300	-0.70710800
H	2.17287700	-3.37960400	-1.42505600
H	1.15659100	-4.44120300	-2.46264100
C	5.13171200	1.81694400	0.09464000
C	1.79021000	0.83140900	3.03188400
H	1.00932700	0.29925700	2.46614000
H	1.30649300	1.47502300	3.78068100

H	2.39833400	0.08104800	3.55613100
C	-2.61800300	2.35980500	1.54964400
C	-6.29691400	3.28168200	-1.63259200
H	-6.27917400	3.81813200	-2.58407500
C	3.82054800	2.26578900	2.87736400
H	4.49350700	1.47833900	3.24930300
H	3.41725600	2.80402700	3.74843900
H	4.41141600	2.97893200	2.28755200
C	-3.70200700	3.15608600	2.27690500
H	-4.45000000	3.57525400	1.59170200
H	-3.23261800	3.99476000	2.81333200
H	-4.21763800	2.52570400	3.01730900
C	-5.24951300	2.42370300	-1.30321900
H	-4.41447400	2.28038200	-1.99296200
C	7.11936900	3.78218700	0.10781400
H	7.89577200	4.55066100	0.11425800
C	-3.23508300	-1.30034600	3.91857700
H	-2.37592800	-1.74924400	4.43825100
H	-4.14143000	-1.51488000	4.50667200
H	-3.08815100	-0.21150500	3.89981100
C	4.18945800	-2.13500400	2.70679000
H	4.31075500	-1.04302700	2.74204500
H	5.16568200	-2.59929200	2.91938900
H	3.49265900	-2.42269600	3.50793400

4.quartet

PBE0-D3BJ/Def2-SVP~ma-TZVP

E = -4551.114996 a.u.

Mn	-0.09039200	-0.81070900	-0.31977300

P	-0.43138700	-0.25701100	-2.44879900
Si	-2.17275100	-0.06718900	0.43086400
Si	2.14964900	-0.47243600	-0.01619000
P	0.10294300	-2.93907600	-1.20092000
N	0.13170600	-1.37564900	1.65650200
N	-2.00627800	-0.57015800	2.16531900
N	-3.87896800	-0.46510700	-0.05945000
N	3.90516300	-0.32152300	-0.75592500
N	2.39689700	-1.72636800	1.26607300
N	-3.11947900	1.49856300	0.52042700
N	3.10804400	1.01651700	0.72425600
C	3.67335800	-2.31356000	1.61975600
H	3.52313800	-3.36687500	1.91293600
H	4.28692900	-2.34070000	0.70823600
C	2.29566400	3.31293800	0.92529300
H	3.11394400	3.78191500	0.36028900
H	1.89624600	4.06085200	1.62662500
H	1.49590300	3.03549700	0.22630700
C	-5.47618700	1.45504700	-0.46041100
C	4.27780000	0.08896100	-3.15762500
H	3.19306900	0.20885400	-3.29332000
H	4.69626100	-0.33565800	-4.08277300
H	4.72180900	1.08399500	-3.01494300
C	-4.70984100	-1.66710600	0.04098100
C	7.54276900	2.37493100	0.27075800
H	8.46938500	2.17976200	0.81505600
C	0.28555800	-2.87018400	-3.07068400
H	1.10675500	-3.53148500	-3.39117400

H	-0.63900300	-3.29999700	-3.48798500
C	-3.01247800	-0.21490000	3.14678400
H	-3.36599600	-1.10637400	3.69652300
H	-3.88258600	0.16100200	2.58962700
C	4.11867500	0.83274900	-0.10257300
C	-1.32770700	-4.10079800	-1.06687400
H	-1.14117600	-5.03139400	-1.62653700
H	-2.23669400	-3.62044300	-1.45035900
H	-1.49430100	-4.35141500	-0.00946400
C	-2.11407900	-0.28684800	-3.19493400
H	-2.78323500	0.38705300	-2.64196400
H	-2.52618500	-1.30004600	-3.09070400
H	-2.09849400	-0.00614900	-4.25946300
C	0.20421900	1.35753200	-3.07272900
H	0.11974500	1.44312200	-4.16754100
H	1.25758300	1.45832600	-2.77323000
H	-0.36005300	2.17648700	-2.60520200
C	-4.91885500	-2.30109100	-1.34153800
H	-5.48383600	-1.63667000	-2.00690300
H	-5.47444300	-3.24834800	-1.25884500
H	-3.94825000	-2.51081000	-1.81362800
C	-3.97167000	-2.68309800	0.92039900
H	-2.92413300	-2.78543500	0.60727100
H	-4.44859600	-3.67210200	0.84692600
H	-3.97751000	-2.37809800	1.97457100
C	-7.92220500	2.59426000	-1.39786200
H	-8.85662700	3.03075400	-1.75602800
C	-0.87062300	-1.99537200	3.76175000

H	-1.72399000	-1.97183100	4.43525300
C	3.98971200	-2.20894600	-2.23324600
H	4.27492200	-2.91611600	-1.44087800
H	4.36145100	-2.60189800	-3.19034800
H	2.89326300	-2.16444800	-2.27798300
C	0.47098400	-1.44243300	-3.57039700
H	0.14875800	-1.33087000	-4.61912500
H	1.52932500	-1.14617000	-3.52170100
C	4.57050800	-0.82446200	-1.96305200
C	-0.92160200	-1.30885600	2.52937700
C	-7.33989900	3.02711800	-0.19702500
H	-7.84036700	3.79268400	0.40333900
C	-4.26475100	0.86847100	0.00494500
C	-6.15455500	2.47972400	0.26390200
H	-5.76680000	2.78052600	1.23714400
C	2.79018700	2.07687900	1.68174200
C	5.17021800	2.87335000	-1.10803300
H	4.23864800	3.06913500	-1.64339000
C	1.30276900	-1.95019200	2.06351200
C	-6.06957100	-1.38329200	0.68621500
H	-5.94122500	-0.86635000	1.64881500
H	-6.58836000	-2.33647300	0.87340000
H	-6.71265700	-0.76041500	0.05195100
C	0.26597700	-2.71281000	4.08716500
H	0.30238100	-3.27477700	5.02412800
C	6.46578400	1.50449000	0.40665200
H	6.54260900	0.62852500	1.05440100
C	6.08259700	-0.96578900	-1.76877200

H	6.58992800	0.00521800	-1.70943100
H	6.51053800	-1.51435000	-2.62134800
H	6.30507100	-1.53320200	-0.85230000
C	6.25319500	3.73957500	-1.24670100
H	6.16729700	4.61397900	-1.89560100
C	-1.16152000	2.89034600	0.49923600
H	-0.97366800	2.68894100	1.56471200
H	-0.74671200	3.88037400	0.25967600
H	-0.62146000	2.12527100	-0.08281300
C	1.37801300	-2.69099000	3.25502300
H	2.29852800	-3.18997900	3.54743400
C	-2.89066500	3.20937100	-1.27165800
H	-2.40730200	2.46802000	-1.92460400
H	-2.46723000	4.19990600	-1.50151400
H	-3.96121900	3.22703700	-1.51708300
C	1.38841700	-4.16010400	-0.66751500
H	1.21526000	-4.37976600	0.39731900
H	2.39419300	-3.73774500	-0.75974600
H	1.32469600	-5.09909300	-1.23956400
C	5.27298200	1.74993200	-0.28247400
C	1.67320300	1.52212600	2.56748400
H	0.82210000	1.18183200	1.95960900
H	1.31820800	2.29538000	3.26355900
H	2.03041400	0.66335100	3.15457700
C	-2.65904700	2.85506900	0.20100700
C	-7.27881200	1.57912100	-2.12121300
H	-7.70473800	1.23312100	-3.06772300
C	3.98645700	2.44693100	2.56048400

H	4.39613000	1.55892300	3.06390400
H	3.65614600	3.15168800	3.33804200
H	4.79062200	2.93162500	1.99116400
C	-3.31739900	3.91855200	1.09081100
H	-4.35957200	4.10221500	0.80624600
H	-2.77488200	4.87280400	1.00098300
H	-3.28992500	3.60235600	2.14453700
C	-6.09565200	1.01921500	-1.67067900
H	-5.58802500	0.26927900	-2.27837700
C	7.43892200	3.49303900	-0.55718900
H	8.28543900	4.17510400	-0.66385000
C	-2.54259300	0.85948000	4.11473900
H	-1.65308700	0.53822400	4.67802300
H	-3.33428800	1.11102800	4.83742500
H	-2.28427200	1.77101700	3.55607900
C	4.44274800	-1.56723000	2.70187900
H	4.68566900	-0.54920600	2.36786300
H	5.38446600	-2.08712600	2.93788700
H	3.85832500	-1.48040500	3.62959300

4.S$_2$

PBE0-D3BJ/Def2-SVP~ma-TZVP

E = -4551.112963 a.u.

Mn	-0.14039300	-0.53819400	-0.60546100
P	-0.76300000	-0.35960200	-3.05933400
Si	-2.22889000	-0.05889700	0.64387300
Si	2.18200600	-0.35807000	-0.07886300
P	-0.27811000	-2.94844800	-1.13896000
N	0.26790600	-1.39909500	1.70043900

N	-1.88669100	-0.73305800	2.29094700
N	-3.98861600	-0.29970200	0.26084100
N	3.88150100	-0.19241200	-0.90387800
N	2.44196200	-1.77694400	1.03295500
N	-3.04771500	1.55794600	0.92529500
N	3.28714600	0.95278100	0.81654100
C	3.70035400	-2.48408400	1.15710500
H	3.50529900	-3.56533700	1.27214500
H	4.23872100	-2.37451900	0.20507200
C	2.62789000	3.20167600	1.49993000
H	3.39642600	3.72899400	0.91699200
H	2.36272300	3.83345000	2.36112700
H	1.73380100	3.07656200	0.87513400
C	-5.31551900	1.78687000	-0.24367600
C	3.88807300	0.72072000	-3.18426100
H	2.81014500	0.90922600	-3.06751200
H	4.09271700	0.49474900	-4.24213100
H	4.43295000	1.64020200	-2.92677400
C	-4.92937100	-1.41979400	0.24534500
C	7.81012600	1.96153100	0.16523500
H	8.76005300	1.57027300	0.53623700
C	-1.07370500	-3.13236000	-2.80247400
H	-0.90469300	-4.14649100	-3.20287800
H	-2.15862700	-3.02633100	-2.63953900
C	-2.81530600	-0.47536000	3.37739800
H	-3.18725000	-1.41757400	3.81847900
H	-3.69192100	0.02131900	2.93669400
C	4.22312600	0.83465300	-0.10200800

C	-1.32646800	-4.02914000	-0.08551800
H	-1.34517000	-5.06691000	-0.45347800
H	-2.34984300	-3.63610400	-0.05857900
H	-0.92987400	-4.01713900	0.94121100
C	-2.53842000	0.00640800	-3.36427800
H	-2.73834300	1.05937000	-3.11807100
H	-3.14642000	-0.59912500	-2.67925100
H	-2.83523300	-0.18541000	-4.40767700
C	0.02464800	0.64148400	-4.39153900
H	-0.43314500	0.45526500	-5.37650000
H	1.09727300	0.40600600	-4.44160700
H	-0.07977800	1.70923700	-4.14759600
C	-4.89892100	-2.11134700	-1.12452700
H	-5.23164000	-1.42943100	-1.91886600
H	-5.55488600	-2.99587600	-1.13960600
H	-3.87435400	-2.43771800	-1.35955500
C	-4.50455700	-2.43998800	1.31027700
H	-3.42931800	-2.65629300	1.26214700
H	-5.05498000	-3.38440500	1.18087300
H	-4.71961300	-2.05879900	2.31712900
C	-7.51394700	3.16978900	-1.43487000
H	-8.35330200	3.69990500	-1.88920600
C	-0.74074600	-2.45734100	3.58888600
H	-1.58226600	-2.57543700	4.26840000
C	3.58769000	-1.71115600	-2.73403500
H	3.89773000	-2.57524300	-2.12774700
H	3.81352800	-1.92888500	-3.78829200
H	2.49954400	-1.58234500	-2.61561500

C	-0.58272000	-2.06410400	-3.77110000
H	-1.11673100	-2.12163000	-4.73551700
H	0.49031100	-2.20147900	-3.98802700
C	4.31221500	-0.44488900	-2.28515800
C	-0.78814100	-1.52671200	2.52598800
C	-7.02314000	3.54524200	-0.17448700
H	-7.50306500	4.36228600	0.37243000
C	-4.23327400	1.06639500	0.34038600
C	-5.95802800	2.88078600	0.40887100
H	-5.64480100	3.14960500	1.41785800
C	3.13861800	1.83743100	1.97297500
C	5.37918800	2.95983500	-0.77010000
H	4.42630800	3.34872700	-1.13497800
C	1.40120400	-2.10443200	1.87284100
C	-6.36129900	-0.99341800	0.57968800
H	-6.38389800	-0.41189700	1.51298900
H	-6.97847100	-1.89478400	0.71823300
H	-6.81814200	-0.38098300	-0.20662900
C	0.39765300	-3.23763100	3.72774200
H	0.44269000	-3.98029600	4.52916900
C	6.66418100	1.17612100	0.23819200
H	6.70895100	0.17117500	0.66363400
C	5.82091500	-0.67674800	-2.38597900
H	6.39828700	0.23629300	-2.19249200
H	6.07102200	-1.02196300	-3.40057700
H	6.14292500	-1.45094700	-1.67307200
C	6.53122000	3.73972800	-0.85029700
H	6.47727300	4.74225700	-1.28059900

C	-1.00164800	2.77114800	1.22868500
H	-1.06251600	2.63086400	2.31797100
H	-0.45093100	3.70162500	1.02769700
H	-0.42069600	1.93141700	0.80866400
C	1.49036000	-3.08693000	2.87969900
H	2.39094200	-3.67910500	3.02754400
C	-2.26350100	3.04389600	-0.90601100
H	-1.64701400	2.23880800	-1.33909900
H	-1.78031500	4.00764000	-1.13221000
H	-3.25153100	3.02936600	-1.38956500
C	1.18584800	-4.06386500	-1.29674300
H	1.73690300	-4.05159500	-0.34562100
H	1.85592500	-3.69026700	-2.08111700
H	0.88870800	-5.09835000	-1.53172100
C	5.44075700	1.67317200	-0.22613000
C	2.09742300	1.18152200	2.88166300
H	1.16317300	0.98309500	2.33694100
H	1.86962600	1.83795400	3.73376100
H	2.46682100	0.22154300	3.27036700
C	-2.39708800	2.83798300	0.60812400
C	-6.90842300	2.08664600	-2.08874900
H	-7.26651900	1.77851700	-3.07568200
C	4.44346900	2.01227900	2.75222400
H	4.86220100	1.03918000	3.04791900
H	4.23817900	2.58228700	3.67057200
H	5.20325100	2.56199600	2.18108400
C	-3.11232700	4.04023300	1.23166400
H	-4.03631400	4.29095400	0.69815200

H	-2.45429300	4.92256700	1.19734400
H	-3.35743500	3.83185900	2.28433800
C	-5.84767700	1.40735300	-1.51457500
H	-5.36909500	0.59217200	-2.05951700
C	7.74631600	3.24428100	-0.38029600
H	8.64716100	3.85929200	-0.43888500
C	-2.22793600	0.42052000	4.45581900
H	-1.32245300	-0.02027500	4.90080000
H	-2.95747900	0.59010500	5.26292600
H	-1.95954700	1.39565100	4.02412600
C	4.59579100	-1.99445000	2.28745300
H	4.88113100	-0.94592100	2.12531200
H	5.51594600	-2.59699900	2.34504700
H	4.08826900	-2.05315000	3.26185200

5.doublet

PBE0-D3BJ/Def2-SVP~ma-TZVP

E = -3970.459136 a.u.

Mn	-0.11958300	-0.19864200	-1.44389100
Si	-1.60288300	0.29451100	0.19298100
Si	1.69450300	0.80083300	-0.33584000
N	-1.49227200	1.42870300	1.60399200
N	0.57732800	2.16433200	0.89537100
O	-2.37754500	-1.64974600	-2.61780400
N	-3.35998400	0.71945200	-0.36706900
N	-2.82995500	-0.98850300	0.80965400
N	2.62485000	-0.12048500	1.04090700
N	3.39905900	0.03829500	-0.97785600
N	2.40476800	2.50806900	-0.20518600

O	0.67357700	0.57146300	-4.18340400
C	0.68857900	-1.69600300	-0.97681900
C	-6.19345100	-0.78472100	0.76373400
H	-5.99636800	-0.19686700	1.66307000
C	-3.94550500	1.77652200	-1.18990100
O	1.21840500	-2.68022100	-0.63906900
C	-1.49576400	-1.04280400	-2.15540800
C	-5.41078400	-1.75908900	-1.31121800
H	-4.59979400	-1.92871800	-2.02261300
C	-0.55313400	2.43025300	1.55547900
C	-3.81800900	-0.43514500	0.09184600
C	1.49660200	3.09876600	0.59884000
C	-2.60319100	1.58686000	2.52645200
H	-3.36344600	0.84147100	2.26109100
H	-3.08536300	2.57244700	2.38879500
C	2.51228300	-0.44665100	2.47432900
C	1.41072300	4.39773300	1.12529000
H	2.16504900	5.15563200	0.91996400
C	-0.70496200	3.70967600	2.13255800
H	-1.58379400	3.96547100	2.71996000
C	0.42518800	0.28562800	-3.08829800
C	-7.70923300	-2.05550900	-0.61944800
H	-8.70548300	-2.46530400	-0.80130100
C	3.58154100	-0.53446500	0.19683400
C	0.29389800	4.65884900	1.91335600
H	0.17711900	5.65214100	2.35575700
C	-5.16358500	-1.00985500	-0.15619800
C	3.62909300	3.16054100	-0.58481200

H	4.34137000	2.37913800	-0.88286000
H	4.06947300	3.65073500	0.30451700
C	4.65669700	-1.51724400	0.50469800
C	-2.65500200	-3.40428600	0.31030900
H	-3.58669300	-3.42711300	-0.27137800
H	-2.51778900	-4.39458700	0.77037600
H	-1.81894100	-3.22445900	-0.37887000
C	-6.68364400	-2.28069100	-1.53598300
H	-6.87263300	-2.86756000	-2.43762800
C	-2.94332100	2.93260000	-1.15250400
H	-2.82435700	3.32596300	-0.13199600
H	-3.28217800	3.75207900	-1.80231000
H	-1.95516100	2.59744200	-1.50869500
C	-4.12284700	1.30764600	-2.63562000
H	-3.16021700	0.98685800	-3.05692700
H	-4.51728700	2.13012600	-3.25140800
H	-4.82712200	0.46675100	-2.69925900
C	1.25509200	0.20848900	3.03208700
H	1.35035000	1.30077500	3.05897900
H	1.08822000	-0.14598400	4.05960600
H	0.37161700	-0.04146400	2.43443200
C	-7.46296200	-1.30569600	0.53069400
H	-8.26402600	-1.12554000	1.25108500
C	3.78904900	0.95883700	-3.18027200
H	2.74348100	1.28328100	-3.13953100
H	4.01412700	0.69284100	-4.22297300
H	4.42949500	1.80808900	-2.90635800
C	2.39933800	-1.96075900	2.69544600

H	1.62696300	-2.38906800	2.04203600
H	2.12592400	-2.16269200	3.74208100
H	3.34356300	-2.48105300	2.49470000
C	-2.20477600	1.38670600	3.98001900
H	-1.82208700	0.36748100	4.13716400
H	-3.07037600	1.53520300	4.64397000
H	-1.41579700	2.08951800	4.28600700
C	4.04851400	-0.24316800	-2.26848300
C	6.92012100	-2.00101100	1.20725300
H	7.88861700	-1.65403500	1.57459200
C	6.70956200	-3.35682400	0.95905000
H	7.51294400	-4.07618200	1.13441300
C	-2.70105600	-2.32663700	1.39573400
C	-1.37554200	-2.32184400	2.15293500
H	-0.54806300	-2.07523800	1.47216300
H	-1.17667200	-3.31257200	2.58571400
H	-1.38884700	-1.58392300	2.96800800
C	3.70876100	0.11675300	3.24942700
H	4.64305000	-0.40316700	3.00290000
H	3.54038200	0.00139500	4.33122400
H	3.83037700	1.18909300	3.03329600
C	-5.28183700	2.24326900	-0.61008300
H	-6.05691600	1.46930000	-0.69361500
H	-5.63291000	3.13148300	-1.15631900
H	-5.17007600	2.51481200	0.45092300
C	-3.83923300	-2.60538400	2.37953300
H	-3.92158100	-1.79347600	3.11870600
H	-3.63673700	-3.54137400	2.92137000

H	-4.80607500	-2.71615200	1.87097300
C	5.89825300	-1.08333000	0.97829900
H	6.06852900	-0.01846100	1.15057200
C	3.46208000	4.18093800	-1.70212000
H	3.04519000	3.70744300	-2.60228200
H	4.43000000	4.63496300	-1.96610800
H	2.77725100	4.99001100	-1.40687700
C	4.44499500	-2.87835000	0.26056200
H	3.46820800	-3.20982200	-0.09751700
C	5.47194500	-3.79204600	0.48652800
H	5.30117100	-4.85343600	0.29260900
C	3.45653500	-1.50619400	-2.90212300
H	3.63110600	-2.38365600	-2.26533400
H	3.92925800	-1.69208800	-3.87844200
H	2.37493600	-1.40217900	-3.05147800
C	5.56926500	-0.40369600	-2.15592600
H	6.01428600	0.41591800	-1.57114200
H	5.99861500	-0.36618400	-3.16862400
H	5.86930700	-1.35725800	-1.70483900

5.quartet

PBE0-D3BJ/Def2-SVP~ma-TZVP

E = -3970.405301 a.u.

Mn	-0.06558800	0.00862000	-1.40687200
Si	-1.77972600	0.21041600	0.25805300
Si	1.78524800	0.82190800	-0.21586900
N	-1.42289900	1.26769600	1.70512700
N	0.66978900	2.03749600	1.00812600
O	-1.26219900	-1.06477000	-3.98674500

N	-3.39950900	0.59621100	-0.40135500
N	-2.89803900	-1.04523100	0.95117700
N	2.70880400	-0.25865500	0.99396400
N	3.31928400	0.01824900	-1.06062700
N	2.49467800	2.48190500	-0.05868600
O	-0.26534700	2.89086300	-2.08385300
C	0.52417700	-1.58941600	-0.82961700
C	-6.06460700	-1.75011100	0.76472300
H	-5.80015500	-1.68058800	1.81986000
C	-4.05374600	1.81305600	-0.88846300
O	0.98471000	-2.59934300	-0.49213600
C	-0.78499000	-0.65579600	-3.03164300
C	-5.63720100	-1.20794300	-1.55748000
H	-4.97828800	-0.81553100	-2.33323500
C	-0.47029800	2.24358700	1.69529900
C	-3.95188400	-0.56348200	0.14330300
C	1.57957200	3.01507700	0.78637600
C	-2.46050600	1.34870000	2.73184400
H	-3.23828000	0.62748400	2.45088400
H	-2.93908900	2.34340500	2.72563900
C	2.72460100	-0.58983700	2.43296800
C	1.49124900	4.26396700	1.40003100
H	2.23924400	5.03857100	1.24141000
C	-0.60274400	3.48103900	2.37042600
H	-1.48139100	3.68613300	2.97595900
C	-0.23029200	1.76703700	-1.81456600
C	-7.69388200	-2.33179300	-0.93716200
H	-8.65336300	-2.77179100	-1.21640500

C	3.57322100	-0.66027500	0.04715900
C	0.37645100	4.45357200	2.21673900
H	0.25000800	5.40982600	2.73148600
C	-5.19038300	-1.18491100	-0.20517700
C	3.67493300	3.17168500	-0.50238700
H	4.35159800	2.41622800	-0.92822200
H	4.20351400	3.59643600	0.37207300
C	4.61965200	-1.70667800	0.19334100
C	-2.47921300	-3.25948300	-0.07269700
H	-3.45270200	-3.26675700	-0.58467200
H	-2.18705700	-4.30148200	0.13008800
H	-1.73935500	-2.82150200	-0.75678600
C	-6.85445800	-1.76985700	-1.90751800
H	-7.15041200	-1.78852900	-2.96028800
C	-3.29765800	3.02542000	-0.33182600
H	-3.38642300	3.07652600	0.76210100
H	-3.71363100	3.95423800	-0.74984200
H	-2.23121800	3.00172000	-0.59287800
C	-3.99281100	1.87402600	-2.42033300
H	-2.94904100	1.82775400	-2.76399800
H	-4.43338600	2.81234800	-2.79175400
H	-4.54222300	1.03963100	-2.87501900
C	1.53627000	0.09000900	3.10431100
H	1.64431200	1.18180700	3.10867900
H	1.47226900	-0.24796900	4.14827500
H	0.59012700	-0.16157900	2.60809200
C	-7.28013600	-2.30774100	0.40150900
H	-7.93431000	-2.71177800	1.17942400

C	3.10229000	0.87504900	-3.29655300
H	2.00581000	0.81653600	-3.20969000
H	3.36887600	0.73600900	-4.35375500
H	3.41723200	1.88262500	-2.99516400
C	2.59889300	-2.10023300	2.65978400
H	1.73691200	-2.50379700	2.11088800
H	2.44939100	-2.29648200	3.73169400
H	3.49784800	-2.64461400	2.34689800
C	-1.94576300	1.01002100	4.12009800
H	-1.57759500	-0.02580000	4.14992400
H	-2.75325600	1.10617000	4.86190300
H	-1.12105900	1.67103800	4.42794700
C	3.77142400	-0.20105100	-2.44180000
C	6.92884600	-2.30866800	0.59030900
H	7.95572900	-2.01238900	0.81550000
C	6.61007600	-3.65389900	0.41178400
H	7.38789100	-4.41609100	0.49790900
C	-2.57223500	-2.45077800	1.22562900
C	-1.23178400	-2.42723900	1.96210700
H	-0.47918900	-1.84877100	1.41147900
H	-0.83945800	-3.44600000	2.09407100
H	-1.35041800	-1.96769400	2.95453700
C	4.00925200	-0.05569600	3.07341000
H	4.90018200	-0.57862300	2.70061300
H	3.97165300	-0.19730900	4.16405900
H	4.11819000	1.02058300	2.87039700
C	-5.50402200	1.91047700	-0.40881700
H	-6.14841300	1.14568500	-0.85920500

H	-5.90696000	2.89959600	-0.67518100
H	-5.55832500	1.79752300	0.68441700
C	-3.57524200	-3.12642700	2.16594800
H	-3.79448400	-2.47735200	3.02771600
H	-3.14394500	-4.06603400	2.54466600
H	-4.51573000	-3.37174000	1.65951600
C	5.93806800	-1.33625600	0.47613800
H	6.18766900	-0.28043100	0.60213700
C	3.39707200	4.26805100	-1.52148500
H	2.88137100	3.86178800	-2.40371600
H	4.33477500	4.74135500	-1.85054800
H	2.74891100	5.05160400	-1.10278800
C	4.30113700	-3.05763300	0.01661200
H	3.26871400	-3.34102700	-0.19629000
C	5.29741100	-4.02499500	0.12275400
H	5.04304700	-5.07784700	-0.01781900
C	3.33784200	-1.57321800	-2.96407300
H	3.80082200	-2.39053600	-2.39615900
H	3.63794200	-1.68296000	-4.01695600
H	2.24535000	-1.67915200	-2.90599400
C	5.29098300	-0.05182600	-2.55736100
H	5.62632500	0.90254700	-2.12335800
H	5.58215200	-0.06267300	-3.61824000
H	5.82421900	-0.86925600	-2.05511900

5.S$_2$

PBE0-D3BJ/Def2-SVP~ma-TZVP

E = -3970.299413 a.u.

Mn	-0.05863400	-0.00936000	-1.44826600

Si	-1.77132400	0.22916000	0.20019300
Si	1.72132100	0.81613600	-0.15392200
N	-1.45647800	1.35399300	1.62574900
N	0.72248200	1.97889000	0.99840000
O	-1.19271300	-0.97895300	-4.09590500
N	-3.39780600	0.60384500	-0.44238200
N	-2.88032100	-1.00539300	0.94581800
N	2.68835800	-0.27097100	1.01107500
N	3.27725700	0.00709300	-1.05019700
N	2.52005300	2.48391500	-0.04730900
O	-0.19264600	2.89754000	-2.04784100
C	0.51833900	-1.61454500	-0.87136700
C	-6.05497600	-1.68216600	0.85745700
H	-5.76413400	-1.57888800	1.90304500
C	-4.04811900	1.80258900	-0.97849600
O	0.96911800	-2.62466900	-0.52321800
C	-0.74352400	-0.61285700	-3.10998100
C	-5.67656500	-1.22697500	-1.49167000
H	-5.03397700	-0.86542600	-2.29572200
C	-0.42044000	2.22910300	1.70997900
C	-3.94964900	-0.53554800	0.15014700
C	1.64588900	2.99934900	0.83411800
C	-2.53755900	1.46150600	2.60291200
H	-3.37484100	0.86228300	2.22579400
H	-2.89970600	2.50162900	2.65840200
C	2.71920400	-0.59842200	2.44995300
C	1.62451300	4.19386000	1.55079700
H	2.39710500	4.94509700	1.38003600

C	-0.47093800	3.41821300	2.47830200
H	-1.38358700	3.63297400	3.03247300
C	-0.17628300	1.76437700	-1.81274200
C	-7.72523300	-2.31379600	-0.78533400
H	-8.69329700	-2.75715200	-1.02707900
C	3.54270200	-0.67044300	0.05126900
C	0.58095600	4.38310000	2.49271600
H	0.50346300	5.28537300	3.09448100
C	-5.20008200	-1.15795100	-0.15144900
C	3.69866300	3.15088900	-0.52351700
H	4.33821200	2.38504700	-0.98474000
H	4.26290600	3.55433100	0.33721400
C	4.58927700	-1.71874100	0.19087300
C	-2.50057600	-3.22870800	-0.07140900
H	-3.48528000	-3.23246100	-0.56137500
H	-2.21093300	-4.27147100	0.13067100
H	-1.77331800	-2.79925400	-0.77445800
C	-6.90490100	-1.79308300	-1.79385600
H	-7.22494600	-1.84735600	-2.83827100
C	-3.29767600	3.03676500	-0.46340300
H	-3.40607800	3.13769900	0.62524700
H	-3.70523600	3.94628700	-0.92932600
H	-2.22721400	2.99978200	-0.70457600
C	-3.97108000	1.80202500	-2.51074100
H	-2.92244600	1.75702700	-2.84008000
H	-4.42087600	2.71754400	-2.92540500
H	-4.50196500	0.94072100	-2.93677700
C	1.52919400	0.06643300	3.13827100

H	1.61535800	1.15979200	3.13709700
H	1.49130000	-0.27184300	4.18361700
H	0.57951400	-0.20518100	2.65986100
C	-7.28157000	-2.24458500	0.54209300
H	-7.92009800	-2.61678900	1.34831100
C	3.06772300	0.86872700	-3.28781800
H	1.97146300	0.80673200	-3.20565300
H	3.34010300	0.73077300	-4.34374500
H	3.37681900	1.87803900	-2.98614100
C	2.61406800	-2.11012200	2.67975200
H	1.75374900	-2.52451200	2.13588900
H	2.47326300	-2.30776200	3.75265600
H	3.51746500	-2.64381500	2.36135100
C	-2.14280400	0.95502700	3.97890400
H	-1.88087800	-0.11124200	3.92885600
H	-2.98245500	1.06762000	4.68132000
H	-1.27791300	1.50188500	4.38443400
C	3.73552200	-0.20595600	-2.42994700
C	6.89800000	-2.32454900	0.58987100
H	7.92473500	-2.02947200	0.81745100
C	6.57890000	-3.66903900	0.40668800
H	7.35600700	-4.43208000	0.49167700
C	-2.55847100	-2.41242100	1.22461100
C	-1.20207100	-2.40101500	1.93129500
H	-0.46129400	-1.81147100	1.37647900
H	-0.80701200	-3.42206300	2.03340000
H	-1.29598900	-1.96458700	2.93655000
C	4.00232700	-0.04767600	3.07961200

H	4.89649200	-0.55679900	2.69561400
H	3.97885200	-0.19221600	4.17030500
H	4.09336900	1.03072800	2.87877700
C	-5.50258900	1.92018400	-0.51636400
H	-6.14704500	1.14470800	-0.94750900
H	-5.89751500	2.90216000	-0.81842000
H	-5.56905300	1.84348900	0.57929400
C	-3.54712000	-3.07358500	2.18999200
H	-3.74172700	-2.41732300	3.05225200
H	-3.11559800	-4.01454400	2.56510100
H	-4.50074200	-3.31352700	1.70601200
C	5.90787300	-1.35123000	0.47734600
H	6.15869800	-0.29609700	0.60678800
C	3.39430700	4.26483000	-1.51663500
H	2.82607800	3.87935800	-2.37540600
H	4.32654400	4.71775700	-1.88601900
H	2.78621000	5.05539600	-1.05374000
C	4.27095200	-3.06932800	0.00986800
H	3.23865000	-3.35192200	-0.20423300
C	5.26622800	-4.03781700	0.11455400
H	5.01101400	-5.09003500	-0.02942100
C	3.31252800	-1.57769300	-2.96283900
H	3.77096000	-2.39589400	-2.39271200
H	3.62556600	-1.68221100	-4.01254900
H	2.21992900	-1.68764700	-2.91902100
C	5.25566300	-0.05184100	-2.54139500
H	5.58959600	0.89978500	-2.10013700
H	5.54895500	-0.05401700	-3.60179200

H	5.79020400	-0.87133200	-2.04415300

Mn(CO)$_5$.doublet

PBE0-D3BJ/Def2-SVP~ma-TZVP

E = -1716.462915 a.u.

Mn	0.00000000	0.00000000	0.21024700
C	0.00000000	0.00000000	-1.60024300
O	0.00000000	0.00000000	-2.74491000
C	0.00000000	1.83793200	0.41172600
C	-1.83793200	0.00000000	0.41172600
C	1.83793200	0.00000000	0.41172600
C	0.00000000	-1.83793200	0.41172600
O	0.00000000	2.97509100	0.51322400
O	2.97509100	0.00000000	0.51322400
O	0.00000000	-2.97509100	0.51322400
O	-2.97509100	0.00000000	0.51322400

Mn(CO)$_5$.quartet.partially optimized

PBE0-D3BJ/Def2-SVP~ma-TZVP

E = -1716.376354 a.u.

C	0.00100800	-1.60887900	1.44465200
O	0.00129600	-1.30308500	2.54931000
C	0.00022200	-0.87650400	-1.66054300
C	-1.82360400	-0.00542400	0.11476200
C	1.82319600	-0.00419600	0.11433900
O	0.00058200	-1.49409100	-2.61927600
O	2.96646000	-0.00870300	0.15939200
O	-2.96685600	-0.01063200	0.16005500
Mn	-0.00025300	0.06938700	-0.08695300
C	-0.00064900	1.90507700	-0.05319300

O	-0.00082300	3.04212100	0.05223400

$Mn(CO)_5$.sextet.partially optimized

PBE0-D3BJ/Def2-SVP~ma-TZVP

E = -1716.254660 a.u.

C	1.16047200	0.00124000	-1.16214900
O	1.90606400	0.00345300	-2.07237300
C	1.54823800	-0.00515600	0.43723700
C	-0.45923200	1.69720700	0.69387900
C	-0.46779900	-1.69758300	0.68820400
O	2.60371400	-0.01094800	0.95930500
O	-0.52049800	-2.76811100	1.06918100
O	-0.50695400	2.76659600	1.07866900
Mn	-0.24179400	0.00036700	0.02453400
C	-1.68254700	0.01055300	-1.11506900
O	-2.80106900	0.00316800	-0.76802700

8 References:

[1] Gallego, D.; Inoue, S.; Blom, B.; Driess, M. *Organometallics* **2014,** *33* (23), 6885–6897.

[2] Bain, G. A.; Berry, J. F. *J. Chem. Educ.* **2008**, *85*, 532.

[3] Bill, E. *SQUID Program julX2*, **2019**.

[4] Bill, E. *EPR Program eview*, **2019**.

[5] Bill, E. *EPR Program esim*, **2019.**

[6] Gaffney, B. J.; Silverstone J. J., *Simulation of the EMR Spectra of High-Spin Iron in Proteins* (Plenum Press, New York, **1993**).

[7] Sheldrick, G. M. SHELX-97 Program for Crystal Structure Determination, Universität Göttingen, Germany (**1997**).

[8] Espinal-Viguri, M.; S. E. Neale, N. T. Coles, S. A. Macgregor, and R. L. Webster, *Journal of the American Chemical Society* **2019,** *141* (1), 572–582.

[9] Frisch, M. J.; Trucks, G. W.; Schlegel, H. B.; Scuseria, G. E.; Robb, M. A.; Cheeseman, J. R.; Scalmani, G.; Barone, V.; Mennucci, B.; Petersson, G. A.; Nakatsuji, H.; Caricato, M.; Li, X.; Hratchian, H. P.; Izmaylov, A. F.; Bloino, J.; Zheng, G.; Sonnenberg, J. L.; Hada, M.; Ehara, M.; Toyota, K.; Fukuda, R.; Hasegawa, J.; Ishida, M.; Nakajima, T.; Honda, Y.; Kitao, O.; Nakai, H.; Vreven, T.; Montgomery, J. A., Jr.; Peralta, J. E.; Ogliaro, F.; Bearpark, M.; Heyd, J. J.; Brothers, E.; Kudin, K. N.; Staroverov, V. N.; Kobayashi, R.; Normand, J.; Raghavachari, K.;

Rendell, A.; Burant, J. C.; Iyengar, S. S.; Tomasi, J.; Cossi, M.; Rega, N.; Millam, J. M.; Klene, M.; Knox, J. E.; Cross, J. B.; Bakken, V.; Adamo, C.; Jaramillo, J.; Gomperts, R.; Stratmann, R. E.; Yazyev, O.; Austin, A. J.; Cammi, R.; Pomelli, C.; Ochterski, J. W.; Martin, R. L.; Morokuma, K.; Zakrzewski, V. G.; Voth, G. A.; Salvador, P.; Dannenberg, J. J.; Dapprich, S.; Daniels, A. D.; Farkas, O.; Foresman, J. B.; Ortiz, J. V.; Cioslowski, J.; Fox, D. J., Gaussian 16, Revision A.03; Gaussian, Inc., Wallingford CT, **2016**.

[10] Adamo C., Barone. V. Toward reliable density functional methods without adjustableparameters: The PBE0 model. *J. Chem. Phys.* **1999**, *110*, 6158–6169.

[11] Grimme S., Ehrlich S., Goerigk L., Effect of the damping function in dispersion corrected density functional theory. *J. Comp. Chem.* **2011**, *32*, 1456–1465.

[12] Weigend F., Ahlrichs. R., Balanced basis sets of split valence, triple zeta valence and quadruple zeta valence quality for H to Rn: Design and assessment of accuracy. *Phys. Chem. Chem. Phys.* **2005**, *7*, 3297–305.

[13] Zheng, J.; Xu, X.; Truhlar, D. G. Minimally augmented Karlsruhe basis sets. *Theor. Chem. Acc.* **2010**, *128*, 295–305.

[14] Papajak, E.; Zheng, J.; Xu, X.; Leverentz, H. R.; Truhlar, D. G. Perspectives on Basis Sets Beautiful: Seasonal Plantings of Diffuse Basis Functions. *J. Chem. Theory. Comput.* **2011**, *7*, 3027–3034.

[15] Zhang, J.-X.; Sheong, F. K.; Lin, Z. Unravelling Chemical Interactions with Principal Interacting Orbital Analysis. *Chem. Eur. J.* **2018**, *24*, 9639–9650.

[16] Zhang, J.-X.; Sheong, F. K.; Lin, Z. Principal interacting orbital: A chemically intuitive method for deciphering bonding interaction. *WIREs Comput. Mol. Sci.* **2020**, e1469.

[17] Sheong, F. K.; Zhang, J.-X.; Lin, Z., Principal interacting spin orbital: understanding the fragment interactions in open-shell systems. *Phys. Chem. Chem. Phys.* **2020**, *22*, 10076–10086.

[18] SGlendening, E. D., Badenhoop, J. K., Reed, A. E., Carpenter, J. E., Bohmann, J. A., Morales, C. M., Landis, C. R., Weinhold, F., NBO 7.0; Theoretical Chemistry Institute, University of Wisconsin: Madison, WI, **2013**. http://nbo7.chem.wisc.edu/.

[19] Lu, T.; Chen, F., Multiwfn: A multifunctional wavefunction analyzer. *J. Comput. Chem.* **2012**, *33*, 580–592.

[20] Humphrey, W.; Dalke, A.; Schulten, K., VMD: Visual molecular dynamics. *J. Mol. Graph.* **1996**, *14*, 33–38.
